# BDDE-Inspired Chalcone Derivatives to Fight Bacterial and Fungal Infections

**DOI:** 10.3390/md20050315

**Published:** 2022-05-08

**Authors:** Ana Jesus, Fernando Durães, Nikoletta Szemerédi, Joana Freitas-Silva, Paulo Martins da Costa, Eugénia Pinto, Madalena Pinto, Gabriella Spengler, Emília Sousa, Honorina Cidade

**Affiliations:** 1 Laboratory of Organic and Pharmaceutical Chemistry, Department of Chemical Sciences, Faculty of Pharmacy, University of Porto, Rua Jorge Viterbo Ferreira 228, 4050-313 Porto, Portugal; anaaimjesus@gmail.com (A.J.); fduraes5@gmail.com (F.D.); madalena@ff.up.pt (M.P.); 2 CIIMAR—Interdisciplinary Centre of Marine and Environmental Research, University of Porto, Avenida General Norton de Matos, 4450-208 Matosinhos, Portugal; joanafreitasdasilva@gmail.com (J.F.-S.); pmcosta@icbas.up.pt (P.M.d.C.); epinto@ff.up.pt (E.P.); 3 Department of Medical Microbiology, Albert Szent-Györgyi Health Center and Albert Szent-Györgyi Medical School, University of Szeged, Semmelweis utca 6, 6725 Szeged, Hungary; szemeredi.nikoletta@med.u-szeged.hu (N.S.); spengler.gabriella@med.u-szeged.hu (G.S.); 4 ICBAS—Institute of Biomedical Sciences Abel Salazar, University of Porto, Rua de Jorge Viterbo Ferreira 228, 4050-313 Porto, Portugal; 5 Laboratory of Microbiology, Department of Biological Sciences, Faculty of Pharmacy, University of Porto, Rua de Jorge Viterbo Ferreira 228, 4050-313 Porto, Portugal

**Keywords:** antibiotic resistance, BDDE, halogenated chalcone derivatives, antimicrobial activity, EPs inhibitors

## Abstract

The growing number of infectious diseases around the world threatens the effective response of antibiotics, contributing to the increase in antibiotic resistance seen as a global health problem. Currently, one of the main challenges in antimicrobial drug discovery is the search for new compounds that not only exhibit antimicrobial activity, but can also potentiate the antimicrobial activity and revert antibiotics’ resistance, through the interference with several mechanisms, including the inhibition of efflux pumps (EPs) and biofilm formation. Inspired by macroalgae brominated bromophenol BDDE with antimicrobial activity, a series of 18 chalcone derivatives, including seven chalcones (**9**–**15**), six dihydrochalcones (**16**–**18**, and **22**–**24**) and five diarylpropanes (**19**–**21**, and **25** and **26**), was prepared and evaluated for its antimicrobial activity and potential to fight antibiotic resistance. Among them, chalcones **13** and **14** showed promising antifungal activity against the dermatophyte clinical strain of *Trichophyton rubrum*, and all compounds reversed the resistance to vancomycin in *Enterococcus faecalis* B3/101, with **9**, **14**, and **24** able to cause a four-fold decrease in the MIC of vancomycin against this strain. Compounds **17**–**24** displayed inhibition of EPs and the formation of biofilm by *S. aureus* 272123, suggesting that these compounds are inhibiting the EPs responsible for the extrusion of molecules involved in biofilm-related mechanisms. Interestingly, compounds **17**–**24** did not show cytotoxicity in mouse embryonic fibroblast cell lines (NIH/3T3). Overall, the results obtained suggest the potential of dihydrochalcones **16**–**18** and **22**–**24,** and diarylpropanes **19**–**21**, **25** and **26**, as hits for bacterial EPs inhibition, as they are effective in the inhibition of EPs, but present other features that are important in this matter, such as the lack of antibacterial activity and cytotoxicity.

## 1. Introduction

Bacterial resistance to antibiotics is a major cause of antimicrobial treatment failure, which poses an increasingly concerning health problem [1,2,3]. This phenomenon can arise through several mechanisms, including target modification and/or overproduction, membrane impermeabilization, drug inactivation, and drug extrusion by efflux pumps (EPs) [4,5]. Therefore, the need for the discovery of new antimicrobial drugs, as well as antimicrobial adjuvants that can revert these resistance mechanisms, is urgent.

EPs, in particular, acquire increasing importance, as they can lead to multidrug resistance. These membrane structures are ubiquitous to every bacterium and are divided into different families [3]. Furthermore, they are involved in different resistance phenomena, such as the formation of biofilm. In fact, research has been carried out in order to explore a possible connection between the formation of biofilm and EPs, and, while no causative or genetic relationship has yet been found, it was noted that EP inhibitors are able to affect biofilm formation in *Staphylococcus aureus*, either by leading to the formation of defective biofilm or in a reduction of its production altogether [6]. It is also known that EPs play an important role in the efflux of extracellular polymeric substances, in which bacteria embed themselves, and inducer molecules, responsible for bacterial communication or quorum-sensing, can modulate the expression of biofilm genes. Additionally, they can also influence bacterial aggregation [7].

**Figure 1:**
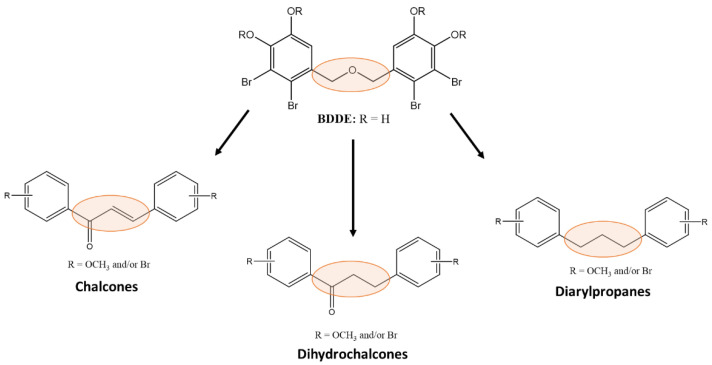
BDDE as models for new chalcone derivatives (chalcones, dihydrochalcones and diarylpropanes) with potential antimicrobial activity.

Chalcones are a class of natural products containing two benzene rings connected by a three-atom bridge. Depending on the nature and position of substituents, chalcones show a wide variety of biological activities, making the chalcone scaffold a “privileged structure” in Medicinal Chemistry [8]. This class of flavonoids has been reported for their wide array of biological activities, including antibacterial effects through the interference with several mechanisms [8,9]. Interestingly, it has already been described that chalcones, along with the structurally related flavones, behave as inhibitors of the EPs of the major facilitator superfamily (MFS), a group of EPs with particular relevance in Gram-positive bacteria. Moreover, previous structure–activity relationship (SAR) studies suggested that the presence of methoxy and bromine substitutions in chalcone derivatives could be beneficial for the antibacterial activity [9,10,11]. Nevertheless, the exploration of the antibacterial activity of brominated chalcones is scarce, with their potential not being fully explored as antimicrobial adjuvants to revert antibiotic resistance [12,13]. Interestingly, in addition to halogenated chalcones, other brominated natural products have been reported for their promising antimicrobial activity. In particular, bis(2,3-dibromo-4,5-dihydroxybenzyl) ether (BDDE), the symmetric bromophenol produced by red and brown macroalgae [14,15,16,17], structurally related to chalcones, also possessing two benzene rings connected by a three-atom bridge, has been found attractive for its antibacterial and antifungal activities (Figure 1) [18]. BDDE showed potent inhibitory activity against several American Type Culture Collection (ATCC) standard bacteria, as well as clinical strains of *S. aureus* and *Pseudomonas aeruginosa*, showing minimum inhibitory concentration (MIC) values lower than 70 µg/mL [19]. Moreover, BDDE showed broad-spectrum antifungal activity, especially on the necrotrophic fungus *Botrytis cinerea* [20]. These promising results prompted us to explore the potential antimicrobial activity of a small library of BDDE-related chalcones. Using BDDE as inspiration, a small library of brominated chalcones was planned and synthesized and evaluated for their antimicrobial activity and potential in reverting antimicrobial resistance in bacteria, through the inhibition of EPs and biofilm formation. Moreover, brominated dihydrochalcones, and diarylpropanes, were prepared, aiming to investigate the influence of the enone moiety in the three-atom bridge between the two phenyl groups in the chalcone scaffold (Figure 1).

## 2. Results and Discussion

### 2.1. Synthesis

**Scheme 1:**
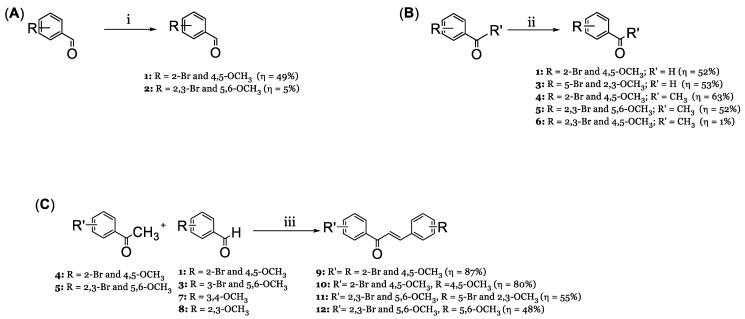
(**A**) Synthesis of brominated benzaldehydes **1** and **2**. (**B**) Synthesis of brominated benzaldehydes (**1** and **3**) and acetophenones (**4**-**6**). (**C**) Synthesis of brominated chalcones (**9**–**12**). (i) Br_2_, acetic acid, 70 °C, 1 h; (ii) NBS, C_2_H_7_NO_2_, CH_3_CN, r.t., 1.5–3 h; (iii) NaOH, MeOH, r.t., 2 h–1 week.

Nine brominated chalcone derivatives, including four chalcones, three dihydrochalcones, and two diarylpropanes, were synthetized. The synthesis of the brominated chalcones was successfully achieved by a base-catalyzed aldol condensation reaction between appropriately substituted acetophenones and benzaldehydes. In order to obtain chalcones with an oxygenation pattern in A and B rings similar to BDDE, commercially available benzaldehydes and acetophenones with two methoxy groups in the *ortho* position were selected to be used as building blocks. Firstly, 3,4-, and 2,3-dimethoxy-benzaldehydes and acetophenones were prepared by bromination (Scheme 1A,B). In this work, electrophilic aromatic bromination was firstly performed with Br_2_ for the synthesis of the brominated derivatives of 3,4-dimethoxy- and 2,3-dimethoxybenzaldehydes, in the presence of acetic acid (Scheme 1A). However, in both cases the reaction allowed the formation of a complex mixture of products which made it impossible to obtain the desired brominated derivatives with high yields. In fact, while the bromination of 3,4-dimethoxybenzaldehyde resulted in the formation of the monobrominated derivative 2-bromo-4,5-dimethoxybenzaldehyde (**1**) in a moderate yield (49%), the bromination of 2,3-dimethoxybenzaldehyde resulted in the formation of the dibrominated product 2,3-dibromo-5,6-dimethoxybenzaldehyde (**2**) in a very low yield (5%) (Scheme 1A). The bromination was then attempted with *N*-bromosuccinimide (NBS) as the bromination agent for the same benzaldehydes. In this reaction, the bromination of 3,4-dimethoxybenzaldehyde resulted in the formation of the same monobrominated derivative 2-bromo-4,5-dimethoxybenzaldehyde (**1**) with a slightly higher yield (52%) than when Br_2_ was used (49%). When these reaction conditions were applied for the bromination of 2,3-dimethoxybenzaldehyde, instead of obtaining 2,3-dibromo-5,6-dimethoxybenzaldehyde (**2**), 5-bromo-2,3-dimethoxybenzaldehyde (**3**) was obtained in a moderate yield (53%) (Scheme 1B). As the bromination with NBS allowed higher yields than Br_2_, it was decided to perform the bromination of 3,4-dimethoxy- and 2,3-dimethoxyacetophenones with NBS, furnishing **4** and **5** with yields of up to 50% (Scheme 1B). In addition to **4**, the bromination of 3,4-dimethoxyacetophenone also afforded the dibrominated derivative **6** as a by-product.

After that, the aldol condensation of acetophenones **4** and **5** with benzaldehydes **1**, **3**, 3,4-dimethoxybenzaldehyde (**7**) and 2,3-dimethoxybenzaldehyde (**8**), in the presence of basic reaction conditions allowed chalcones **9**, **10**, **11**, and **12** to be obtained, with yields between 48–87% (Scheme 1C).

**Scheme 2:**
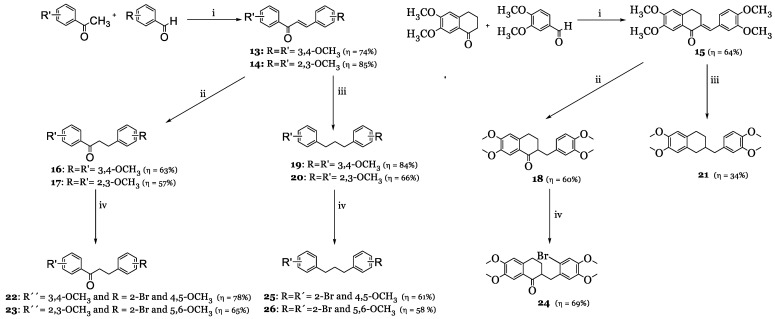
Synthesis of brominated dihydrochalcones (**22**–**24**) and diarylpropanes (**25** and **26**). (i) NaOH, MeOH, r.t., 2 h-1 week; (ii) Pd/C, H_2_, r.t., 2 h; (iii) Pd(OH)_2_/C, H_2_, r.t., 2 h; (iv) NBS, C_2_H_7_NO_2_, CH_3_CN, r.t., 2 h.

The approach used for the synthesis of brominated dihydrochalcones and diarylpropanes is represented in Scheme 2. In order to obtain brominated dihydrochalcones and diarylpropanes, chalcones **13**, **14**, and **15** were firstly synthesized and submitted to reduction reactions to the corresponding dihydrochalcones (**16**, **17**, and **18**) and diarylpropanes (**19**, **20**, and **21**), which were brominated afterwards. In order to obtain dihydrochalcones **16, 17**, and **18** and diarylpropanes **19**, **20**, and **21,** a catalytic hydrogenation using Pd/C and Pd(OH)_2_/C as catalysts was performed under H_2_ atmosphere at room temperature, respectively. These compounds were obtained with yields of between 34–84%. After purification, the dihydrochalcones and diarylpropanes were brominated with NBS, giving rise to dihydrochalcones **22**, **23**, **24** and diarylpropanes **25** and **26**, with yields of up to 60%.

The structure elucidation of compounds **1**, **3**, **4**, **6**, **9**, **10**, **13**–**16,** and **18**–**20** was established by infrared (IR) spectroscopy, and nuclear magnetic resonance (MNR), and full details are provided in the Supplementary Materials (Figures S1–S24). Data were in accordance with the previously reported [21,22,23,24,25,26,27,28]. The newly synthesized compounds **2**, **5**, **11**, **12**, **17**, and **21**–**26** were characterized by IR, NMR, and high-resolution mass spectrometry (HRMS) (Supplementary Materials, Figures S25–S56). ^13^C NMR assignments were determined by 2D heteronuclear single quantum correlation (HSQC) and heteronuclear multiple bond correlation (HMBC) experiments (Supplementary Materials, Figures S1–S56).

The coupling constants of the vinylic systems (J_Hα-Hβ_ = 15.5–16.4 Hz) confirm (*E*)-configuration of all the chalcones synthetized. 

### 2.2. Antibacterial Activity and Potentiation of Antimicrobials 

Chalcone derivatives **9**–**26** were firstly tested for their antimicrobial activity against five bacterial strains: the Gram-negative *Escherichia coli* ATCC 25922 and *P. aeruginosa* ATCC 27853, and the Gram-positive *S. aureus* ATCC 29213, *Enterococcus faecalis* ATCC 29212, and the methicillin- and ofloxacin-resistant clinical isolate *S. aureus* 272123. For the assay aimed at studying the capability of the compounds to restore the activity of the antibacterial drugs, two clinically relevant isolates were used: the extended-spectrum β-lactamase (ESBL)-producing *E. coli* SA/2 [29] and the vancomycin-resistant *E. faecalis* (VRE) B3/101 [29]. The antibiotics used were cefotaxime (CTX) and vancomycin (VAN), respectively.

The results of the antibacterial assay showed that these compounds presented a MIC value superior to the maximum concentration tested (MIC > 100 µM, data not shown). Concerning the potentiation of activity of the antimicrobials, fifteen chalcone derivatives (**9**, **13**–**23**, and **25**) were able to decrease the MIC of CTX in *E. coli* SA/2 and all the compounds reversed the resistance to vancomycin in *E. faecalis* B3/101, when tested at the previously determined sub-MIC concentration of 64 µg/mL (results not shown). In fact, compounds **9**, **13**–**23**, and **25** caused a two-fold decrease in the MIC of CTX for ESBL *E. coli* SA/2, and compounds **9**, **14**, and **24** caused a four-fold decrease of the MIC of VAN against *E. faecalis* B3/101, while the others caused a two-fold decrease (Table 1). It can be hypothesized that the compounds may interact with the broad-spectrum β-lactamases, in the case of *E. coli* SA/2. *E. faecalis* B3/101 has the capacity to modify the peptidoglycan synthesis pathway, thus it can also be a target for these compounds, but further studies are needed to elucidate the precise mechanisms.

### 2.3. Antifungal Activity 

The chalcone derivatives (**9**–**26**) were tested for their antifungal activity in *Candida albicans* ATCC 10231, *Aspergillus fumigatus* ATCC 204305, and *Trichophyton rubrum* FF5. The analysis of the results shows that only chalcones **13** and **14** were active at the maximal concentration tested (128 µg/mL). However, some selectivity was observed against *T. rubrum* FF5, with MIC of 128 µg/mL (**13**) and 16–32 µg/mL (**14**); MICs values were higher for *C. albicans* and *A. fumigatus.*

### 2.4. Efflux Pump Inhibition Assay

A screening of the compounds on the Gram-positive strain *S. aureus* 272123 was performed to study the capability of these chalcone derivatives to modulate the accumulation of ethidium bromide (EB), a known EP substrate, and therefore conclude about the potential of these compounds as EP inhibitors. *S. aureus* 272123 is a clinical strain and has been previously used to study the potential of xanthones and thioxanthones as efflux pump inhibitors [30]. In this strain, the expression of the *mepA* and *norA* genes were studied in the presence of antimicrobials of natural origin, and even though *norA* is a core gene of *S. aureus*, its expression levels were not changing [31,32,33]. 

Compounds **9**–**26** were tested at the concentration of 50 µM, as none of them had antibacterial activity in the strain used in this assay. The relative fluorescence index (RFI) was calculated based on the means of relative fluorescence units and are present in Table 1. Reserpine was used as the positive control, at the sub-MIC concentration of 25 µM.

From the analysis in Table 1, it can be noted that **12, 14**, and **17**–**24** can increase the fluorescence in comparison to the positive control, which can be attributed to the inhibition of the efflux of EB in the tested strains. The compounds can also emit fluorescence, which can pose a limitation for the assay. In this case, however, the analysis of the fluorescence curves over the time elapsed show that this is not a problem, as the compounds present approximately the same fluorescence as the controls in the beginning of the assay (Supplementary Materials, Figures S57–S74).

**Figure 2:**
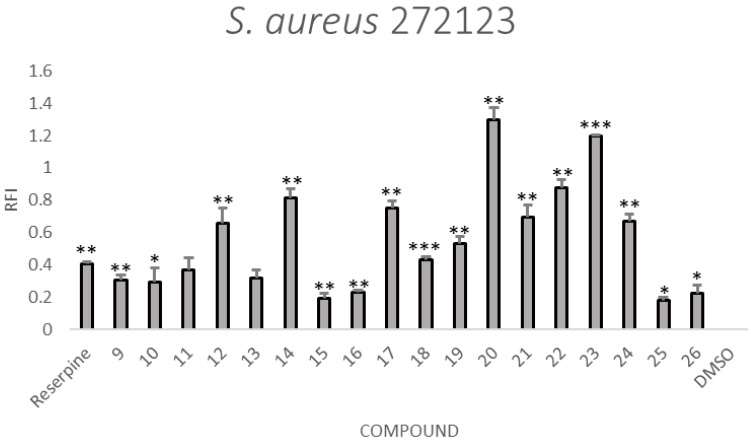
Relative fluorescence index (RFI) of the chalcone derivatives **9**–**26** in *S. aureus* 272123. Results are presented as mean ± SD. Statistical comparisons were performed using the *t*-test (* *p* < 0.05; ** *p* < 0.01; *** *p* < 0.001 vs. control (DMSO 1% *v/v*)).

From the analysis in Figure 2, it can be noted that compounds **12**, **14**, and **17**–**24** could inhibit the efflux of EB to a greater extent than reserpine, implying a possible underlying EP inhibition. The results obtained herein emphasize the usefulness of these compounds as EP inhibitors.

The results obtained herein also emphasize the potential of chalcones as EP inhibitors. In fact, a library of chalcone derivatives has previously been tested and displayed encouraging results against a bacterial strain whose resistance to norfloxacin was manifested through the overexpression of the NorA efflux pump [10].

**Figure 3:**
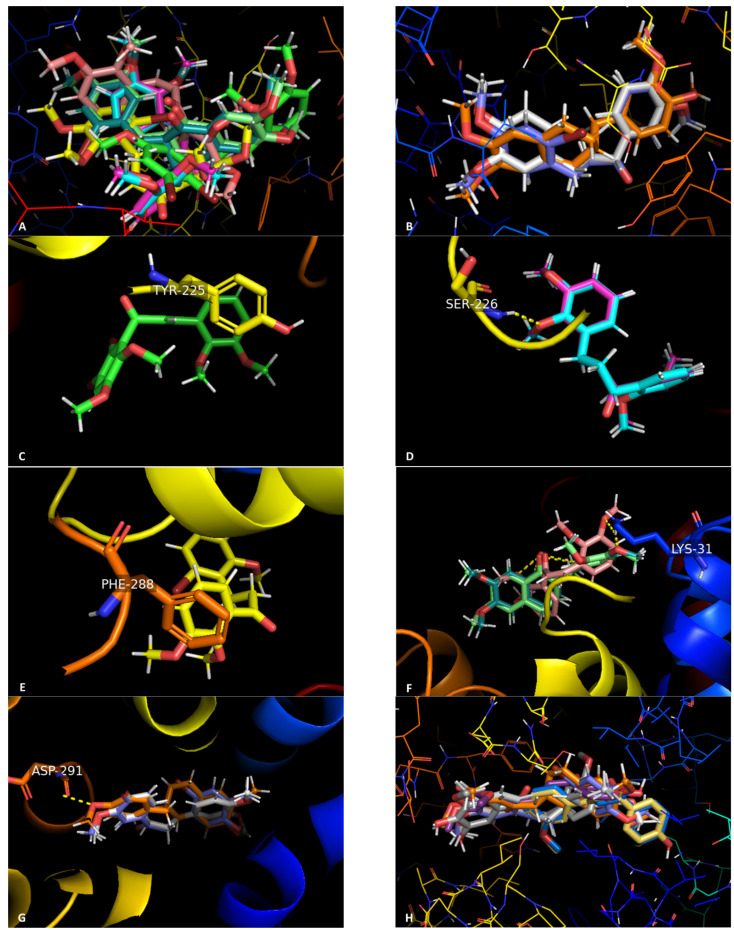
(**A**) Molecular visualization of compounds **12**, **14**, **17**, **18**, **20**, **22**, **23**, and **24** in the BCR of the NorA homology model; (**B**) Molecular visualization of compounds **19**, **21** and **25** in the BCR of the NorA homology model; (**C**) Interactions of compound **12** with the BCR; (**D**) Interactions of compounds **17** and **18** with the BCR; (**E**) Interactions of compound **23** with the BCR; (**F**) Interactions of compounds **14**, **18** and **24** with the BCR; (**G**) Interactions of compound **21** with the BCR; (**H**) Molecular visualization of compounds **a**–**d**, binding in the same site as compounds **19**, **21** and **25**.

To gain a better understanding of the molecular interactions of these compounds and the NorA EP, docking studies were performed (Table S1, Supplementary Materials). As this EP does not have a crystal structure deposited in the Protein Data Bank, a homology model was built. The compounds that displayed better results (−9.0 kcal/mol < docking score < −4.5 kcal/mol) than reserpine (docking score = 1.0 kcal/mol), were visualized using PyMol in the binding core region (BCR) of the homology model of NorA, as this was the site with which they were predicted to have better affinity (Figure 3). The chalcones described in [10] (compounds **a**–**d**, Figure S1, Supplementary Materials) were also used in this study, to check if the interactions were similar.

Through the analysis in Figure 3, it can be noted that all the compounds except **19**, **21**, and **25**, are predicted to bind in the same site (Figure 3A,B). An individual analysis of the compounds allowed for a better understanding of the residues they interact with, within the homology model of NorA. Compound **12** is predicted to establish a π-π interaction with Tyr-225 (Figure 3C). Compounds **17**, and **20** can establish a hydrogen bond between one of the methoxy groups and the amide in Ser-226 (Figure 3D). Compound **23** can interact with Phe-288 via a π-π stacking (Figure 3E). Compounds **14**, **18,** and **24** can interact with Lys-31, through a hydrogen bond between a methoxy and the amine in the residue (Figure 3F). Considering the three compounds that bind in a separate site, **19**, **21,** and **25**, it could be noted that only **21** had a relevant interaction with a residue of the homology model, as it was predicted that one of the oxygens in the methoxy group interacted with the amide group of Asp-291 (Figure 3G). Lastly, it could be observed that, in this model, compounds **a**–**d** are predicted to bind in the same site as compounds **19**, **21,** and **25** (Figure 3H). These are encouraging results, as these chalcones have shown potentiation of norfloxacin in a strain known to overexpress the NorA and MepA pumps. This is particularly more relevant concerning compound **c**, which was the most promising one [10]. However, in this study, we could not observe any predicted interaction that could explain the difference between this compound and the other ones that bind in the same site (results not shown).

### 2.5. Inhibition of Biofilm Formation

As previously mentioned, EPs are related to biofilm formation, as they can expel extracellular polymeric substances, quorum-sensing molecules, and modulate bacterial aggregation [7]. Therefore, the compounds that displayed good results in the real-time EB accumulation assay, **12**, **14**, **17**, **19**–**24**, were investigated for their effect on the biofilm formation of the sensitive and resistant *S. aureus* strains. The biofilm inhibition, presented as a percentage (%), was calculated based on the mean of absorbance units. Reserpine was used as the control in both strains, as it was the positive control used in the previous assay and has been described as both a biofilm inhibitor and an EP inhibitor for *S. aureus* strains [34]. The results obtained are shown in Table 1**.**

The results show that these chalcone derivatives inhibit the formation of biofilm to a greater extent in *S. aureus* 272123 than in the ATCC strain. In fact, only two compounds, **12** and **22**, were more effective than reserpine in *S. aureus* ATCC 25923. However, they could only display a slight inhibition, with 3% for **12**, and 13% for **22**. In the case of *S. aureus* 272123, all the compounds displayed an effect to different extents. Compound **14** is noteworthy, as it presented an inhibition of biofilm formation of 87% when compared to the control, even more than the inhibition promoted by reserpine. Compound **21** was also capable of a 71% inhibition, which is comparable to that of reserpine.

It can be speculated that these compounds are inhibiting the EPs responsible for the efflux of molecules involved in biofilm-related mechanisms. However, they can also inhibit other mechanisms related to biofilm formation, which are not linked to efflux inhibition.

**Figure 4:**
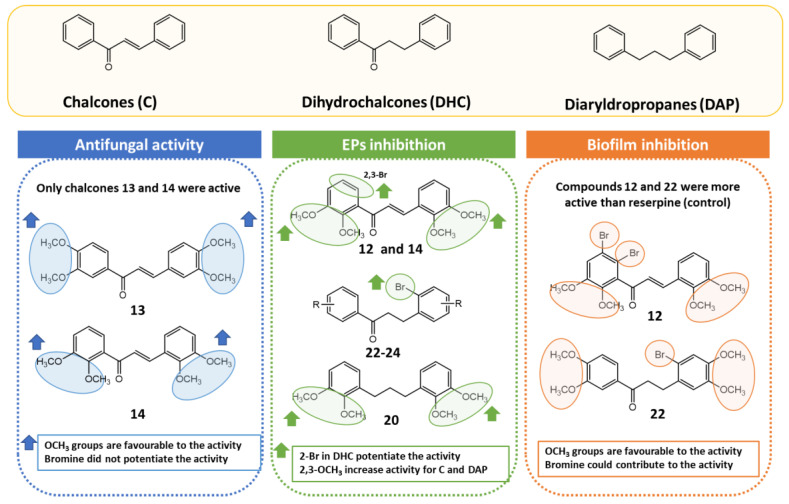
Main SAR conclusions.

Considering the overall biological activity results, some structure-activity relationship (SAR) conclusions can be proposed, as highlighted in Figure 4. For antifungal activity, SAR suggests that the presence of a non-hindered enone moiety between the two aromatic rings is beneficial for the activity, as chalcones **13** and **14** were the only ones to be effective. Regarding the ability to potentiate the activity of antimicrobials, it seems that the presence of two consecutive methoxy groups have the ability to increase the activity, being the 5,6-dimethoxy pattern that is the most favorable for the antifungal activity. Moreover, it seems that bromine substituents did not affect the activity.

For the real-time EB accumulation assay, in general, when comparing the results obtained for chalcones, dihydrochalcones, and diarylpropanes, it can be seen that dihydrochalcones and diarylpropanes showed more ability to accumulate EB than chalcones. Interestingly, all the dihydrochalcones showing promising EPs inhibitory effects possessed only one bromine atom in the position 2 of the aromatic B ring. In addition, the RFI value of the brominated derivatives **22**–**24** was superior to the RFI value of the structure-related non-brominated derivatives **16**–**18**, respectively, which evidenced the possible positive effect that the bromine substituent has in position 2 of the aromatic B ring in the chemical structure of the dihydrochalcones. Nevertheless, concerning diarylpropanes, non-brominated compounds were the only derivatives considered as promising, with **20** being the most potent compound, increasing the relative fluorescence index more than three-fold compared to reserpine. Overall results show that compounds bearing in their structure more than one bromine substituent did not show potential as EPs inhibitors, with the exception of chalcones **12** and **14**, that presented bromine substituents in positions 2 and 3 of the A ring.

Concerning the biofilm inhibitory effect, it seems that the presence of bromine substituents could be important for the activity, depending on the substitution pattern and the moiety of the linker between the two aromatic rings, as the brominated chalcone **12** and dihydrochalcone **22** were those showing the highest effect. 

### 2.6. Cytotoxicity Assay

The cytotoxicity of the derivatives with promising results in the EP inhibition assay **12**, **14**,and **17**–**24** was tested in mouse embryonic fibroblast cell lines (NIH/3T3). The IC_50_ of the tested compounds is presented in Table 2.

The dihydrochalcone and diarylpropane derivatives (**17**–**24**) do not present cytotoxicity for the tested cell line. On the other hand, the chalcone derivatives (**12** and **14**) that were tested presented cytotoxicity, suggesting that the presence of a α,β-unsaturated ketone could be responsible for this effect. The α,β-unsaturated ketone moiety could act as a Michael acceptor, which could lead to covalent binding to biomolecules, and be also considered as a potential Pan-Assay Interference Compounds (PAINs) structure [35]. Therefore, compounds **17**–**24**, could be regarded as hits for bacterial EP inhibition, as they are effective in the inhibition of EB efflux, but present other features that are important in this matter, such as the lack of antibacterial activity and cytotoxicity.

## 3. Materials and Methods

### 3.1. Synthesis

All the reagents and solvents were purchased from Sigma Aldrich and were used without further purification. Solvents were evaporated using a rotary evaporator under reduced pressure (Buchi Waterchath B-480). All reactions were monitored by thin-layer chromatography (TLC) carried out on precoated plates (silica gel, 60 F_254_ Merck) with 0.2 mm of thickness. The visualization of the chromatograms was under UV light at 254 nm and 365 nm. The purification of compounds was performed by crystallization, flash chromatography using Macherey-Nagel silica gel 60 (0.04–0.063 mm), and preparative thin-layer chromatography (TLC) using Macherey-Nagel silica gel 60 (GF_254_) plates. Melting points were obtained in a Köfler microscope and are uncorrected. ^1^H and ^13^C NMR spectra were taken in CDCl_3_ at room temperature, on Bruker Avance 300 instruments (300.13 MHz for ^1^H and 75.47 MHz for ^13^C). Chemical shifts are expressed in δ (ppm) values relative to tetramethylsilane (TMS) as an internal reference; ^13^C NMR assignments were made by 2D (HSQC and HMBC) NMR experiments (long-range ^13^C and ^1^H coupling constants were optimized to 7 Hz). The spectral treatment was executed using the MestReNova v6.0.2e5475 software. HRMS mass spectra were recorded at CEMUP–Centro de Materiais da Universidade do Porto, on an LTQ OrbitrapTM XL hybrid mass spectrometer (Thermo Fischer Scientific, Bremen, Germany) controlled by LTQ Tune Plus 2.5.5 and Xcalibur 2.1.0, in positive mode. The capillary voltage of the electrospray ionization source (ESI) was set to 3.1 kV. The capillary temperature was 275 °C. The sheath gas was at 6 (arbitrary unit, as provided by the software settings). The capillary voltage was 46V and the tube lens voltage was 120 V.

#### 3.1.1. Synthesis of Brominated Benzaldehydes **1**–**3** and Acetophenones **4**–**6**

**Bromination with Br_2_**

In a two-necked round bottom flask, 3,4-dimethoxybenzaldehyde (200 mg, 1.2 mmol), or 2,3-dimethoxybenzaldehyde (200 mg, 1.2 mmol), and acetic acid (6 mL) were placed. Then a solution of bromine in acetic acid (1:4) was added. The reaction mixture was stirred at reflux (70 °C) for 1 h. The reaction mixture was cooled at room temperature and a solution of 10% sodium thiosulfate was added until the orange color disappeared. A yellow powder was formed and the solid was filtered. The remaining mixture was extracted with chloroform (3 × 20 mL), and the organic phase was washed with brine solution (2 × 20 mL), dried over anhydrous sodium sulfate, filtered and then the solvent was evaporated under reduced pressure. The 2-bromo-4,5-dimethoxybenzaldehyde (**1**, 49%) was purified by flash column chromatography (SiO_2_, *n*-hexane: ethyl acetate 9:1). Compound **2** was purified and characterized as indicated below.

**2,3-dibromo-4,5-dimethoxybenzaldehyde (2):** Yield: 5% as a white solid; m.p. (methanol) = 97.8–100.1 °C; IR (KBr, ʋ (cm^-1^)): 3070 (C_sp2_-H); 2978, 2935, 2848 (C_sp3_-H); 1697 (C=O); 1570, 1557, 1469, 1442 (aromatic C=C); 1202 (C-O); 597 (C-Br); ^1^H NMR (300 MHz, CDCl_3_) δ (ppm): 10.21 (s, CHO), 7.34 (s, H-4), 3.91 (s, 5-OCH_3_), 3.89 (s, 6-OCH_3_); ^13^C NMR (300 MHz, CDCl_3_) δ (ppm): 190.3 (C=O), 153.0 (C-6), 150.3 (C-5), 121.4 (C-2), 120.5 (C-4), 114.7 (C-3), 62.5 (5-OCH_3_), 56.4 (6-OCH_3_). 

**Bromination with NBS**

In a two-necked round bottom flask, 3,4-dimethoxybenzaldehyde (500 mg, 3.0 mmol), 2,3-dimethoxybenzaldehyde (600 mg, 3.6 mmol), 3,4-dimethoxyacetophenone (500 mg, 2.8 mmol), or 2,3-dimethoxyacetophenone (500 mg, 2.8 mmol), 0.2 equivalents of ammonium acetate (55.7 mg, 722.1 µmol), and 20 mL of acetonitrile were placed. The round bottom flask was cooled to 0 °C, and after 15 min, two equivalents of NBS (1.3 g, 7.2 mmol) were added in one single portion. The mixture was allowed to stir at room temperature and protected from the light, for 2 h for the bromination of 3,4-dimethoxybenzaldehyde, 2,3-dimethoxybenzaldehyde, and 2,3-dimethoxyacetophenone, or 1.5 h for the bromination of 3,4-dimethoxyacetophenone. The solution was partitioned between water and ethyl acetate. The aqueous phase was extracted with ethyl acetate (2 × 25 mL) and the organic phase was washed with brine solution (2 × 25 mL), dried over sodium sulfate anhydrous, filtered and then the solvent was evaporated under reduced pressure. The 2-bromo-5,6-dimethoxybenzaldehyde (**3**, 53%) and 2-bromo-4,5-dimetoxyacetophenone (**4**, 63%) were purified by flash column chromatography (SiO_2_, *n*-hexane: ethyl acetate 9:1). The 2-bromo-4,5-dimethoxybenzaldehyde (**1**, 52%) and 2,3-dibromo-4,5-dimethoxyacetophenone (**6**, 1%) were purified by flash column chromatography (SiO_2_, *n*-hexane: ethyl acetate 95:5). Compounds **5** and **6** were purified and characterized as indicated below.

**2,3-dibromo-5,6-dimethoxyacetophenone (5):** purified by flash column chromatography (SiO_2_, *n*-hexane: ethyl acetate 95:5). Yield: 52% as a beige oil; IR (KBr, ʋ (cm^−1^)): 3081 (C_sp2_-H); 2974, 2940, 2837 (C_sp3_-H); 1713 (C=O); 1573, 1469, 1416 (aromatic C=C); 1246 (C-O); 649 (C-Br); ^1^H NMR (300 MHz, CDCl_3_) δ (ppm): 7.17 (*s*, H-4), 3.87 (*s*, 6-OCH_3_), 3.79 (*s*, 5-OCH_3_), 2.51 (*s*, H-1’); ^13^C NMR (300 MHz, CDCl_3_) δ (ppm): 200.8 (C=O), 152.6 (C-6), 144.8 (C-5), 139.7 (C-1), 120.2 (C-2), 117.4 (C-4), 109.6 (C-3), 62.1 (5-OCH_3_), 56.4 (6-OCH_3_), 31,3 (C-1′). ESI-HRMS (+) m/z: Anal. Cal. for (C_10_H_10_Br_2_O_3_Na_2_) (M+2Na)^+^: 382.89762 found: 381.29904.

#### 3.1.2. Synthesis of Chalcones **9**–**15**

In a round bottom flask the appropriately substituted ketone (50 mg–650 mg, 147.9 µmol–2.7 mmol) was dissolved in methanol and a solution of 40% sodium hydroxide was added until pH 13–14. Then, a solution of appropriately substituted benzaldehyde (49.2 mg–942.6 mg, 295.9 µmol–5.4 mmol) in methanol was added dropwise. The reaction mixture was stirred at room temperature for 2 h–1 week. At the end of the reaction, ice was added to quench the reaction, and a solution of 1M HCl was added until pH 5. For all synthesized chalcones, except **11** and **14**, the obtained solid was filtered and washed with cold distilled water. For compounds **11** and **14**, as the crude product was an oil, the crude product was extracted with chloroform (3 × 25 mL), and the organic phase was washed with brine solution (2 × 20 mL), dried over anhydrous sodium sulfate, filtered and then the solvent was evaporated under reduced pressure. Compounds **13**, and **15** were obtained without further purification. The 1,3-bis(2-bromo-4,5-dimethoxyphenyl)prop-2-en-1-one (**9**, 87%) was purified by flash column chromatography (SiO_2_, *n*-hexane/ethyl acetate 98:2), followed by TLC (SiO_2_, diethyl ether/petroleum ether 8:2). The (*E*)-1-(2-bromo-4,5-dimethoxyphenyl)-3-(3,4-dimethoxyphenyl)prop-2-en-1-one (**10**, 80%) was purified by TLC (SiO_2_, *n*-hexane/ethyl acetate 9:1). The (*E*)-1,3-bis(2,3-dimethoxyphenyl)prop-2-en-1-one (**14**, 85%) was purified by flash column chromatography (SiO_2_, *n*-hexane/ethyl acetate 9:1). Compounds **11** and **12** were purified and characterized as indicated below.

**(*E*)-3-(5-bromo-2,3-dimethoxyphenyl)-1-(2,3-dibromo-5,6-dimethoxyphenyl) prop-2-en-1-one (11):** purified by flash column chromatography (SiO_2_, *n*-hexane/ethyl acetate 95:5) Yield: 55% as an orange oil. m.p.(*n*-hexane/ethyl acetate 95:5) = 90.8–93.2 °C; IR (KBr, ʋ (cm^−1^)): 3079 (C_sp2_-H); 2970, 2936, 2829 (C_sp3_-H); 1655 (C=O); 1620, 1567, 1467, 1418 (aromatic C=C); 1376 (*trans* vinylic system); 1198 (C-O); 579 (C-Br); ^1^H NMR (300 MHz, CDCl_3_) δ (ppm): 7.51 (*d*, J = 16.4 Hz, H-β), 7.40 (*d*, J = 2.2 Hz, H-6), 7.25 (*s*, H-4′), 7.05 (*d*, J = 2.2 Hz, H-4), 6.91 (*d*, J = 16.4 Hz, H-α), 3.90 (*s*, 5′-OCH_3_), 3.85 (*s*, 3-OCH_3_), 3.77 (*s*, 6′-OCH_3_), 3.76 (*s*, 2-OCH_3_); ^13^C NMR (300 MHz, CDCl_3_) δ (ppm): 193.2 (C=O), 153.8 (C-3), 152.3 (C-5′), 147.9 (C-2), 145.9 (C-6′), 140.4 (C-β), 137.3 (C-1′), 129.9 (C-α), 128.6 (C-1), 122.1 (C-6), 120.1 (C-2′), 117.7 (C-4′), 117.6 (C-4), 116.9 (C-3′), 111.2 (C-5), 61.9 (6′-OCH_3_), 61.7 (2-OCH_3_), 56.3 (5′-OCH_3_), 56.2 (3-OCH_3_). ESI-HRMS (+) m/z: Anal. Cal. for (C_19_H_17_Br_3_O_5_) (M+H)^+^: 565.05200; found: 564.86898.

**(*E*)-1-(2,3-dibromo-5,6-dimethoxyphenyl)-3-(2,3-dimethoxyphenyl)prop-2-en-1-one (12):** purified by TLC (SiO_2_, *n*-hexane/ethyl acetate 9:1). Yield: 48% as a yellow-orange oil; IR (KBr, ʋ (cm^−1^)): 3078 (C_sp2_-H); 2937, 2836 (C_sp3_-H); 1732 (C=O); 1574, 1557, 1538, 1505, 1463, 1455 (aromatic C=C); 1373 (*trans* vinylic system); 1200 (C-O); 576 (C-Br); ^1^H NMR (300 MHz, CDCl_3_) δ (ppm): 7.60 (*d*, J =16.4 Hz, H-β), 7.25 (*s*, H-4′), 7.19 (*dd*, J = 8.0; 1.5 Hz, H-6), 7.07 (*t*, J = 8.0 Hz, H-5), 6.96 (*dd*, J = 8.0; 1.5 Hz, H-4), 6.95 (*d*, J = 16.4 Hz, H-α), 3.90 (*s*, 6′-OCH_3_), 3.86 (*s*, 3-OCH_3_), 3.78 (*s*, 5′-OCH_3_), 3.77 (*s*, 2-OCH_3_); ^13^C NMR (300 MHz, CDCl_3_) δ (ppm): 193.7 (C=O), 153.1 (C-3), 152.5 (C-6′), 148.8 (C-2), 145.9 (C-5′), 142.5 (C-β), 137.5 (C-1′), 128.6 (C-1), 127.9 (C-α), 124.3 (C-5), 120.0 (C-3′), 119.6 (C-6), 117.6 (C-4′), 114.7 (C-4), 61.8 (5′-OCH_3_), 61.6 (2-OCH_3_), 56.3 (6′-OCH_3_), 55.9 (3-OCH_3_). ESI-HRMS (+) m/z: Anal. Cal. for (C_19_H_18_Br_2_O_5_) (M+H)^+^: 486.95005: found: 486.95714.

#### 3.1.3. Synthesis of Dihydrochalcones **16**–**18**

A 10 mL round bottom flask with stir bar was charged with 0.2 equivalents of Pd/C 10% wt (13.0 mg, 121.8 µmol), chalcones **13** (200 mg, 609.1 µmol), **14** (200 mg, 609.1 µmol), or **15** (50 mg, 141.1 µmol), and 4 mL of toluene, with H_2_ atmosphere. The reaction mixture was left to stir with H_2_ atmosphere, at room temperature. The final product was filtered through silica gel flash, and recovered with ethyl acetate, to remove the Pd/C catalyst from the reaction mixture. The solvent was evaporated under reduced pressure. The 1,3-bis(3,4-dimethoxyphenyl)propan-1-one (**16**, 63%), and 2-(3,4-dimethoxybenzyl)-6,7-dimethoxy-3,4-dihydronaphthalene-1(*2H*)-one) (**18**, 60%) were purified by TLC (SiO_2_, *n*-hexane/ethyl acetate 6:4 and 7:3, respectively). Compound **17** was purified and characterized as indicated below.

**1,3-bis(2,3-dimethoxyphenyl)propan-1-one (17):** purified by TLC (SiO_2_, *n*-hexane/ethyl acetate 8:2). Yield: 57% as a colorless oil; IR (KBr, ʋ (cm^−1^)): 3076 (C_sp2_-H); 2937, 2835 (C_sp3_-H); 1682 (C=O); 1581, 1474, 1427 (aromatic C=C); 1172 (C-O); ^1^H NMR (300 MHz, CDCl_3_) δ (ppm): 7.15 (*dd*, J = 8.0; 2.1 Hz, H-6), 7.08 (*t*, J = 8.0 Hz, H-5′), 7.02 (*dd*, J = 8.0; 2.1 Hz, H-4′), 6.97 (*t*, J = 8.0 Hz, H-5), 6.83 (*dd*, J = 8.0; 2.1 Hz, H-4), 6.78 (*dd*, J = 8.0; 2.0; Hz H-6′), 3.89 (*s*, 3′-OCH_3_), 3.85 (*s*, 2′-OCH_3_, 3-OCH_3_), 3.83 (*s*, 2-OCH_3_), 3.27 (*t*, J = 7.7 Hz, H-β), 3.03 (*t*, J = 7.7 Hz, H-α) ); ^13^C NMR (300 MHz, CDCl_3_) δ (ppm): 202.6 (C=O), 153.0 (C-3), 152.8 (C-3′), 148.1 (C-2′), 147.3 (C-2), 135.2 (C-1′), 134.1 (C-1), 124.0 (C-5′), 123.9 (C-5), 122.0 (C-6′), 120.8 (C-6), 115.5 (C-4′), 110.4 (C-4), 61.2 (2′-OCH_3_), 60.6 (2-OCH_3_), 56.0 (3′-OCH_3_), 55.7 (3-OCH_3_), 44.0 (C-β), 24.8 (C-α). ESI-HRMS (+) m/z: Anal. Cal. for (C_19_H_22_O_5_) (M+H)^+^: 331.14672; found: 331.15394.

#### 3.1.4. Synthesis of Brominated Dihydrochalcones **22**–**24**

In a round bottom flask **16** (50 mg, 151.3 µmol), **17** (50 mg, 151.3 µmol), or **18** (50 mg, 140.3 µmol), 0.2 equivalents of ammonium acetate (2.3 mg, 30.3 µmol), and 5 mL of acetonitrile were placed. The round bottom flask was cooled to 0 °C, and after 15 min, two equivalents of NBS (53.9 mg, 302.7 µmol) were added in one single portion. The mixture was allowed to stir for 2 h, at room temperature. The solution was partitioned between distilled water and ethyl acetate. The aqueous phase was extracted with ethyl acetate (2 × 15 mL), and the organic phase was washed with brine solution (2 × 15 mL), dried over sodium sulfate anhydrous, filtered and then the solvent was evaporated under reduced pressure. Compounds **22**–**24** were purified and characterized as indicated below.

**3-(2-bromo-4,5-dimethoxyphenyl)-1-(3,4-dimethoxyphenyl)propan-1-one (22)**: purified by TLC (SiO_2_, *n*-hexane/ethyl acetate 6:4). Yield: 78% as a white-beige solid; m.p. (*n*-hexane/ethyl acetate 6:4) = 102.0–104.2 °C IR (KBr, ʋ (cm^−1^)): 3068, 3000 (C_sp2_-H); 2963, 2912 (C_sp3_-H); 1669 (C=O); 1595, 1584, 1507, 1464, 1457 (aromatic C=C); 1209 (C-O); 620 (C-Br); ^1^H NMR (300 MHz, CDCl_3_) δ (ppm): 7.56 (*dd*, J = 8.4; 2.0 Hz, H-6′), 7.53 (*d*, J = 2.0 Hz, H-2′), 7.01 (*s*, H-3), 6.88 (*d*, J = 8.4 Hz, H-5′), 6.82 (*s*, H-6), 3.94 (*s*, 4-OCH_3_), 3.93 (*s*, 4′-OCH_3_), 3.85 (*s*, 3′-OCH_3_), 3.84 (*s*, 5-OCH_3_), 3.23 (*t*, J = 7.5 Hz, H-β), 3.09 (*t*, J = 7.5 Hz, H-α); ^13^C NMR (300 MHz, CDCl_3_) δ (ppm): 197.9 (C=O), 153.3 (C-4′), 149.0 (C-4), 148.4 (C-5), 148.1 (C-3′), 132.6 (C-1′), 130.0 (C-1), 122.8 (C-6′), 115.5 (C-3), 113.9 (C-2), 113.0 (C-6), 110.1 (C-5′), 110.0 (C-2′), 56.2 (4-OCH_3_), 56.1 (2′-OCH_3_, 3′-OCH_3_), 56.0 (5-OCH_3_), 38.5 (C-β), 31.1 (C-α). ESI-HRMS (+) m/z: Anal. Cal. for (C_19_H_21_BrO_5_Na) (M+Na)^+^: 432.26577; found: 431.04876.

**3-(6-bromo-2,3-dimethoxyphenyl)-1-(2,3-dimethoxyphenyl)propan-1-one (23)**: purified by TLC (SiO_2_, *n*-hexane/ethyl acetate 6:4). Yield: 65% as a white-beige oil; IR (KBr, ʋ (cm^−1^)): 3080 (C_sp2_-H); 2938, 2837 (C_sp3_-H); 1683 (C=O); 1576, 1540, 1471, 1417 (aromatic C=C); 1174 (C-O); 612 (C-Br); ^1^H NMR (300 MHz, CDCl_3_) δ (ppm): 7.24 (*d*, J = 8.9 Hz, H-5), 7.21 (*dd*, J = 7.9; 1.8 Hz, H-6′), 7.09 (*t*, J = 7.9 Hz, H-5′), 7.03 (*dd*, J = 7.9; 1.8 Hz, H-4′), 6.68 (*d*, J = 8.9 Hz, H-4), 3.88 (*s*, 2′-OCH_3_), 3.87 (*s*, 2-OCH_3_), 3.84 (*s*, 3′-OCH_3_), 3.82 (*s*, 3-OCH_3_), 3.19 (*t*, J = 3.9 Hz, H-α, H-β); ^13^C NMR (300 MHz, CDCl_3_) δ (ppm): 202.2 (C=O), 153.1 (C-2), 152.3 (C-3), 148.3 (C-3′), 148.1 (C-2′), 135.1 (C-1′), 133.8 (C-1), 127.1 (C-5), 124.0 (C-5′), 120.8 (C-6′), 115.6 (C-4′), 115.4 (C-6), 111.5 (C-4), 61.6 (2′-OCH_3_), 61.0 (2-OCH_3_), 56.1 (3′-OCH_3_), 55.9 (3-OCH_3_), 42.5 (C-β), 25.1 (C-α). ESI-HRMS (+) m/z: Anal. Cal. for (C_19_H_23_BrO_5_) (M+H)^+^: 411.27600; found: 411.06331.

**2-(2-bromo-4,5-dimethoxybenzyl)-6,7-dimethoxy-3,4-dihydronaphthalen-1(2*H*)-one (24)**: purified by TLC (SiO_2_, n-hexane/ethyl acetate 6:4). Yield: 69% as a white-beige solid; m.p. (*n*-hexane/ethyl acetate 6:4) = 138.4–139.2 °C IR (KBr, ʋ (cm^−1^)): 3078 (C_sp2_-H); 2966, 2938, 2851 (C_sp3_-H); 1670 (C=O); 1577, 1559, 1540, 1508, 1465, 1457 (aromatic C=C); 1197 (C-O); 565 (C-Br); ^1^H NMR (300 MHz, CDCl_3_) δ (ppm): 7.54 (*s*, H-2′), 7.01 (*s*, H-3), 6.79 (*s*, H-6), 6.64 (*s*, H-5′), 3.93 (*s*, 4-OCH_3_), 3.92 (*s*, 5-OCH_3_), 3.86 (*s*, 3′-OCH_3_, 4′-OCH_3_), 3.55 (*m*, H-βa), 2.71 (*m*, H-βb), 2.89 (*m*, H-2″), 2.82 (*m*, H-α), 2.10 (*m*, H-1″a), 1.87 (*m*, H-1″b); ^13^C NMR (300 MHz, CDCl_3_) δ (ppm): 198.2 (C=O), 153.5 (C-4′), 148.3 (C-4), 148.0 (C-3′, C-5), 138.9 (C-1), 131.7 (C-1′), 125.6 (C-6′), 115.5 (C-2), 114.6 (C-6), 114.0 (C-3), 110.1 (C-2′), 108.7 (C-5′), 56.2 (4-OCH_3_), 56.1 (5-OCH_3_, 4′-OCH_3_), 56.0 (3′-OCH_3_), 47.9 (C-α), 35.7 (C-β), 28.6 (C-2″), 28.3 (C-1″). ESI-HRMS (+) m/z: Anal. Cal. for (C_21_H_24_BrO_5_) (M+H)^+^: 435.07289; found: 435.07980.

#### 3.1.5. Synthesis of Diarylpropanes **19**–**21**

To a solution of chalcone **13** (200 mg, 609.1 µmol), **14** (200 mg, 609.1 µmol), or **15** (50 mg, 141.1 µmol) in ethyl acetate (6 mL), and H_2_O (2 mL) was added 0.2 equivalents of 20% wt Pd(OH)_2_/C (17.1 mg, 121.8 µmol). The flask was left in H_2_ atmosphere for 2 h. After stirring at room temperature, the reaction mixture was filtered through silica gel and recovery with ethyl acetate, to remove the Pd(OH)_2_/C catalyst from the reaction mixture. The filtrate was evaporated under reduced pressure. The 1,3-bis(3,4-dimethoxyphenyl)propane (**19**, 84%) and 1,3-bis(2,3-dimethoxyphenyl)propane (**20**, 66%) were purified by TLC (SiO_2_, *n*-hexane/ethyl acetate 6:4 and 7:3, respectively). Compound **21** was purified and characterized as indicated below.

**2-(3,4-dimethoxybenzyl)-6,7-dimethoxy-1,2,3,4-tetrahydronaphthalene (21)**: purified by TLC (SiO_2_, *n*-hexane/ethyl acetate 6:4). Yield: 34% as a white solid; m.p. (*n*-hexane/ethyl acetate 6:4) = 78.1–79.0 °C; IR (KBr, ʋ (cm^−1^)): 3002 (C_sp2_-H); 2931, 2913, 2833 (C_sp3_-H); 1588, 1516, 1457, 1439, 1417 (aromatic C=C), 1197 (C-O). ^1^H NMR (300 MHz, CDCl_3_) δ (ppm): 6.82 (*d*, J = 8.3 Hz, H-5′), 6.75 (*d*, J = 1.8 Hz, H-2′), 6.74 (*dd*, J = 8.3; 1.8 Hz, H-6′), 6.57 (*s*, H-2″), 6.52 (*s*, H-5″), 3.88 (*s*, 3″-OCH_3_, 4″-OCH_3_), 3.83 (*s*, 3′-OCH_3_), 3.81 (*s*, 4′-OCH_3_), 2.74 (*m*, H-3a), 2.65 (*m*, H-3b), 2.69 (*m*, H-1a), 2.39 (*m*, H-1b), 2.57 (*m*, H-1″′), 2.02 (*m*, H-2), 1.94 (*m*, H-2″′a), 1.42 (*m*, H-2″′b); ^13^C NMR (300 MHz, CDCl_3_) δ (ppm): 148.7 (C-3′), 147.2 (C-3″), 147.0 (C-4′), 146.9 (C-4″), 133.4 (C-1′), 128.4 (C-6″), 128.2 (C-1″), 121.0 (C-6′), 112.3 (C-5″), 111.9 (C-2′), 111.6 (C-5′), 111.0 (C-2″), 55.9 (3′-OCH_3_, 3″-OCH_3_, 4″-OCH_3_), 55.8 (4′-OCH_3_), 42.5 (C-3), 36.5 (C-1), 35.5 (C-2), 29.4 (C-2″′), 28.7 (C-1″′). ESI-HRMS (+) m/z: Anal. Cal. for (C_21_H_26_O_4_Na) (M+Na)^+^: 365.17288; found: 365.17198.

#### 3.1.6. Synthesis of Brominated Diarylpropanes **25** and **26**

In a two-necked round bottom flask **19** (50 mg, 158.0 µmol), or **20** (50 mg, 158.0 µmol), 0.2 equivalents of ammonium acetate (2.4 mg, 31.6 µmol) and 5 mL of acetonitrile were placed. The round bottom flask was placed at 0 °C, and after 15 min, four equivalents of NBS (112.5 mg, 632.1 µmol) were added in one single portion. The mixture was allowed to stir for 4 h, at room temperature. The solution was partitioned between distilled water and ethyl acetate. The aqueous phase was extracted with ethyl acetate (2 × 15 mL), and the organic phase was washed with brine solution (2 × 15 mL), dried over sodium sulfate anhydrous, filtered and then the solvent was evaporated under reduced pressure. Compounds **25** and **26** were purified and characterized as indicated below. 

**1,3-bis(2-bromo-4,5-dimethoxyphenyl)propane (25):** purified by TLC (SiO_2_, *n*-hexane/ethyl acetate 6:4). Yield: 61% as a white-beige solid; m.p. (*n*-hexane/ethyl acetate 6:4) = 127.8–128.1 °C; IR (KBr, ʋ (cm^−1^)): 3000 (C_sp2_-H); 2946, 2932, 2855, 2839 (C_sp3_-H); 1577, 1510, 1454 (aromatic C=C); 1210 (C-O); 675 (C-Br); ^1^H NMR (300 MHz, CDCl_3_) δ (ppm): 7.00 (*s*, H-6′, H-6″), 6.72 (*s*, H-3′, H-3″), 3.86 (*s*, 5′-OCH_3_, 5″-OCH_3_), 3.85 (*s*, 4′-OCH_3_, 4″-OCH_3_), 2.73 (*t*, J = 7.8 Hz, H-1, H-3), 1.90 (*m*, H-2); ^13^C NMR (300 MHz, CDCl_3_) δ (ppm): 148.3 (C-5′, C-5″), 147.8 (C-4′, C-4″), 133.4 (C-1′, C-1″), 115.5 (C-6′, C-6″), 114.1 (C-2′, C-2″), 112.9 (C-3′, C-3″), 56.2 (4′-OCH_3_, 4″-OCH_3_), 56.1 (5′-OCH_3_, 5″-OCH_3_), 35.5 (C-1, C-3), 30.5 (C-2). ESI-HRMS (+) m/z: Anal. Cal. for (C_19_H_23_Br_2_O_4_) (M+H)^+^: 474.98644; found: 474.99484.

**1,3-bis(6-bromo-2,3-dimethoxyphenyl)propane (26):** purified by TLC (SiO_2_, *n*-hexane/ethyl acetate 6:4). Yield: 58% as a white-beige solid; m.p. (n-hexane/ethyl acetate 6:4) = 162.7–163.4 °C; IR (KBr, ʋ (cm^−1^)): 2961, 2936, 2860, 2836 (C_sp3_-H); 1574,1472, 1456, 1435 (aromatic C=C), 1173 (C-O); 683 (C-Br); ^1^H NMR (300 MHz, CDCl_3_) δ (ppm): 7.21 (*d*, J = 8.8 Hz, H-5′, H-5″), 6.64 (*d*, J = 8.8 Hz, H-4′, H-4″), 3.83 (*s*, 2′-OCH_3_, 2″-OCH_3_), 3.82 (*s*, 3′-OCH_3_, 3″-OCH_3_), 2.88 (*t*, J = 8.1 Hz, H-1, H-3), 1.80 (*m*, H-2); ^13^C NMR (300 MHz, CDCl_3_) δ (ppm): 152.2 (C-2′, C-2″), 148.0 (C-3′, C-3″), 136.2 (C-1′, C-1″), 127.6 (C-5′, C-5″), 115.6 (C-6′, C-6″), 111.1 (C-4′, C-4″), 61.1 (2′-OCH_3_, 2″-OCH_3_), 55.8 (3′-OCH_3_, 3″-OCH_3_), 30.3 (C-1, C-3), 29.4 (C-2). ESI-HRMS (+) m/z: Anal. Cal. for (C_19_H_23_Br_2_O_4_) (M+H)^+^: 474.98644; found: 474.99543.

### 3.2. Biological Activity

#### 3.2.1. Culture Media and Chemicals

The culture media used in the experiments were the following: cation-adjusted Mueller-Hinton broth (MHB II; Sigma-Aldrich, St- Louis, MO, USA and Biokar Diagnostics, Allone, Beauvais, France), Tryptic Soy broth (TSB; Scharlau Chemie S. A., Barcelona, Spain), and Trypto-Casein Soy agar (TSA; Biokar Diagnostics) were purchased. Sabouraud Dextrose Agar (SDA) was purchased from Bio-Mérieux (Marcy L’Etoile, France), RPMI-1640 broth medium from Biochrom AG (Berlin, Germany), which was buffered with 3-(*N*-morpholino) propanesulfonic acid (MOPS), purchased from Sigma-Aldrich, to pH 7.0.

Dimethyl sulfoxide (DMSO), 3-(4,5-dimethylthiazol-2-yl)-2,5-diphenyltetrazolium bromide (MTT), sodium dodecyl sulfate (SDS), phosphate-buffered saline (PBS; pH 7.4), ethidium bromide (EB), reserpine, and crystal violet (CV) were purchased from Sigma-Aldrich Chemie GmbH (Steinheim, Germany). Doxorubicin 2 mg/mL was purchased from Teva Pharmaceuticals, Budapest, Hungary. The antibiotics cefotaxime (CTX) was purchased from Duchefa Biochemie, Haarlem, The Netherlands, and vancomycin (VAN) from Oxoid, Basingstoke, England.

#### 3.2.2. Microorganisms

In this study, four ATCC strains were used for the antibacterial activity assay: the Gram-negative *E. coli* ATCC 25922 and *P. aeruginosa* ATCC 27853, and the Gram-positive *S. aureus* ATCC 25923 and *E. faecalis* ATCC 29212. Regarding the antimicrobial potentiation assay, environmental isolates vancomycin-resistant enterococci (VRE) *E. faecalis* B3/101 [29], and clinical isolates of the extended-spectrum β-lactamase (ESBL)-producing *E. coli* SA/2 were used. For the real-time EB accumulation assay, the methicillin- and ofloxacin-resistant *S. aureus* 272123 clinical isolate was investigated.

For the antifungal assays, the yeast *C. albicans* ATCC 10231, the filamentous fungi *A. fumigatus* ATCC 204305, and the clinical isolated dermatophyte *T. rubrum* FF5 were used. Cultures were obtained in SDA.

#### 3.2.3. Antibacterial Assay and Potentiation of Antibiotics

According to the Clinical and Laboratory Standard Institute (CLSI) guidelines, the MIC for each compound was obtained via the broth microdilution method, and the assays were performed in a 96-well plate [36]. For *S. aureus* 272123, the compounds were tested from 100 µM to 0.195 µM, diluted in MHB II from a previously prepared stock solution of 10 mM in DMSO; for the other strains used herein, the concentrations tested ranged from 64 µg/mL to 4 µg/mL, obtained from a previously prepared stock solution of 10 mg/mL in DMSO, which was used in sub-inhibitory concentrations (1% *v/v*), which was also used as the control. The MIC was determined visually, through the observation of the lack of bacterial growth in comparison to a positive control (bacterial strain in culture media).

The combined effect of the compounds and clinically relevant antimicrobial drugs against resistant strains was evaluated by determining the antibiotic MIC in the presence of each compound. Briefly, the antibiotics cefotaxime (CTX) and vancomycin (VAN) for *E. coli* SA/2 and *E. faecalis* B3/101, respectively, were serially diluted, and their MIC was determined in the presence of the fixed concentration of 64 µg/mL of the compound, as this was the highest concentration of compound that was observed to not affect bacterial growth when used alone. Antibiotic MICs were determined as described above, and the results were presented as the number of times the MIC was decreased in comparison to the antibiotic alone.

#### 3.2.4. Antifungal Assay

The compounds were assayed for their antifungal activity through the broth microdilution assay, based on the CLSI protocols M27A-3 for yeasts [37] and M38-A2 [38] for filamentous fungi. RPMI-1640 was used as broth culture media. The compounds were tested at concentrations between 128 µg/mL and 8 µg/mL. The MIC was considered the lowest concentration that was able to totally inhibit fungal growth in comparison to the positive control (fungal strain in culture medium). A DMSO control was included, similarly to what was described in Section 3.2.3.

#### 3.2.5. Efflux Pump Inhibition Assays

The compounds were evaluated for their ability to inhibit EPs of *S. aureus* 272123, through the real-time fluorimetry, monitoring of the intracellular accumulation of EB, an EP substrate. This was determined by the automated method using a CLARIOstar Plus plate reader (BMG Labtech, Ortenberg, Germany). Reserpine was applied at the sub-MIC concentration of 25 µM as the positive control, and the solvent DMSO was applied at 1% *v/v*.

The bacteria were incubated in TSB at 37 °C until an optical density (OD) of 0.6 at 600 nm was reached. The culture was centrifuged at 13,000× *g* for 3 min, and the pellet washed and resuspended with phosphate buffered saline (PBS, pH 7.4). The suspension was centrifuged again in the same conditions and resuspended in PBS.

The compounds were applied at 50 µM, as none of them displayed antibacterial activity for *S. aureus* 272123. Then, 50 µL of this solution were transferred into a 96-well black microtiter plate (Greiner Bio-One Hungary Kft, Hungary), and 50 µL of bacterial suspension (OD_600_ 0.4–0.6) were added to each well.

The plates were placed into the CLARIOstar plate reader, and the fluorescence was monitored at excitation and emission wavelengths of 530 nm and 600 nm, respectively, every minute for one hour in a real-time basis. From the real-time data, the activity of the compounds, namely the relative fluorescence index (RFI) of the last time point (minute 60) of the EB accumulation assay, was calculated according to the following formula:

(1) $$\mathrm{RFI}=\frac{{\mathrm{RF}}_{\mathrm{treated}}-{\mathrm{RF}}_{\mathrm{untreated}}}{{\mathrm{RF}}_{\mathrm{untreated}}}$$

where RF_treated_ is the relative fluorescence (RF) at the last time point of the EB accumulation curve in the presence of the compound, and RF_untreated_ is the RF at the last time point of the EB accumulation curve of the untreated control, having only the solvent (DMSO) control [39]. The accumulation curves were designed using Microsoft Excel^®^. The samples were tested in triplicate, and the RFI presented comes from the average of these three values. The accumulation curves present the mean of the RF over the cycles performed. The standard deviation (SD) was calculated automatically and included in the RF curves. The *p* values were obtained with a *t*-test.

#### 3.2.6. Docking Studies

Docking studies were performed in a homology model of the EP NorA, since this is the most relevant EP in Gram-positive bacteria [40]. However, NorA does not yet have a crystal structure deposited in the Protein Data Bank, which led to the preparation of a homology model. Using the Swiss Model server [41], the model was generated and the sequence was deposited in UniProt (Q5HHX4) [42], using the EmrD pump from *E. coli* (PDB: 2GFP) as the homolog, as described in [43]. Reserpine, a known NorA inhibitor [44] was used as control, and compounds **9**–**26** were drawn with ChemDraw (PerkinElmer Informatics) and minimized using ArgusLab. Docking was carried out using AutoDock Vina (Scripps, CA, USA) [45], The top nine poses were collected for each molecule and the most favorable binding conformation was attributed to the lowest docking score value. For molecular visualization, PyMol (Schrödinger, NY, USA) was used [46].

#### 3.2.7. Inhibition of Biofilm Formation

The most promising EP inhibitors, chalcone derivatives **12**–**14**, dihydrochalcone derivatives **17**, **22**–**24**, and diarylpropane derivatives **19**–**21**, were tested for their ability to decrease the formation of biofilm in the Gram-positive pair *S. aureus* ATCC 25923 and *S. aureus* MRSA 272123. The produced biofilm was detected using the dye crystal violet (CV; 0.1% *v/v*). The inoculum was prepared from an overnight incubation of the bacterial strains, which was diluted to an OD_600_ of 0.1. This suspension was added to 96-well microtiter plates and the compounds were added at the sub-MIC concentration of 100 µM, to a final volume of 200 µL. The EP and biofilm formation inhibitor, reserpine, was used as the positive control [34]. 

The plates were incubated for 48 h with gentle stirring (100 rpm) at 30 °C. After this period, the medium was discarded, and the unattached cells were removed through a washing step, using tap water. In the next step, the biomass attached to the wells was stained with 200 µL of a 0.1% (*v/v*) CV solution, which was incubated for 15 min at room temperature. Then, the CV solution was discarded from the wells, and after another washing step with tap water, 200 µL of a solution of 70% ethanol was added to the wells.

The OD_600_ was measured using a Multiscan EX ELISA plate reader (Thermo Labsystems, Cheshire, WA, USA). The anti-biofilm effect of the compounds was expressed in the percentage (%) of decrease of biofilm formation in comparison to a positive control (bacteria and media without compound) [47].

#### 3.2.8. Cytotoxicity Assay

The compound concentrations used were the same as the antibacterial activity assay and prepared from the same 10 mM stock solution in DMSO, diluted freshly on the day of the experiment in cell culture media.

The cell line used was mouse fibroblasts (NIH/3T3, ATCC CRL-1658TM), cultivated in DMEM (Gibco 52100-039), and supplemented with 10% heat-inactivated fetal bovine serum (Biowest, VWR International Kft, Debrecen, Hungary), 2 mM of L-glutamine, 1 mM Na pyruvate, 100 U/L, and 10 mg/L of penicillin/streptomycin mixture (Sigma-Aldrich Chemie GmbH, Steinheim, Germany), respectively, and 0.1% nystatin (8.3 g/L in ethylene glycol). Using a combination of 0.25% Trypsin-Versene (EDTA) solution for 5 min at 37 °C, the adherent cells were detached. Prior to each cytotoxicity assay, the cells were seeded in untreated 96-well flat-bottom microtiter plates, which was followed by a four-hour incubation period in a humidified atmosphere (5% CO_2_, 95% air) at 37 °C [48].

The MTT assay was used to determine the cytotoxicity of the compounds against NIH/3T3 cells. Prior to the assay, the cells were seeded for four hours using 1 × 10^4^ cells/well. The compounds were added by two-fold serial dilutions to the cells distributed into the 96-well flat bottom microtiter plates, starting with 100 μM. Then, after the plates had been incubated for 24 h, a solution of MTT in PBS was added to each well, followed by a four-hour incubation period. After this, 100 μL of SDS (10% in a 0.01 M HCl solution) wase added to each well and incubated overnight at 37 °C. As a positive control, the drug doxorubicin was used. Cell growth was determined in quadruplicate by measuring OD at λ = 540 nm (reference 630 nm) in a Multiscan EX ELISA reader (Thermo Labsystems, Cheshire, WA, USA). The percentage of inhibition of cell growth was determined according to the Equation (2).

(2) $$100-\left(\frac{{\mathrm{OD}}_{\mathrm{sample}}-{\mathrm{OD}}_{\mathrm{medium}\;\mathrm{control}}}{{\mathrm{OD}}_{\mathrm{cell}\;\mathrm{control}}-{\mathrm{OD}}_{\mathrm{medium}\;\mathrm{control}}}\right)\times 100$$

The results were expressed as the mean ± standard deviation (SD), and the IC_50_ values were obtained by best fitting the dose-dependent inhibition curves in GraphPad Prism 5.03 for Windows software.

## 4. Conclusions 

Infections due to antibiotic-resistant bacteria constitute an alarming concern, which can lead to a global health problem. As resistance development limits the useful lifespan of available antimicrobial drugs, the need for the discovery of new compounds capable of fighting bacterial- and fungal-derived infections is urgent. Flavonoids, especially chalcones, have been reported for their vast number of biological activities, which includes their potential to act as antibacterial agents. This assessment can be complemented by an alternative goal of simply suppressing the resistance mechanism of multi-drug resistant pathogens, namely by inhibiting the EPs. Along with chalcones, the macroalgae-derived metabolite BDDE has been already reported for its interesting antibacterial and antifungal activities. Inspired by these results, a small library of brominated chalcones, dihydrochalcones, and diarylpropanes was designed, synthesized, and evaluated for their antifungal and antibacterial activities. For antifungal activity, two chalcones, **13** and **14**, were active against *T. rubrum* FF5, with MIC values of 128 and 16–32 µg/mL, respectively. Furthermore, all the compounds were able to reverse the resistance to vancomycin in the resistant *E. faecalis* B3/101 strain, with **9**, **14,** and **24** being the most promising, able to decrease the MIC of vancomycin by four-fold, and the remaining induced a two-fold decrease of the MIC, for the same strain. Among these compounds, **14** and **21** showed capabilities of inhibiting the formation of biofilm in sensitive and resistant *S. aureus* (87% and 71% of inhibition, respectively). To conclude, and considering all the results obtained, compounds **17**–**24** could be selected as hit compounds, as they can exert activity against one or more bacterial resistance mechanisms. Since our study focuses on the inhibition of EPs, further studies are warranted and planned, in order to determine if the efflux inhibition demonstrated by these compounds is due to the inhibition of EPs and, if so, which pump is being inhibited, as the assay used herein is not specific for a certain EP. It can, however, pinpoint the potential of this class of compounds, and strengthen their position as potential antimicrobial adjuvants.

## Figures and Tables

**Table 1:** Effects of compounds **9**–**26** in the antimicrobial activity, potentiation of antimicrobials, EP inhibition, and inhibition of biofilm formation assays.

Compounds	Antibacterial Activity and Potentiation of Antimicrobials (CTX: Cefotaxime; VAN: Vancomycin)		EP Inhibition Assay	Inhibition of Biofilm Formation (%)	
	*E. coli* SA/2 MIC CTX = 562 µM	*E. faecalis* B3/101 MIC VAN = 707 µM	Relative Fluorescence Index (RFI) ± SD		
	MIC Reduction	MIC Reduction	*S. aureus* 272123	*S. aureus* ATCC 25923	*S. aureus* 272123
**9**	2-fold	4-fold	0.31 ± 0.03	-	-
**10**	No effect	2-fold	0.29 ± 0.09	-	-
**11**	No effect	2-fold	0.37 ± 0.08	-	-
**12**	2-fold	2-fold	0.66 ± 0.09	3.05 ± 1.41	7.76 ± 2.14
**13**	2-fold	2-fold	0.32 ± 0.04	-	-
**14**	2-fold	4-fold	0.81 ± 0.06	0	87.28 ± 3.84
**15**	2-fold	2-fold	0.37 ± 0.03	-	-
**16**	2-fold	2-fold	0.23 ± 0.01	-	-
**17**	2-fold	2-fold	0.75 ± 0.04	0	6.89 ± 2.41
**18**	2-fold	2-fold	0.43 ± 0.02	0	8.15 ± 0.64
**19**	2-fold	2-fold	0.53 ± 0.04	0	49.59 ± 0.39
**20**	2-fold	2-fold	1.30 ± 0.08	0	23.80 ± 0.13
**21**	2-fold	2-fold	0.70 ± 0.07	0	71.33 ± 1.09
**22**	2-fold	2-fold	0.88 ± 0.05	13.28 ± 6.67	7.95 ± 0.65
**23**	2-fold	2-fold	1.20 ± 0.01	0	7.08 ± 2.72
**24**	No effect	4-fold	0.67 ± 0.04	0	10.65 ± 0.76
**25**	2-fold	2-fold	0.18 ± 0.02	-	-
**26**	No effect	2-fold	0.22 ± 0.05	-	-
Reserpine (control)	-	-	0.41 ± 0.01	2.49 ± 1.38	77.62 ± 4.08

**Table 2:** Cytotoxicity (IC_50_) of the tested compounds.

Name	IC_50_ (µM) ± SD
Doxorubicin	12.05 ± 0.81
**12**	28.31 ± 0.25
**14**	30.16 ± 1.04
**17**	>100
**18**	>100
**22**	>100
**23**	>100
**24**	>100
**19**	>100
**20**	>100
**21**	>100

## Supplementary Materials

The following supporting information can be downloaded at: https://www.mdpi.com/article/10.3390/md20050315/s1, Figures S1–S24: NMR spectra and characterization of the compounds **1**, **3**, **4**, **6**, **9**, **10**, **13**–**16**, **18**, and **20**; Figures S25–S56: NMR and HRMS spectra of compounds **2**, **5**, **11**, **12**, **17**, and **21**–**26**; Figures S57–S74: RFI of compounds **9**–**26**, reserpine (positive control) and DMSO (negative control); Figure S75: Chalcones described in [10], used in the docking studies; Table S1: Docking results for the compounds in NorA.
