# Supplementary material for: BDDE-Inspired Chalcone Derivatives to Fight Bacterial and Fungal Infections
Source: Mar Drugs. 2022 May 8;20(5):315. doi: 10.3390/md20050315 (PMC9147945; doi:10.3390/md20050315)
Supplement: Supplementary file 1 [file marinedrugs-20-00315-s001.zip › marinedrugs-1714143- supplementary.pdf]

marine drugs

Article

BDDE-inspired chalcone derivatives to fight bacterial and fungal infectious

Ana Jesus [1,2,†], Fernando Durães [1,2,†], Nikoletta Szemerédi [3], Joana Freitas-Silva [2,4], Paulo Martins da Costa [2,4], Eugénia Pinto [2,5], Madalena Pinto [1,2], Gabriella Spengler [3], Emília Sousa [1,2,*], Honorina Cidade [1,2,*]

1 Laboratory of Organic and Pharmaceutical Chemistry, Department of Chemical Sciences, Faculty of Pharmacy, University of Porto, Rua Jorge Viterbo Ferreira, 228, 4050-313 Porto, Portugal; anaaimjesus@gmail.com (A.J.), fduraes5@gmail.com (F.D.); madalena@ff.up.pt (M.P.)
2 CIIMAR—Interdisciplinary Centre of Marine and Environmental Research, University of Porto, Avenida General Norton de Matos, 4450-208 Matosinhos, Portugal; joanafreitasdasilva@gmail.com (J.F.-S.); pmcosta@icbas.up.pt (P.M.d.C.) epinto@ff.up.pt (E.P.)
3 Department of Medical Microbiology, Albert Szent-Györgyi Health Center and Albert Szent-Györgyi Medical School, University of Szeged, Semmelweis utca 6, 6725 Szeged, Hungary; szemeredi.nikoletta@med.u-szeged.hu (N.S.), spengler.gabriella@med.u-szeged.hu (G.S.)
4 ICBAS – Institute of Biomedical Sciences Abel Salazar, University of Porto, Rua de Jorge Viterbo Ferreira 228, 4050-313 Porto, Portugal
5 Laboratory of Microbiology, Department of Biological Sciences, Faculty of Pharmacy, University of Porto, Rua de Jorge Viterbo Ferreira 228, 4050-313 Porto, Portugal;
† These authors contributed equally to this work and share first authorship.
* Corresponding authors: esousa@ff.up.pt (E.S); hcidade@ff.up.pt (H.C.).

Supplementary material

Table of contents:

NMR spectra and characterization of the compounds 1, 3, 4, 6, 9, 10, 13-16, 18, and 20. 1

Figure S1. ^{1}H NMR and ^{13}C NMR spectra of compound **1**. 1

Figure S2. HSQC and HMBC spectra of compound **1**. 2

Figure S3. ^{1}H NMR and ^{13}C NMR spectra of compound **3**. 3

Figure S4. HSQC and HMBC spectra of compound **3**. 4

Figure S5. ^{1}H NMR and ^{13}C NMR spectra of compound **4**. 5

Figure S6. HSQC and HMBC spectra of compound **4**. 6

Figure S7. ^{1}H NMR and ^{13}C NMR spectra of compound **6**. 7

Figure S8. HSQC and HMBC spectra of compound **6**. 8

Figure S9. ^{1}H NMR and ^{13}C NMR spectra of compound **9**. 9

Figure S10. HSQC and HMBC spectra of compound **9**. 10

Figure S11. ^{1}H NMR and ^{13}C NMR spectra of compound **10**. 11

Figure S12. HSQC and HMBC spectra of compound **10**. 12

Figure S13. ^{1}H NMR and ^{13}C NMR spectra of compound **13**. 13

Figure S14. HSQC and HMBC spectra of compound **13**. 14

Figure S15. ^{1}H NMR and ^{13}C NMR spectra of compound **14.** 15

Figure S16. ^{1}H NMR and ^{13}C NMR spectra of compound **14.** 16

Figure S17. ^{1}H NMR and ^{13}C NMR spectra of compound **15.** 17

Figure S18. HSQC and HMBC spectra of compound **15.** 18

Figure S19. ^{1}H NMR and ^{13}C NMR spectra of compound **16.** 19

Figure S20. HSQC and HMBC spectra of compound **16.** 20

Figure S21. ^{1}H NMR and ^{13}C NMR spectra of compound **18.** 21

Figure S22. ^{1}H NMR and ^{13}C NMR spectra of compound **18.** 22

Figure S23. ^{1}H NMR and ^{13}C NMR spectra of compound **20.** 23

Figure S24. ^{1}H NMR and ^{13}C NMR spectra of compound **20.** 24

NMR and HRMS spectra of compounds 2, 5, 11, 12, 17, and 21-26. 25

Figure S25. ^{1}H NMR and ^{13}C NMR spectra of compound **2.** 25

Figure S26. HSQC and HMBC spectra of compound **2.** 26

Figure S27. ^{1}H NMR and ^{13}C NMR spectra of compound **5.** 27

Figure S28. HSQC and HMBC spectra of compound **5.** 28

Figure S29. HRMS spectrum of compound **5.** 29

Figure S30. ^{1}H NMR and ^{13}C NMR spectra of compound **11.** 30

Figure S31. HSQC and HMBC spectra of compound **11.** 31

Figure S32. HRMS spectrum of compound **11.** 32

Figure S33. ^{1}H NMR and ^{13}C NMR spectra of compound **12.** 33

Figure S34. HSQC and HMBC spectra of compound **12.** 34

Figure S35. HRMS spectrum of compound **12.** 35

Figure S36. ^{1}H NMR and ^{13}C NMR spectra of compound **17.** 36

Figure S37. HSQC and HMBC spectra of compound **17.** 37

Figure S38. HRMS spectrum of compound **17.** 38

Figure S39. ^{1}H NMR and ^{13}C NMR spectra of compound **21.** 39

Figure S40. HSQC and HMBC spectra of compound **21.** 40

Figure S41. HRMS spectrum of compound **21.** 41

Figure S42. ^{1}H NMR and ^{13}C NMR spectra of compound **22.** 42

Figure S43. HSQC and HMBC spectra of compound **22.** 43

Figure S44. HRMS spectrum of compound **22.** 44

Figure S45. ^{1}H NMR and ^{13}C NMR spectra of compound **23.** 45

Figure S46. HSQC and HMBC spectra of compound **23**. 46

Figure S47. HRMS spectrum of compound **23**. 47

Figure S48. ^{1}H NMR and ^{13}C NMR spectra of compound **24**. 48

Figure S49. HSQC and HMBC spectra of compound **24**. 49

Figure S50. HRMS spectrum of compound **24**. 50

Figure S51. ^{1}H NMR and ^{13}C NMR spectra of compound **25**. 51

Figure S52. HSQC and HMBC spectra of compound **25**. 52

Figure S53. HRMS spectrum of compound **25**. 53

Figure S54. ^{1}H NMR and ^{13}C NMR spectra of compound **26**. 54

Figure S55. HSQC and HMBC spectra of compound **26**. 55

Figure S56. HRMS spectrum of compound **26**. 56

Relative fluorescence index curves for compounds 9 – 26 57

Figure S57. Relative fluorescence index (RFI) of compound **9**, reserpine (positive control) and DMSO (negative control) 57

Figure S58. RFI of compound **10**, reserpine (positive control) and DMSO (negative control). 57

Figure S59. RFI of compound **11**, reserpine (positive control) and DMSO (negative control). 58

Figure S60. RFI of compound **12**, reserpine (positive control) and DMSO (negative control). 58

Figure S61. RFI of compound **13**, reserpine (positive control) and DMSO (negative control). 59

Figure S62. RFI of compound **14**, reserpine (positive control) and DMSO (negative control). 59

Figure S63. RFI of compound **15**, reserpine (positive control) and DMSO (negative control). 60

Figure S64. RFI of compound **16**, reserpine (positive control) and DMSO (negative control). 60

Figure S65. RFI of compound **17**, reserpine (positive control) and DMSO (negative control). 61

Figure S66. RFI of compound **18**, reserpine (positive control) and DMSO (negative control). 61

Figure S67. RFI of compound **19**, reserpine (positive control) and DMSO (negative control). 62

Figure S68. RFI of compound **20**, reserpine (positive control) and DMSO (negative control). 62

Figure S69. RFI of compound **21**, reserpine (positive control) and DMSO (negative control). 63

Figure S70. RFI of compound **22**, reserpine (positive control) and DMSO (negative control). 63

Figure S71. RFI of compound **23**, reserpine (positive control) and DMSO (negative control). 64

Figure S72. RFI of compound **24**, reserpine (positive control) and DMSO (negative control). 64

Figure S73. RFI of compound **25**, reserpine (positive control) and DMSO (negative control). 65

Figure S74. RFI of compound **26**, reserpine (positive control) and DMSO (negative control). 65

Figure S75. Chalcones described in [10], used in the docking studies. 66

Docking studies 66

marine drugs

MDPI

NMR spectra and characterization of the compounds 1, 3, 4, 6, 9, 10, 13-16, 18, and 20.

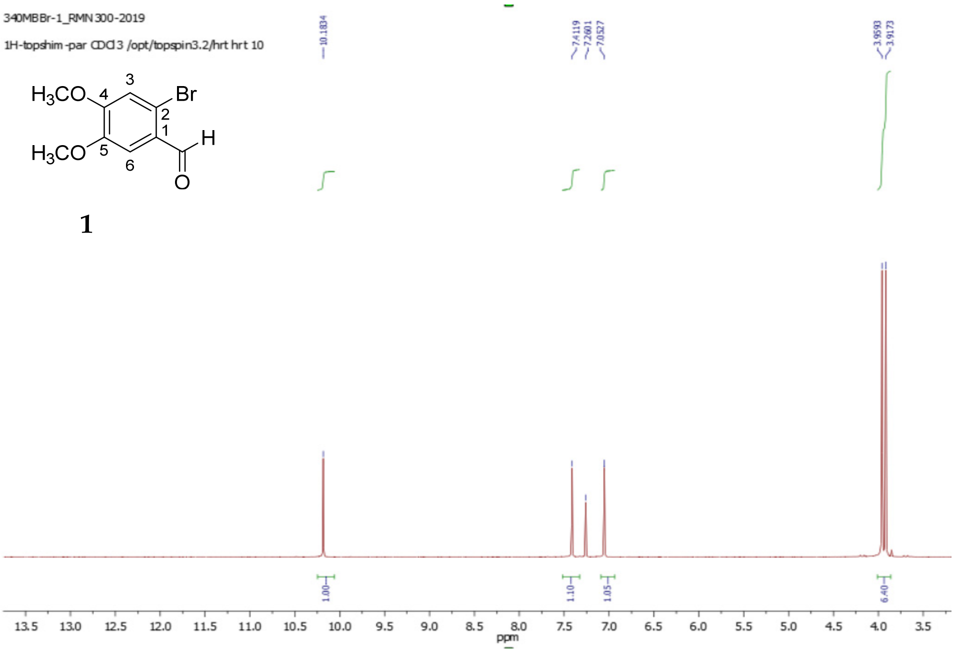

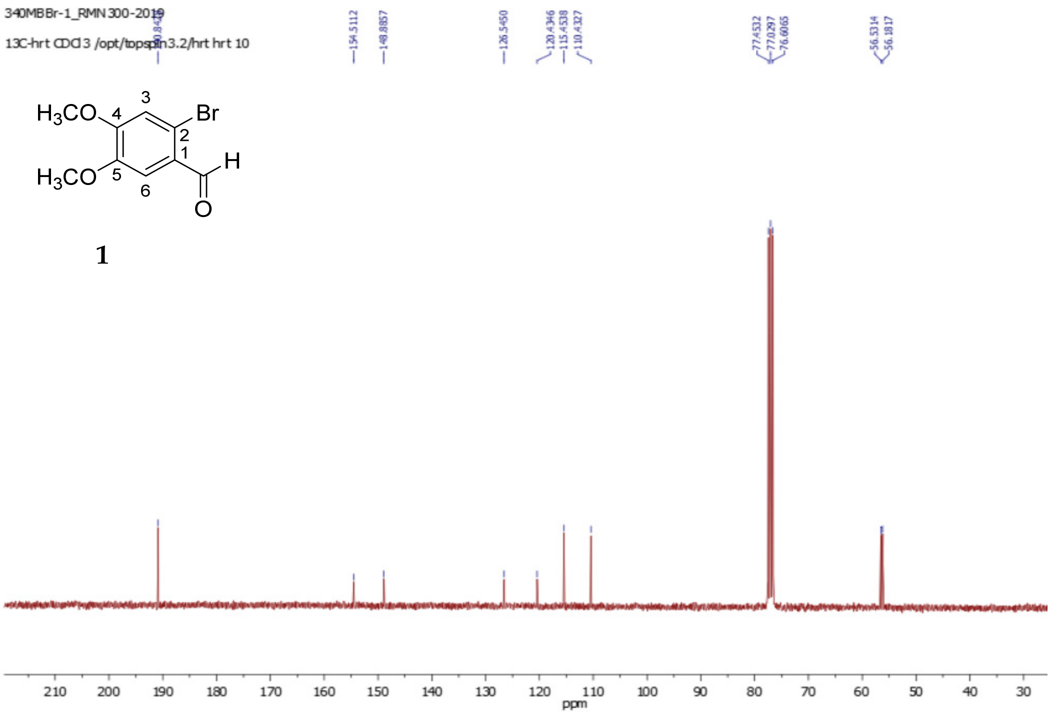

Figure S1. ^{1}H NMR and ^{13}C NMR spectra of compound **1**.

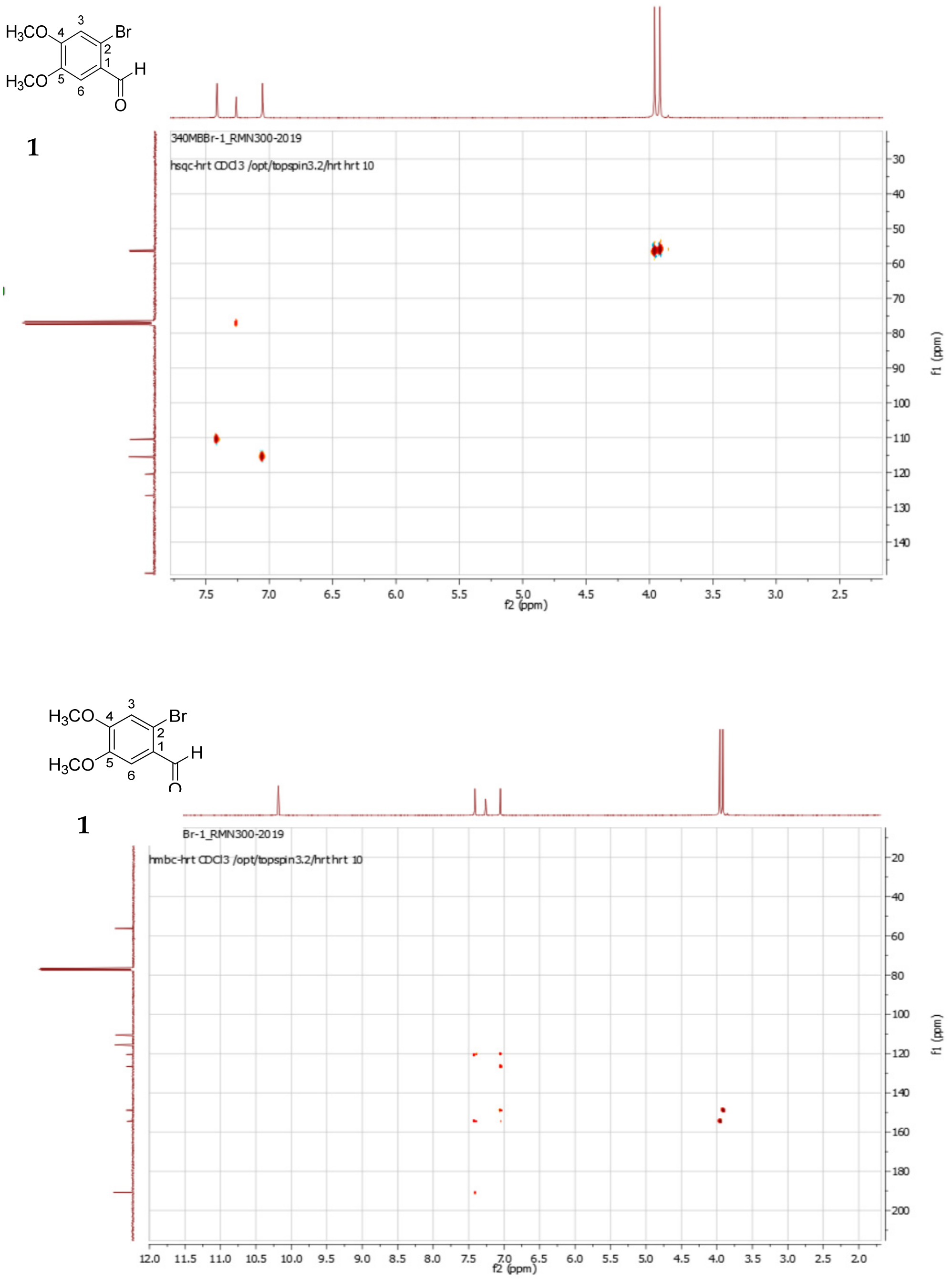

Figure S2. HSQC and HMBC spectra of compound **1**.

2-bromo-4,5-dimethoxybenzaldehyde (1): Purified by flash column chromatography (SiO2, n-hexane: ethyl acetate 9:1). Yield: 49% as a white solid; m.p.(n-hexane / ethyl acetate 9:1) = 150.7 - 151.5 °C; IR (KBr, υ (cm^{-1})): 3008 (Csp2-H); 2979, 2919, 2849 (Csp3-H); 1669 (C=O); 1590, 1507, 1469, 1445 (aromatic C=C), 1218 (C-O); 589 (C-Br); 1H NMR (300 MHz, CDCl3) δ (ppm): 10.18 (s, CHO), 7.41 (s, H-6), 7-05 (s, H-3), 3.96 (s, 5- OCH3), 3.92 (4-OCH_3); ^{13}C NMR (300 MHz, CDCl3) δ (ppm): 190.8 (C=O), 154.5 (C-5), 148.8 (C-4), 120.4 (C-2), 115.5 (C-3), 110.4 (C-6), 56.5 (5-OCH3), 56.2 (4-OCH3).

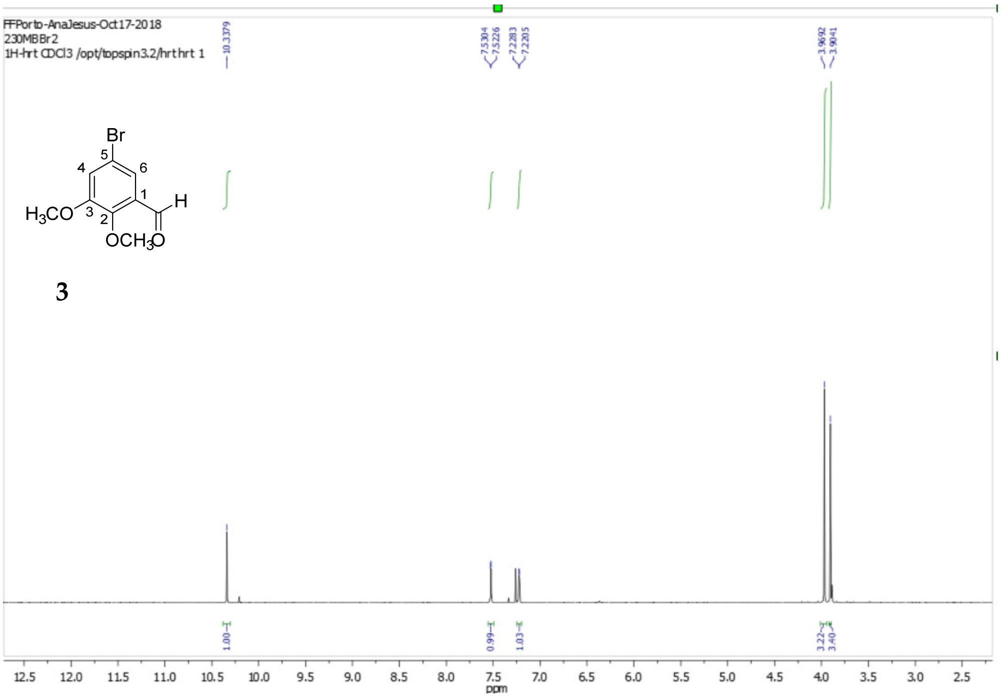

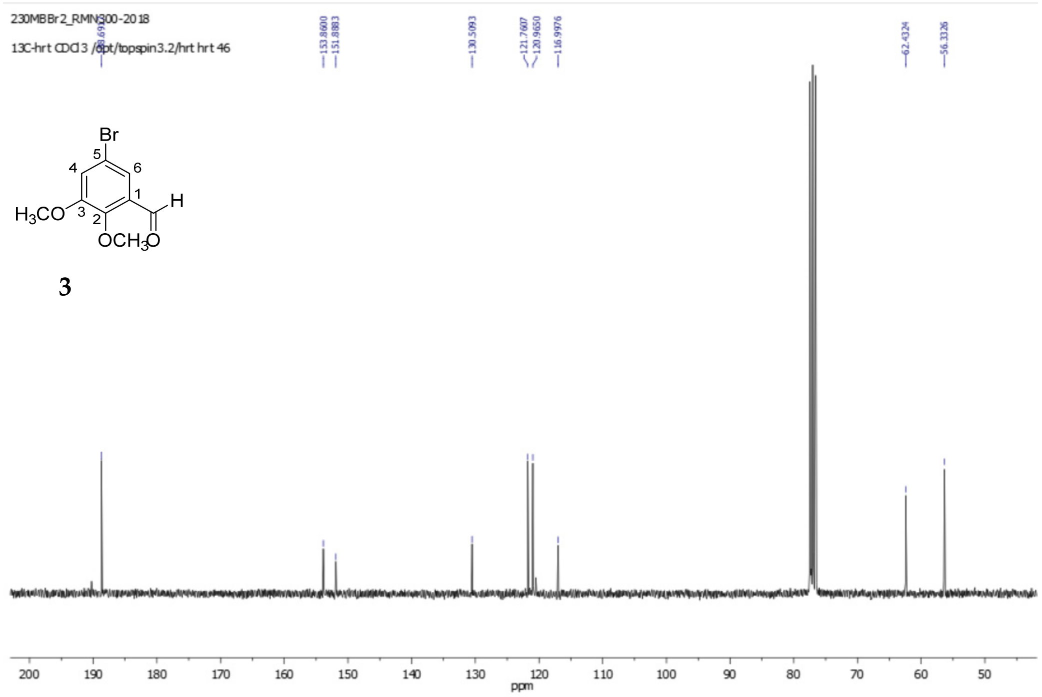

Figure S3. ^{1}H NMR and ^{13}C NMR spectra of compound **3**.

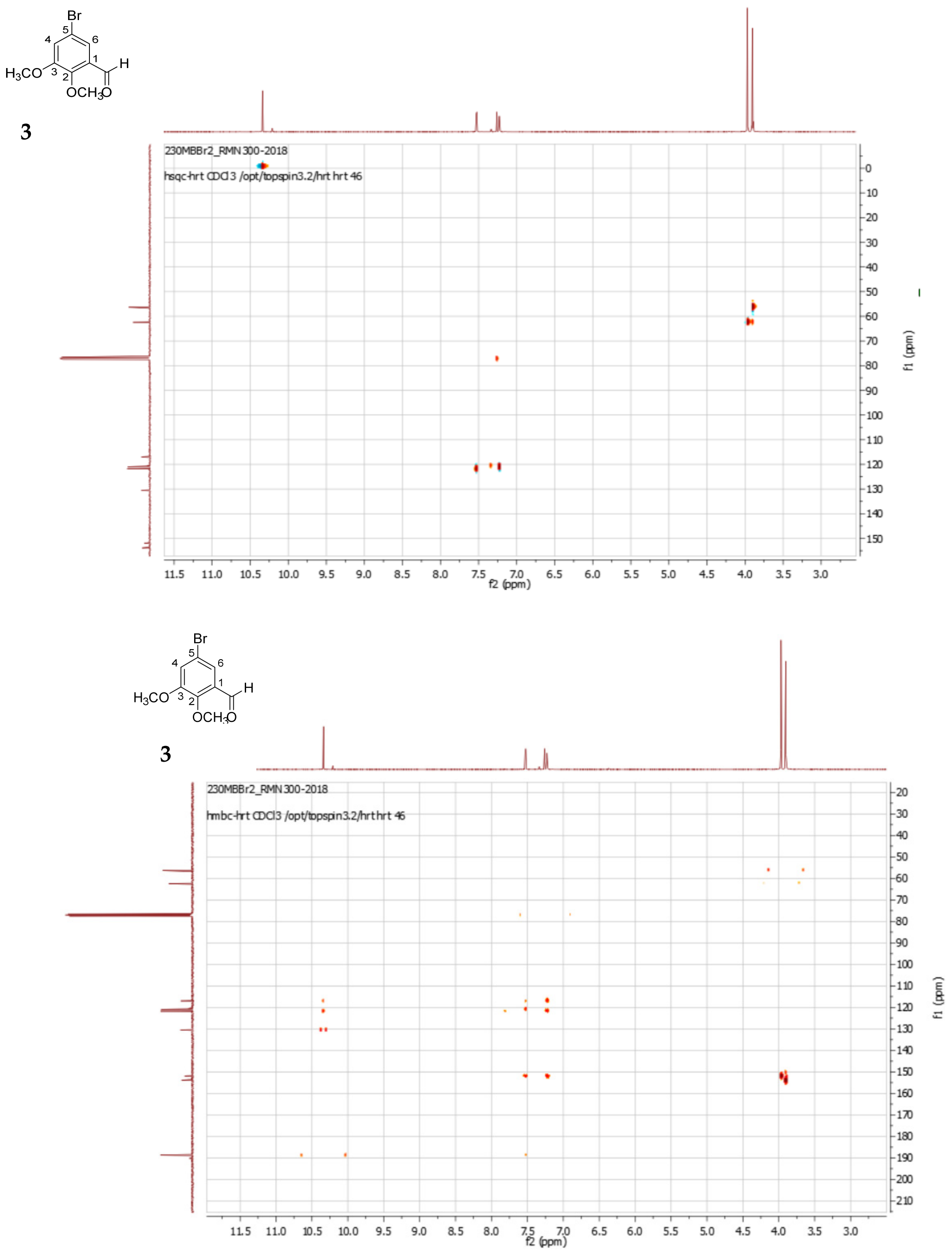

Figure S4. HSQC and HMBC spectra of compound **3**.

5-bromo-2,3-dimethoxybenzaldehyde (3): purified by flash column chromatography (SiO_2, n-hexane: ethyl acetate 9:1). Yield: 53% as white solid; m.p. (n-hexane/ethyl acetate 95:5) = 77.8 - 80.4°C; IR (KBr, υ (cm^{-1})): 3082 (C_{sp2}-H); 2966, 2939, 2847 (C_{sp3}-H); 1679 (C=O); 1577, 1482, 1445, 1429 (aromatic C=C); 1218 (C-O); 583 (C-Br); ^{1}H NMR (300 MHz, $CDCl_3$) δ (ppm): 10.34 (*s*, CHO), 7.53 (*d*, J = 2.3 Hz, H-4), 7.22 (*d*, J = 2.3 Hz, H-6), 3.97 (*s*, 2-OCH_3), 3.90 (*s*, 3-OCH_3); ^{13}C NMR (300 MHz, $CDCl_3$) δ (ppm): 188.7 (C=O), 153.9 (C-3), 151.9 (C-2), 121.8 (C-4), 121.0 (C-6), 117.0 (C-5), 62.4 (2-OCH_3), 56.3 (3-OCH_3).

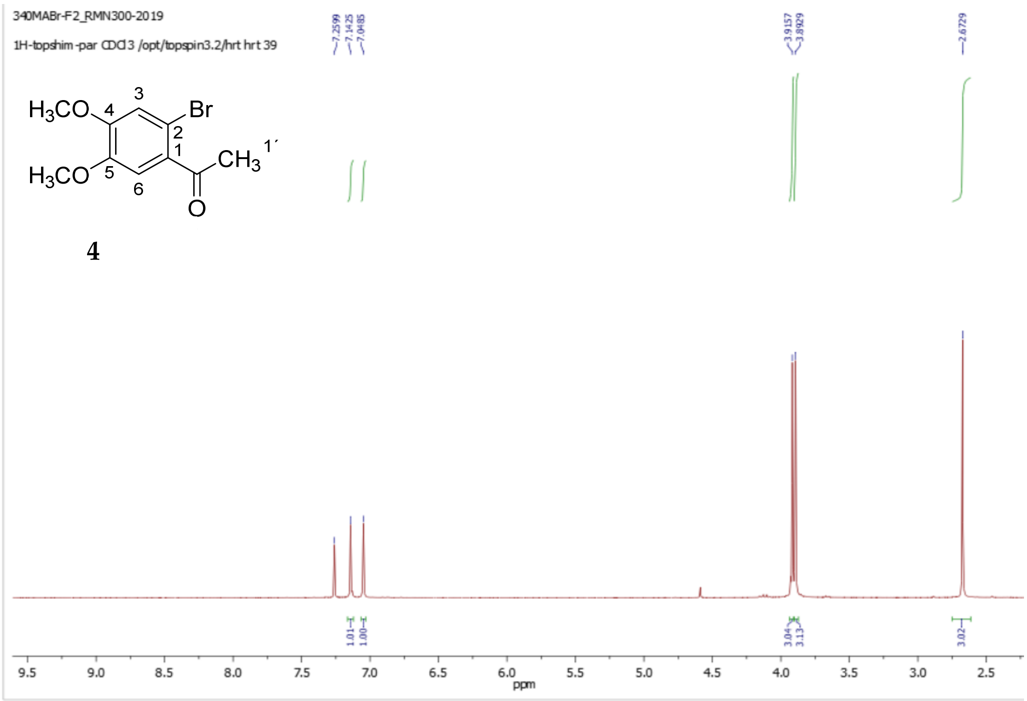

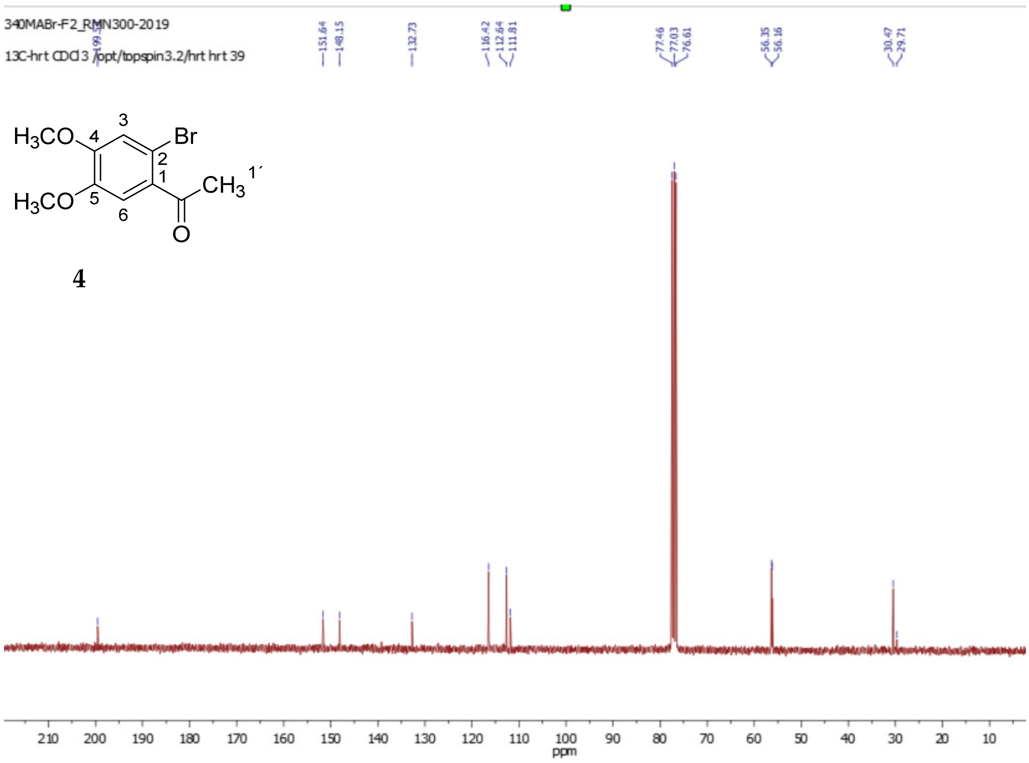

Figure S5. ^{1}H NMR and ^{13}C NMR spectra of compound **4**.

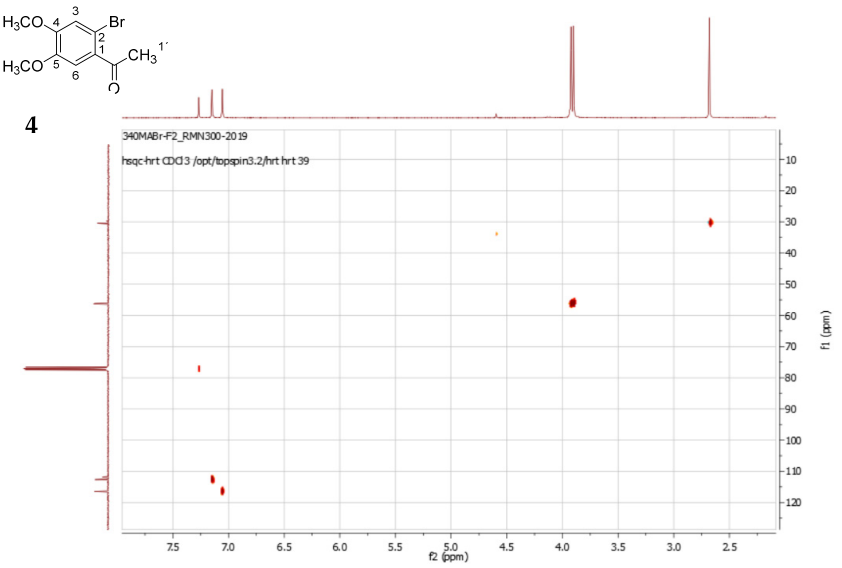

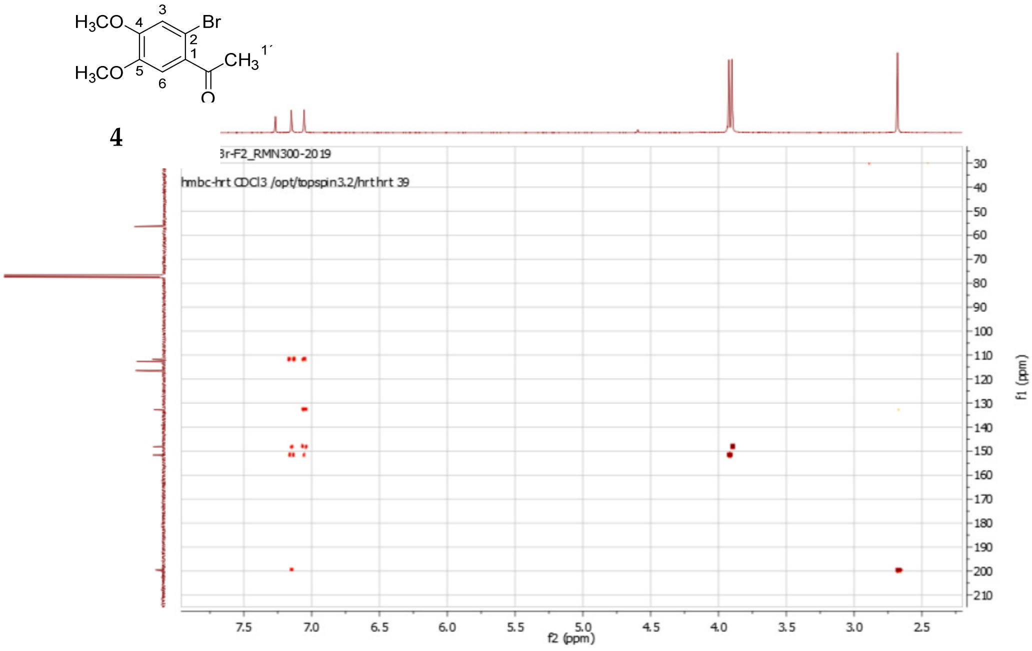

Figure S6. HSQC and HMBC spectra of compound **4**.

2-bromo-4,5-dimethoxyacetophenone (4): purified by flash column chromatography (SiO_2, n-hexane: ethyl acetate 9:1). Yield: 63% as white solid; m.p. (n-hexane/ethyl acetate 9:1) = 74.7 - 75.6 °C; IR (KBr, υ (cm^{-1})): 2967, 2937, 2841 (C_{sp3}-H); 1681 (C=O); 159, 1562, 1501, 1440 (aromatic C=C); 1204 (C-O); 649 (C-Br);^{1}H NMR (300 MHz, $CDCl_3$) δ (ppm): 7.14 (*s*, H-6), 7.05 (*s*, H-3), 3.92 (*s*, 5-OCH_3), 3.89 (4-OCH_3), 2.67 (*s*, H-1′); ^{13}C NMR (300 MHz, $CDCl_3$) δ (ppm): 199.5 (C=O), 151.6 (C-5), 148.2 (C-4), 132.7 (C-1), 116.4 (C-3), 112.6 (C-6), 111.8 (C-2), 56.4 (5-OCH_3), 56.2 (4-OCH_3, 30.5 (C-1′).

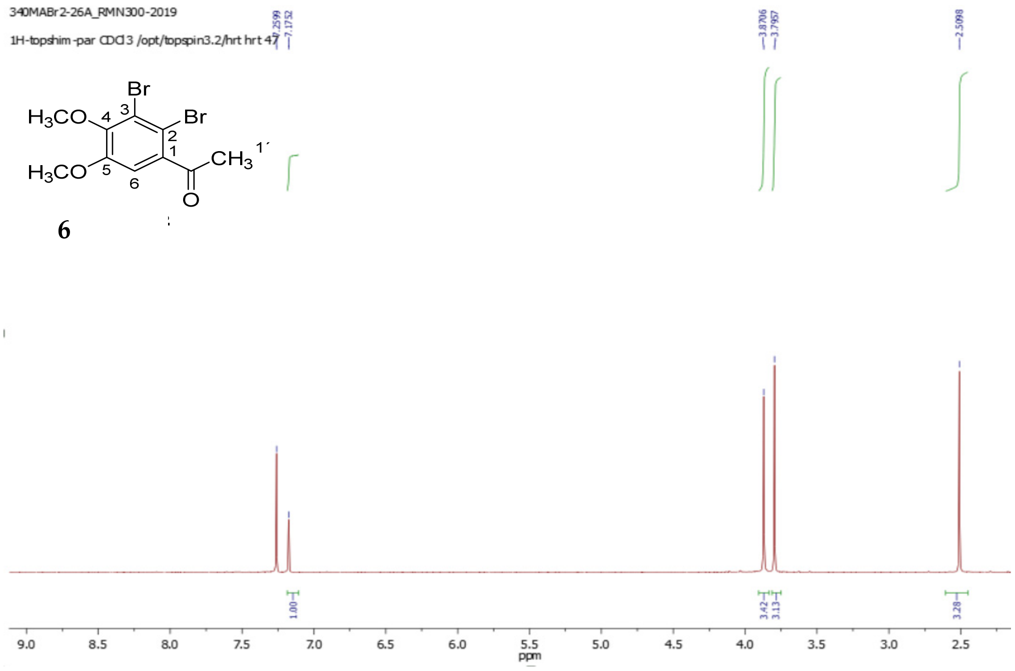

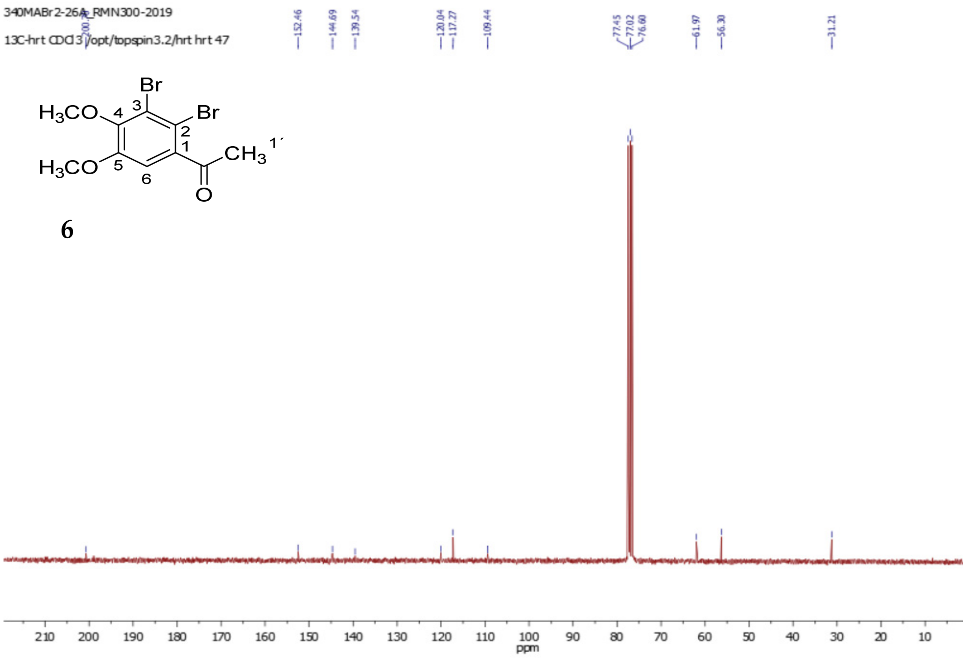

Figure S7. ^{1}H NMR and ^{13}C NMR spectra of compound **6**.

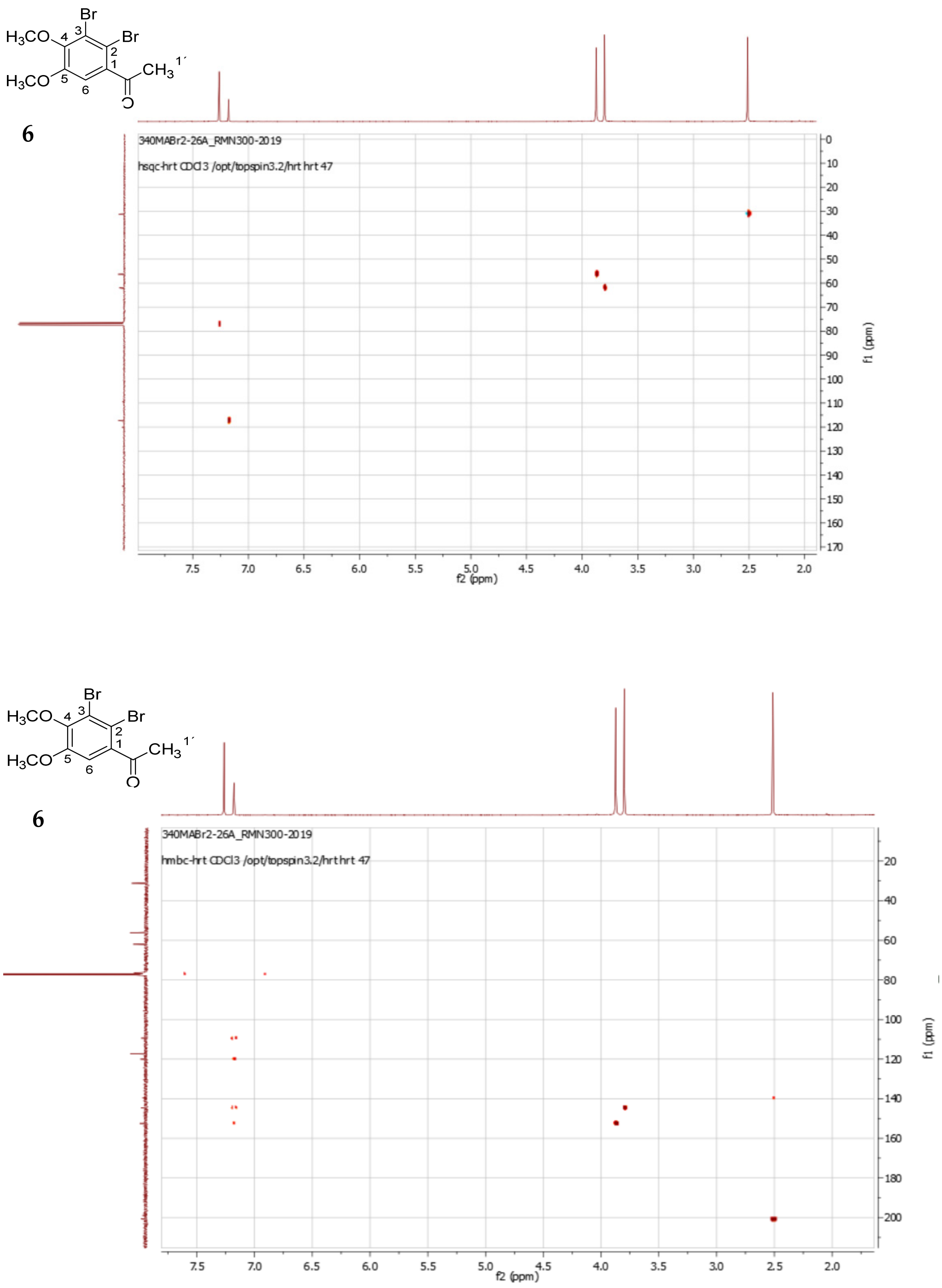

Figure S8. HSQC and HMBC spectra of compound **6**.

2,3-dibromo-4,5-dimethoxyacetophenone (6): purified by flash column chromatography (SiO_2, n-hexane: ethyl acetate 95:5). Yield: 1% as orange oil; ^1H NMR (300 MHz, $CDCl_3$) δ (ppm): 7.18 (*s*, H-6), 3.87 (*s*, 5-OCH_3), 3.80 (*s*, 4-OCH_3), 2.51 (*s*, H-1´); ^{13}C NMR (300 MHz, $CDCl_3$) δ (ppm): 200.7 (C=O), 152.5 (C-4), 144.7 (C-5), 139.5 (C-1), 120.0 (C-2), 117.3 (C-6), 109.3 (C-6), 62.0 (5-OCH_3), 56.3 (4-OCH_3), 31.2 (C-1´).

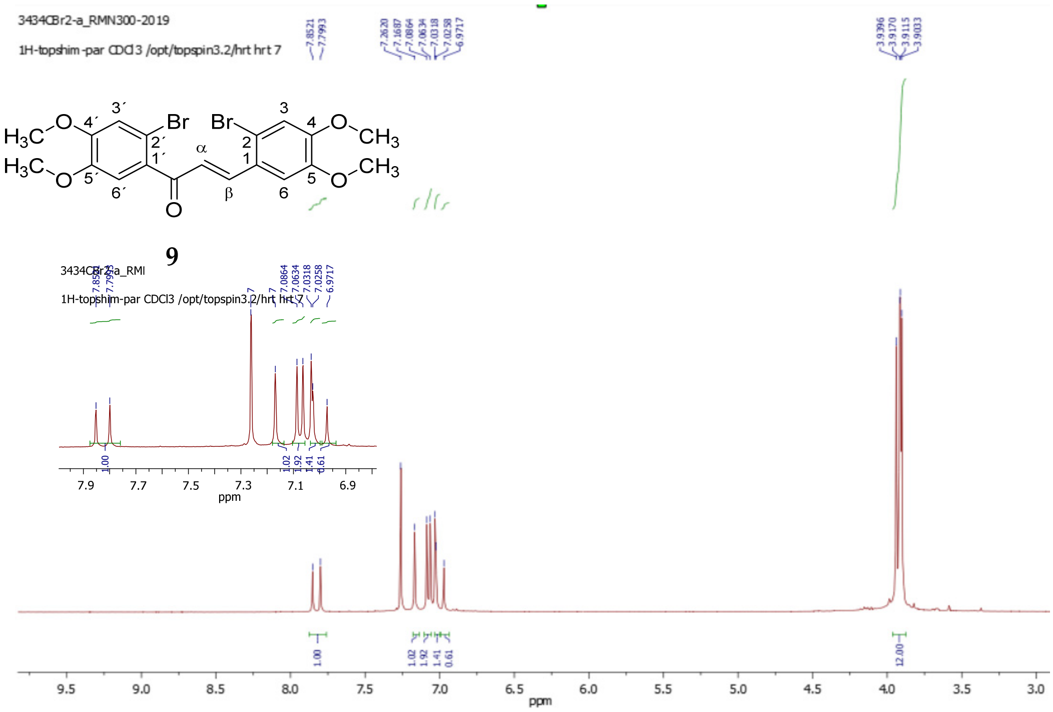

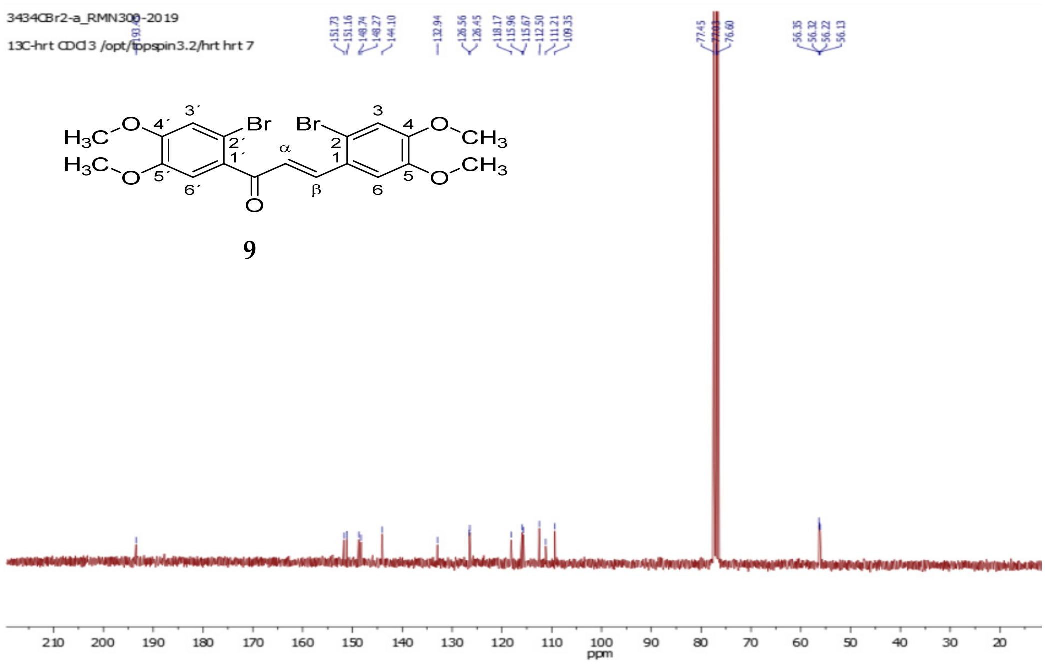

Figure S9. ^{1}H NMR and ^{13}C NMR spectra of compound **9**.

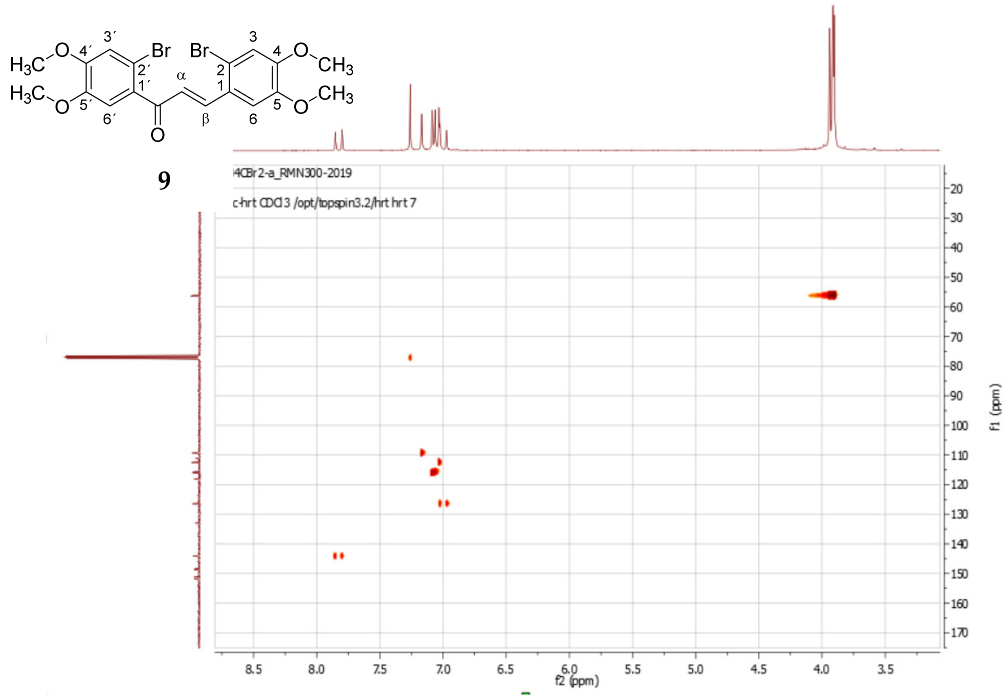

9

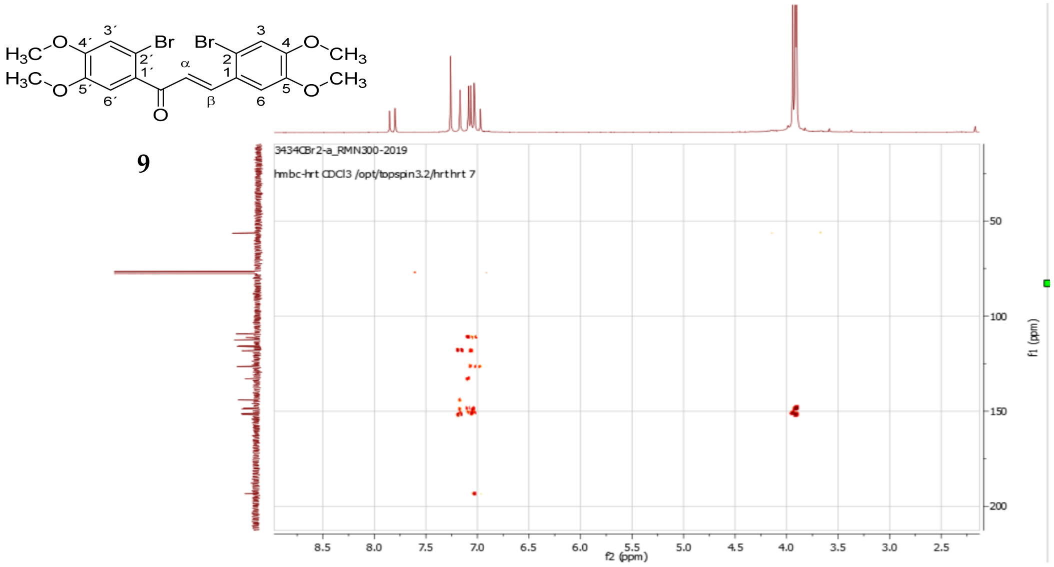

9

Figure S10. HSQC and HMBC spectra of compound **9**.

***(E)*-1,3-bis(2-bromo-4,5-dimethoxyphenyl)prop-2-en-1-one (9):** purified by flash column chromatography (SiO_2, n-hexane/ ethyl acetate 98:2), followed by TLC (SiO_2, diethyl ether/ petroleum ether 8:2). Yield: 87% as yellow solid; m.p.(diethyl ether/ petroleum ether 8:2) = 168.6 - 171.6 °C; IR (KBr, υ (cm^{-1})): 2932, 1840 (C_{sp3}-H); 1658 (C=O); 1591, 1505, 1465, 1442 (aromatic C=C); 1336 (*trans* vinylic system); 1202 (C-O); 587 (C-Br); ^{1}H NMR (300 MHz, $CDCl_3$) δ (ppm): 7.83 (*d*, J = 15.9 Hz, H-β), 7.17 (*s*, H-3), 7.09 (*s*, H-6´),7.06 (*s*, H-6), 7.03 (*s*, H-3´), 7.00 (*d*, J = 15.9 Hz, H-α), 3.94 (*s*, 5´-OCH_3), 3.92 (*s*, 4-OCH_3), 3.91 (*s*, 4´-OCH_3),3.90 (*s*, 5-OCH_3); ^{13}C NMR (300 MHz, $CDCl_3$) δ (ppm): 193.5 (C=O), 151.7 (C-4), 151.1 (C-5´), 148.7 (C-4´), 148.3 (C-5), 144.1 (C-β), 132.9 (C-1´), 126.6 (C-1), 126.5 (C-α), 118.1 (C-2), 116.0 (C-6´), 115.7 (C-6), 112.5 (C-3´), 111.2 (C-2´), 109.5 (C-3), 56.4 (4-OCH_3), 56.3 (5´-OCH_3), 56.2 (5-OCH_3), 56.1 (4´-OCH_3).

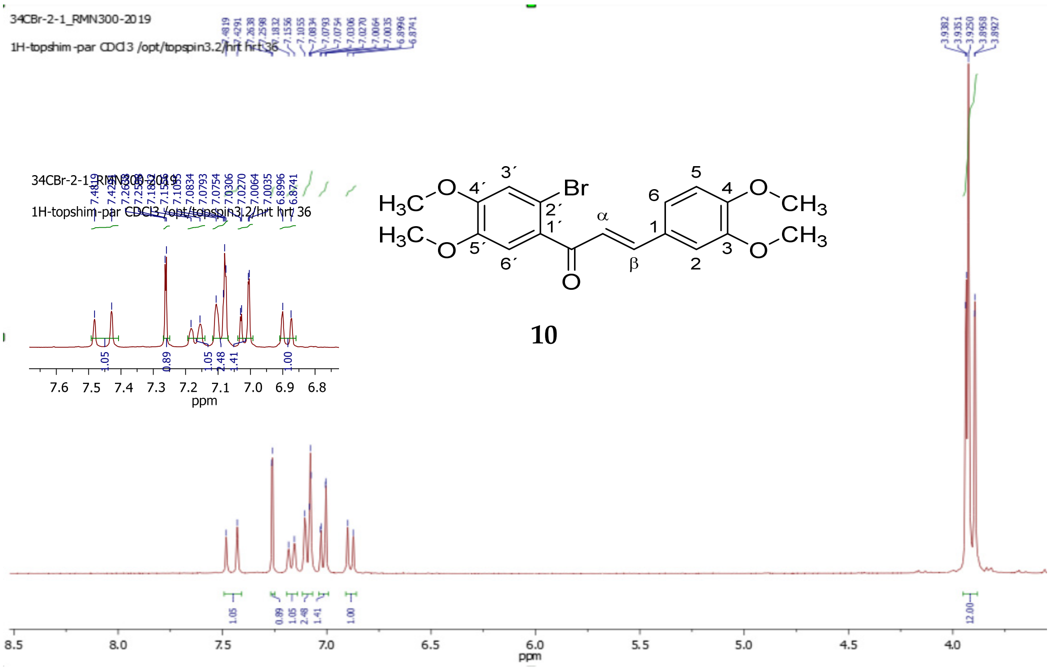

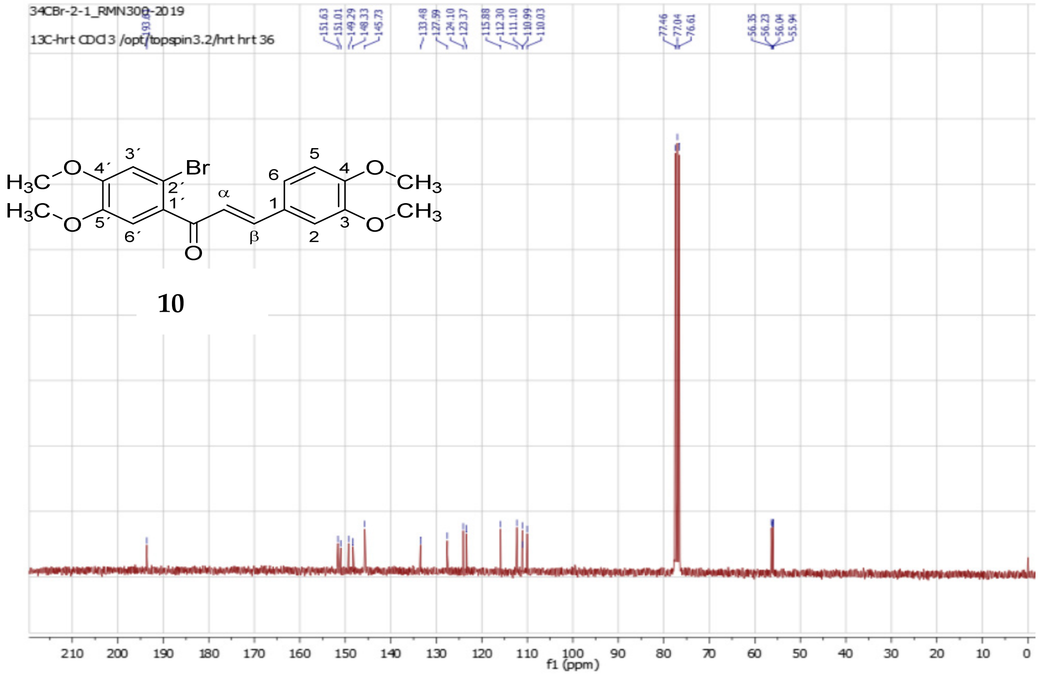

Figure S11. ^{1}H NMR and ^{13}C NMR spectra of compound **10**.

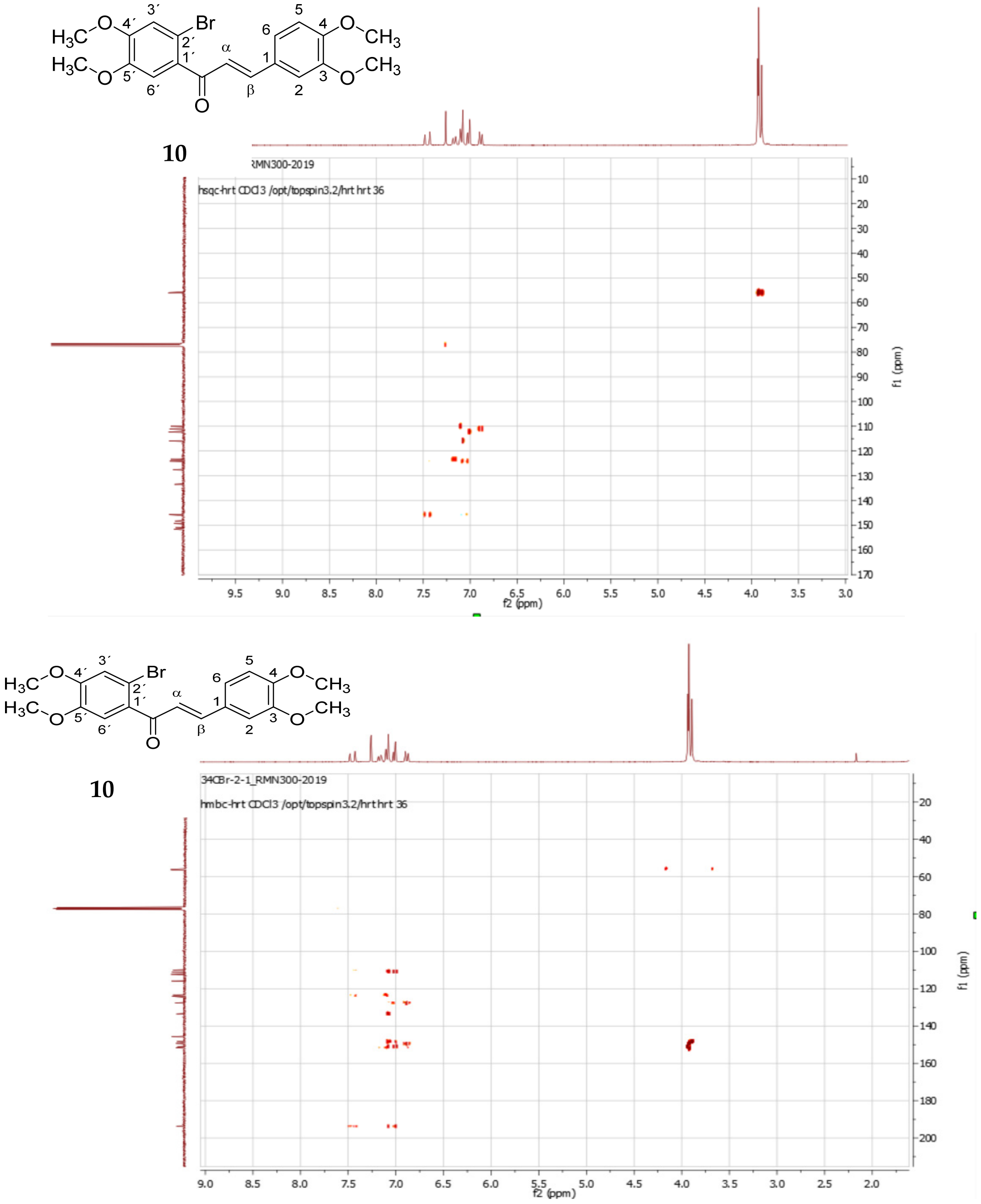

Figure S12. HSQC and HMBC spectra of compound **10**.

(*E*)-1-(2-bromo-4,5-dimethoxyphenyl)-3-(3,4-dimethoxyphenyl)prop-2-en-1-one (10): purified by TLC (SiO_2, n-hexane/ethyl acetate 9:1). Yield: 80% as yellow solid; m.p. (n-hexane/ethyl acetate 9:1) = 144.6-146.9 °C; IR (KBr, υ (cm^{-1})): 3084 (C_{sp2}-H); 2994, 2934, 2836 (C_{sp3}-H); 1638 (C=O); 1596, 1514, 1451, 1442 (aromatic C=C); 1372 (*trans* vinylic system); 1229 (C-O); 578 (C-Br); ^{1}H NMR (300 MHz, $CDCl_3$) δ (ppm): 7.45 (*d*, J = 15.8 Hz, H-β), 7.17 (*dd*, J = 8.2; 2.1 Hz, H-6), 7.10 (*d*, J=2.1 Hz, H-2), 7.08 (*s*, H-3′), 7.04 (*d*, J = 15.8 Hz, H-α), 7.00 (*s*, H-6′), 6.89 (*d*, J = 8.2 Hz, H-5), 3.93 (*s*, 4-OCH_3), 3.92 (*s*, 3-OCH_3, 5′-OCH_3), 3.89 (*s*, 4′-OCH_3); ^{13}C NMR (300 MHz, $CDCl_3$) δ (ppm): 193.6 (C=O), 151.6 (C-5′), 151.0 (C-4),149.3 (C-3), 148.3 (C-4′), 145.7 (C-β), 133.5 (C-1′), 127.6 (C-α), 124.1 (C-6), 123.4 (C-1), 115.9 (C-3′), 112.3 (C-6′), 111.1 (C-2′), 111.0 (C-5), 110.0 (C-2), 56.4 (5′-OCH_3), 56.2 (4′-OCH_3), 56.0 (3-OCH_3), 55.9 (4-OCH_3).

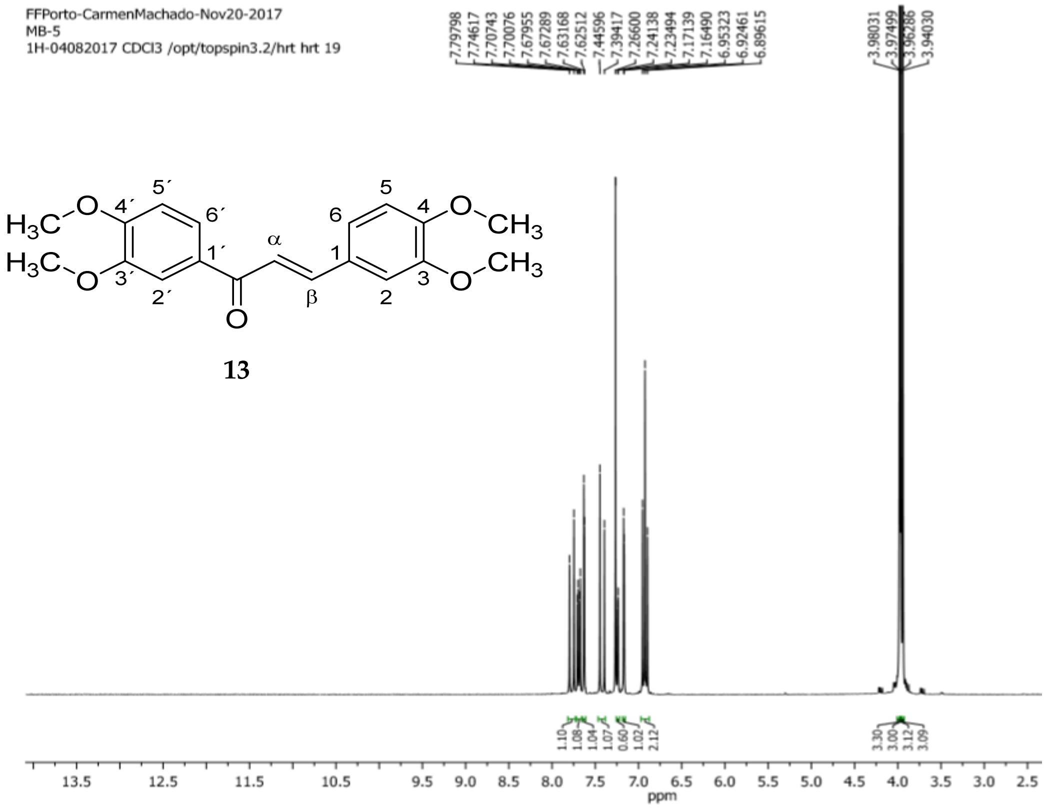

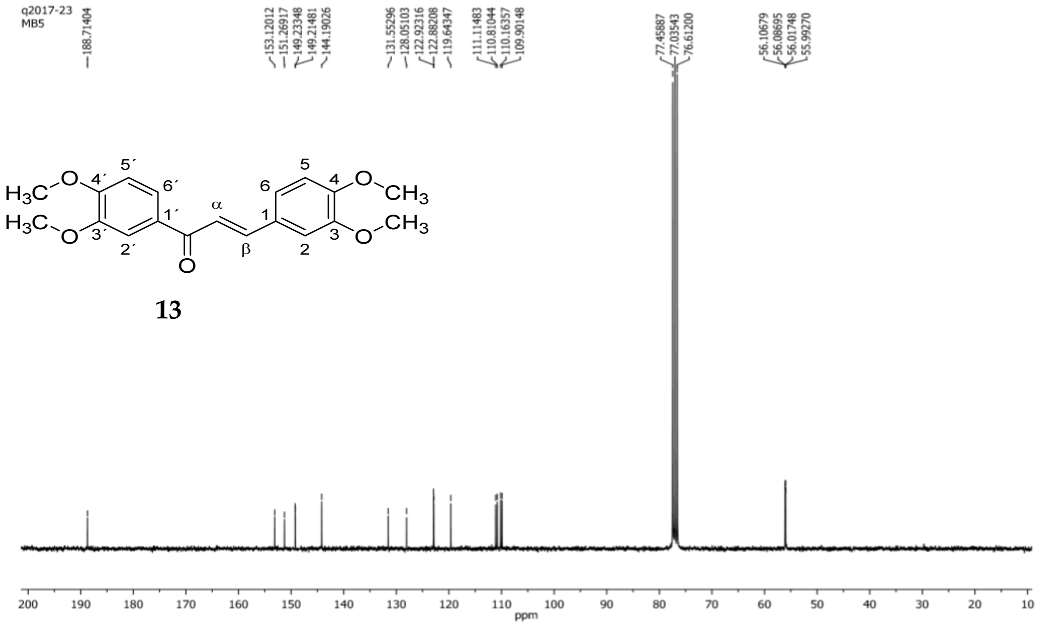

Figure S13. ^{1}H NMR and ^{13}C NMR spectra of compound **13**.

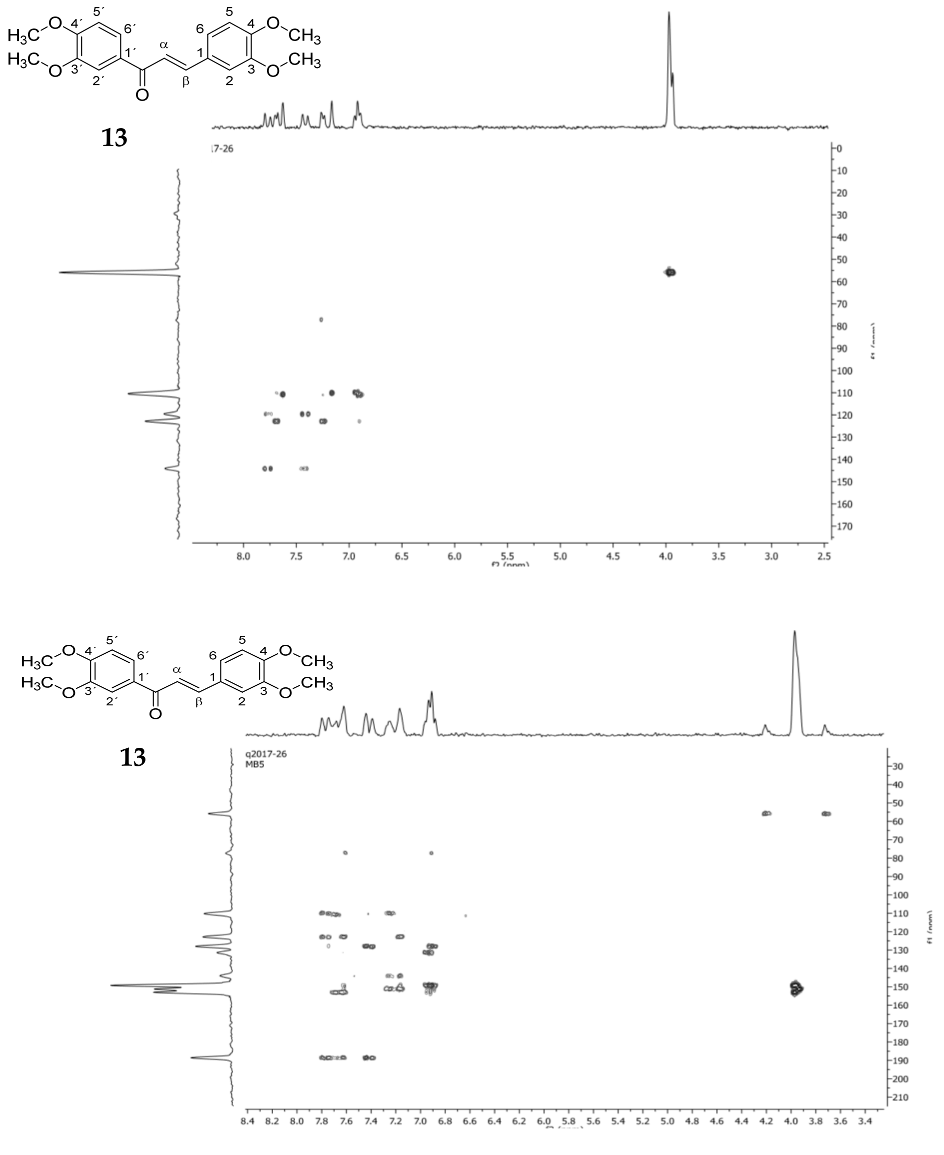

Figure S14. HSQC and HMBC spectra of compound **13**.

(*E*)-1,3-bis(3,4-dimethoxyphenyl)prop-2-en-1-one (13): Yield: 74% as a yellow solid; m.p. (distilled water) = 89.3 - 91.1 °C; IR (KBr, υ (cm^{-1})): 3082, 3008 (C_{sp2}-H); 2937, 2839 (C_{sp3}-H);1651 (C=O); 1597, 1574. 1510, 1464 (aromatic C=C); 1348 (*trans* vinylic system); 1200 (C-O); ^{1}H NMR (300 MHz, $CDCl_3$) δ (ppm): 7.77 (*d*, J = 15.5 Hz, H-β), 7.69 (*dd*, J = 8.5; 2.0 Hz, H-6′), 7.63 (*d*, J = 2.0 Hz, H-2′), 7.42 (*d*, J = 15.5 Hz, H-α), 7.25 (*dd*, J = 8.4; 1.9 Hz, H-6), 7.17 (*d*, J = 1.9 Hz, H-2), 6.94 (*d*, J = 8.5 Hz, H-5′), 6.91 (*d*, J = 8.4 Hz, H-5), 3.98 (*s*, 4′-OCH_3; 4-OCH_3), 3.96 (*s*, 3-OCH_3), 3.94 (*s*, 3′-OCH_3); ^{13}C NMR (300 MHz, $CDCl_3$) δ (ppm): 188.7 (C=O), 153.1 (C-4′), 151.3 (C-4), 149.2 (C-3), 149.2 (C-3′), 144.2 (C-β), 131.6 (C-1′), 128.1 (C-1), 122.9 (C-6′; C-6), 119.6 (C-α), 111.1 (C-5), 110.8 (C-2′), 110.2 (C-2), 109.9 (C-5′), 56.1 (3′-OCH_3, 3-OCH_3), 56.0 (4-OCH_3), 55.9 (4′-OCH_3).

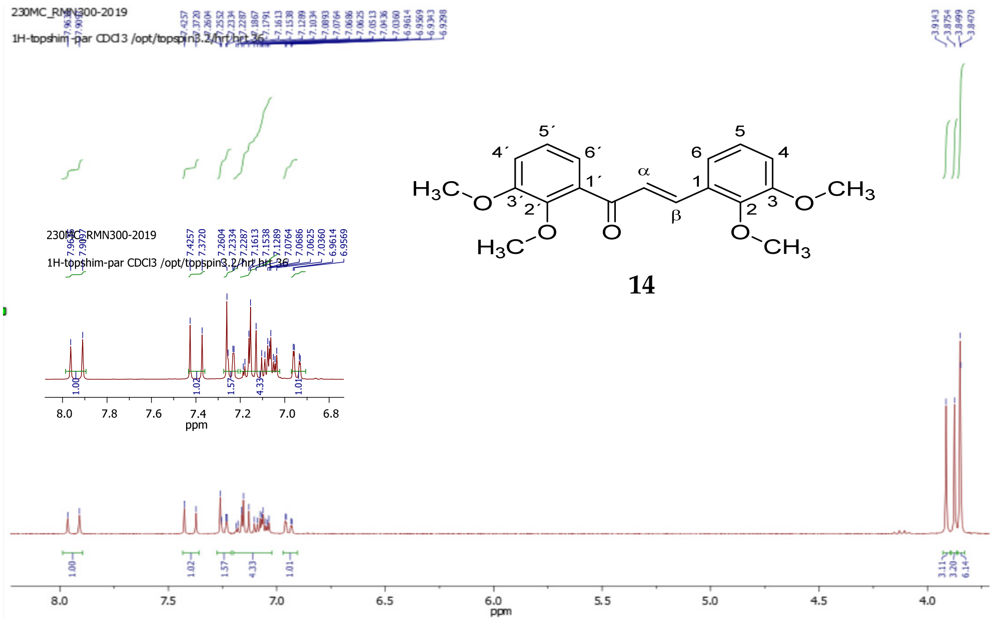

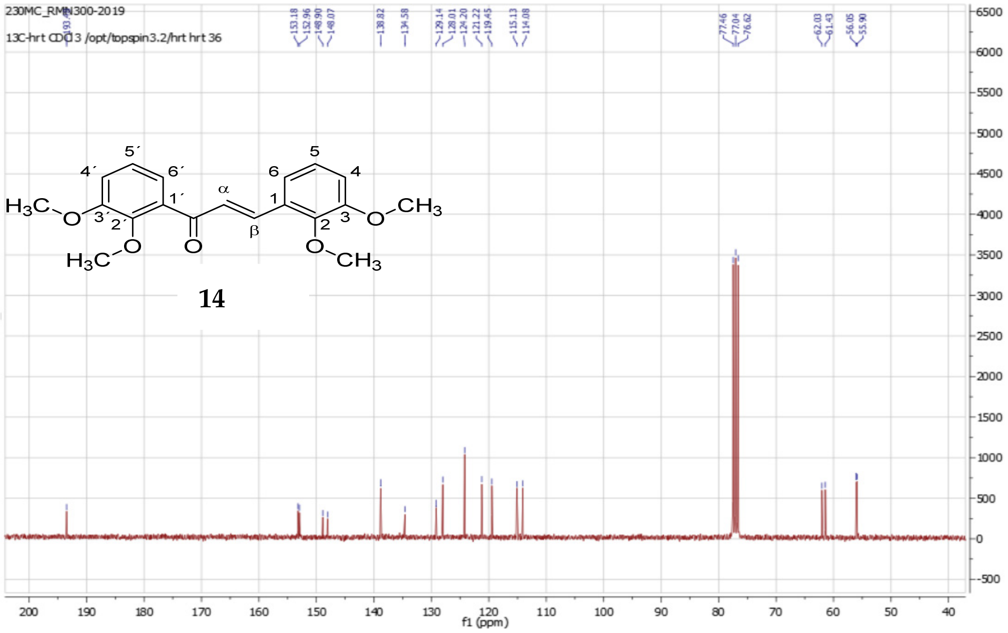

Figure S15. ^{1}H NMR and ^{13}C NMR spectra of compound **14**.

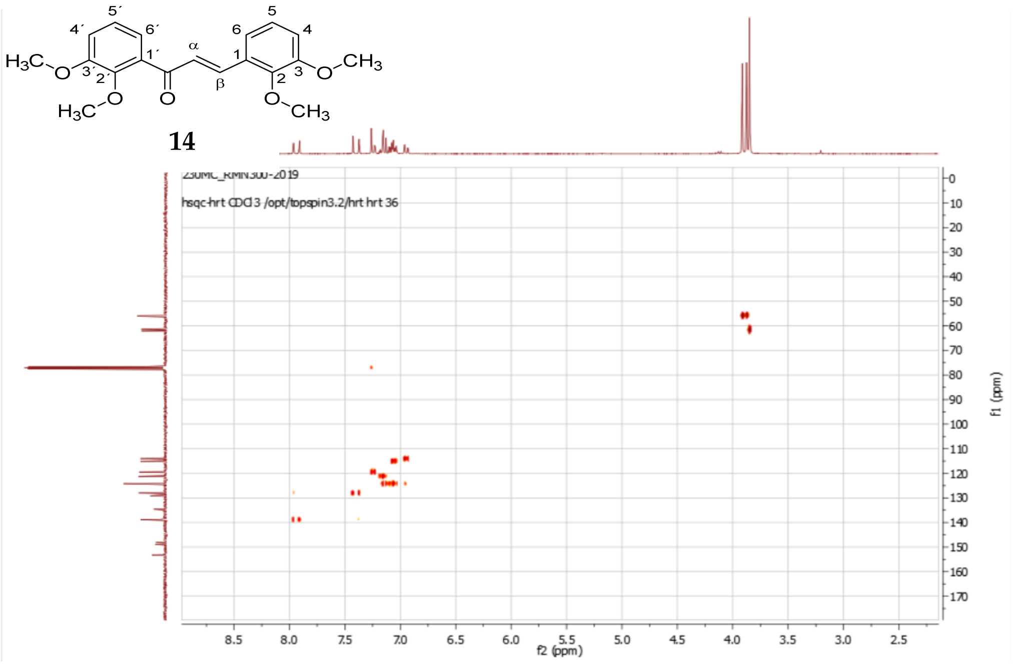

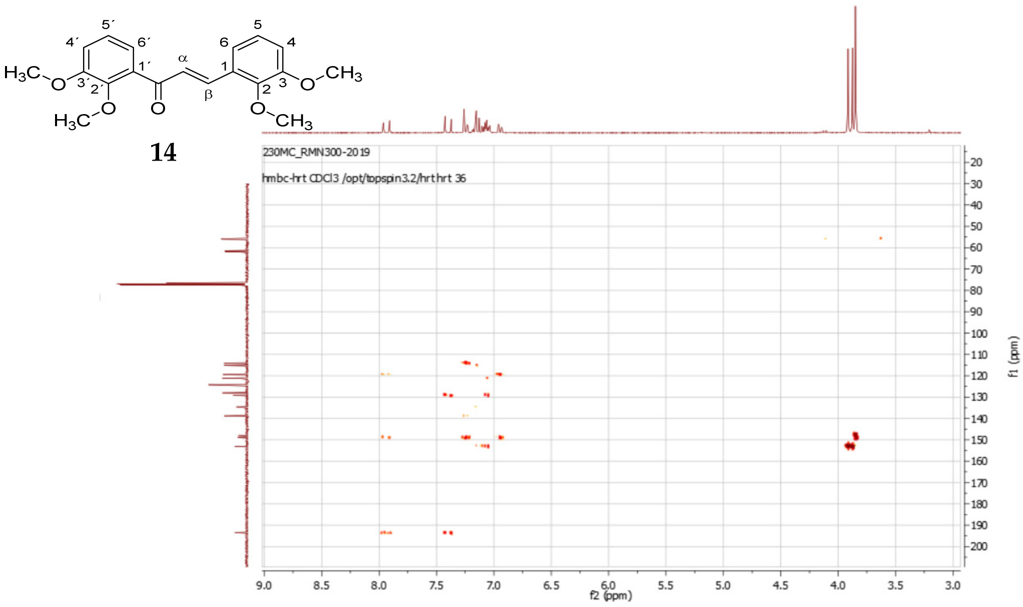

Figure S16. ^{1}H NMR and ^{13}C NMR spectra of compound **14**.

(*E*)-1,3-bis(2,3-dimethoxyphenyl)prop-2-en-1-one (14): purified by flash column chromatography (SiO_2, n-hexane/ ethyl acetate 9:1). Yield: 85% as an orange oil; IR (KBr, υ (cm^{-1})): 3075 (C_{sp2}-H); 2937, 2836 (C_{sp3}-H); 1735 (C=O); 1652, 1576, 1474, 1426 (aromatic C=C); 1372 (*trans* vinylic system); 1170 (C-O); ^{1}H NMR (300 MHz, $CDCl_3$) δ (ppm): 7.94 (*d*, J = 16.1 Hz, H-β), 7.40 (*d*, J = 16.1 Hz, H-*α*), 7.24 (*dd*, J = 8.0; 2.2 Hz, H-6), 7.17 (*dd*, J = 7.7; 2.3 Hz, H-6), 7.13 (*t*, J = 7.7 Hz, H-5′), 7.06 (*t*, J = 8.0 Hz, H-5), 7.06 (*dd*, J = 7.7; 2.3 Hz, H-4′), 6.95 (*dd*, J = 8.0; 2.2 Hz, H-4), 3.91 (*s*, 3-OCH_3), 3.88 (*s*, 3′-OCH_3), 3.85 (*s*, 2-OCH_3, 2′-OCH_3); ^{13}C NMR (300 MHz, $CDCl_3$) δ (ppm): 193.5 (C=O), 153.2 (C-3′), 153.0 (C-3),148.9 (C-2′), 148.1 (C-2), 138.2 (C-β), 134.6 (C-1′), 129.1 (C-1), 128.0 (C-*α*), 124.2 (C-5′,C-5), 121.1 (C-6′), 119.5 (C-6), 115.1 (C-4′), 114.1 (C-4), 62.0 (2′-OCH_3), 61.4 (2-OCH_3), 56.1 (3-OCH_3), 55.9 (3′-OCH_3).

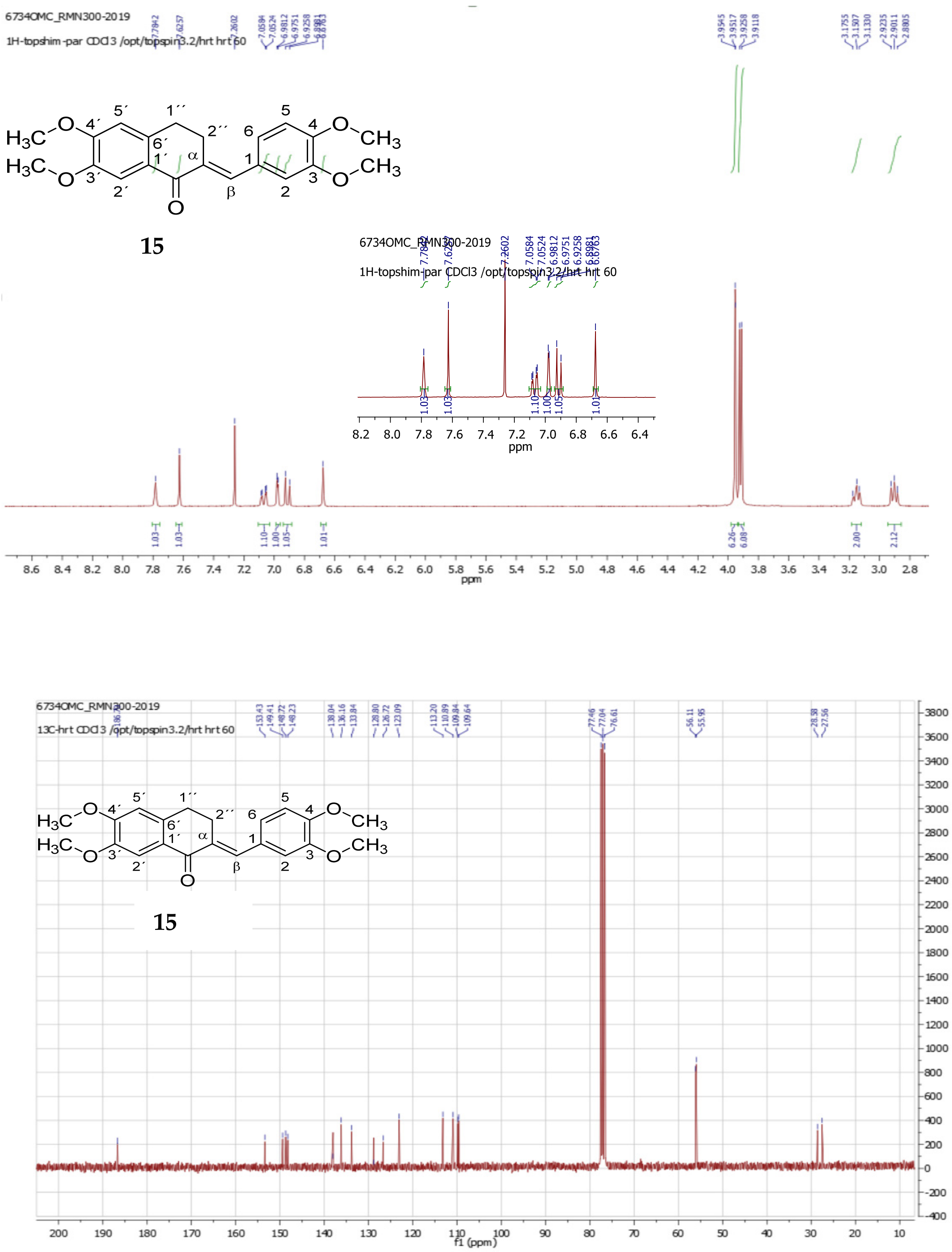

Figure S17. ^{1}H NMR and ^{13}C NMR spectra of compound **15**.

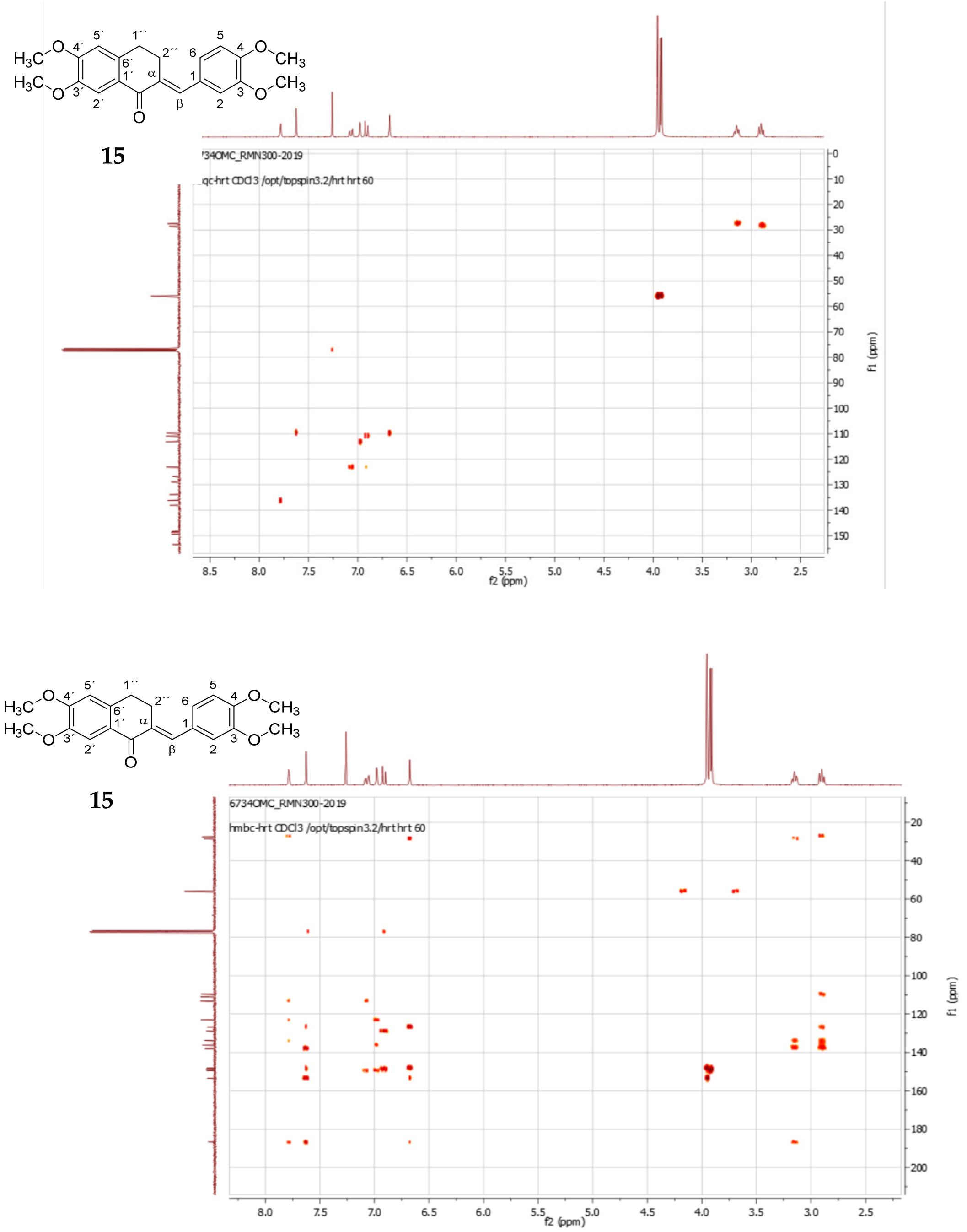

Figure S18. HSQC and HMBC spectra of compound **15**.

(*E*)-2-(3,4-dimethoxybenzylidene)-6,7-dimethoxy-3,4-dihydronaphthalen-1(2*H*)-one (15): Yield: 54% as a yellow solid; m.p. (distilled water) = 142.9 - 144 °C; IR (KBr, υ (cm^{-1})): 2990, 2937, 2837 (C_{sp3}-H);1657 (C=O); 1584, 1514, 1466, 1450 (aromatic C=C); 1362 (*trans* vinylic system); 1204 (C-O); ^{1}H NMR (300 MHz, $CDCl_3$) δ (ppm): 7.78 (*s*, H-β), 7.63 (*s*, H-2´), 7.07 (*dd*, J = 8.3; 1.9; Hz, H-6), 6.98 (*d*, J = 1.9 Hz, H-2), 6.91 (*d*, J = 8.3 Hz, H-5), 6.68 (*s*, H-5´), 3.95 (*s*, 3´-OCH_3), 3.94 (*s*, 4´-OCH_3), 3.92 (*s*, 4-OCH_3), 3.91 (*s*, 3-OCH_3), 3.15 (*t*, J = 6.5 Hz, H-1´´), 2.90 (*t*, J = 6.5 Hz, H-2´´); ^{13}C NMR (300 MHz, $CDCl_3$) δ (ppm): 186.7 (C=O), 153.4 (C-4´), 149.4 (C-4), 148.7 (C-3), (148.2 (C-3´), 138.0 (C-1´), 136.2 (C-β), 133.8 (C-6´), 128.8 (C-1), 126.7 (C-α), 123.1 (C-6), 113.2 (C-2), 110.9 (C-5), 109.6 (C-2´), 109.6 (C-5´), 56.1 (3´-OCH_3, 4´-OCH_3), 56.0 (3-OCH_3, 4-OCH_3), 28.6 (C-2´´), 27.6 (C-1´´).

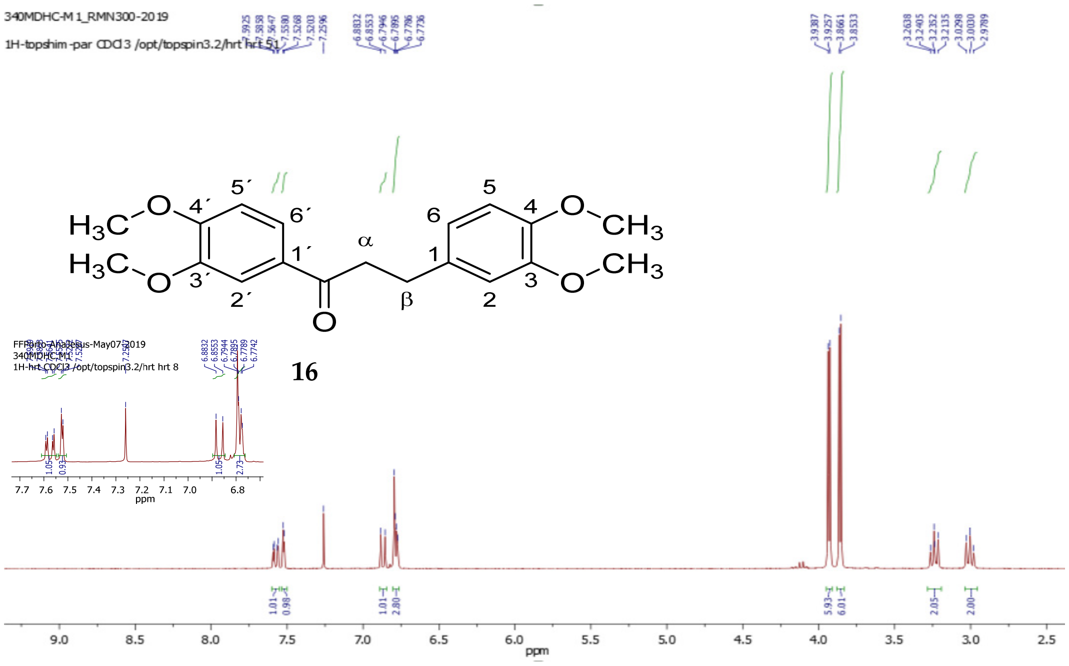

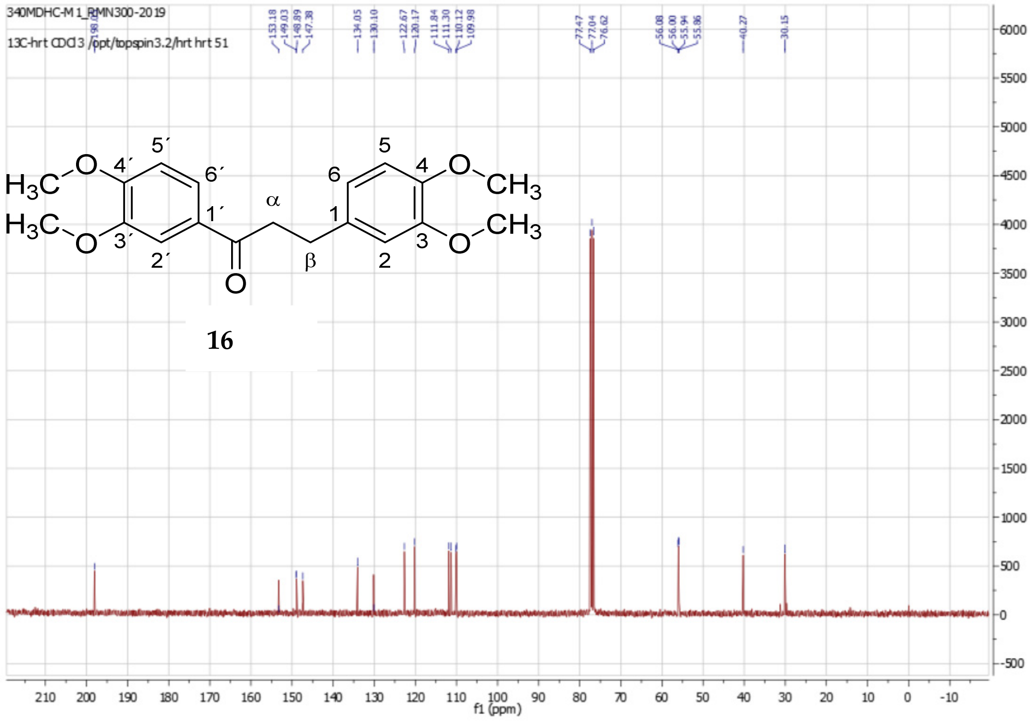

Figure S19. ^{1}H NMR and ^{13}C NMR spectra of compound **16**.

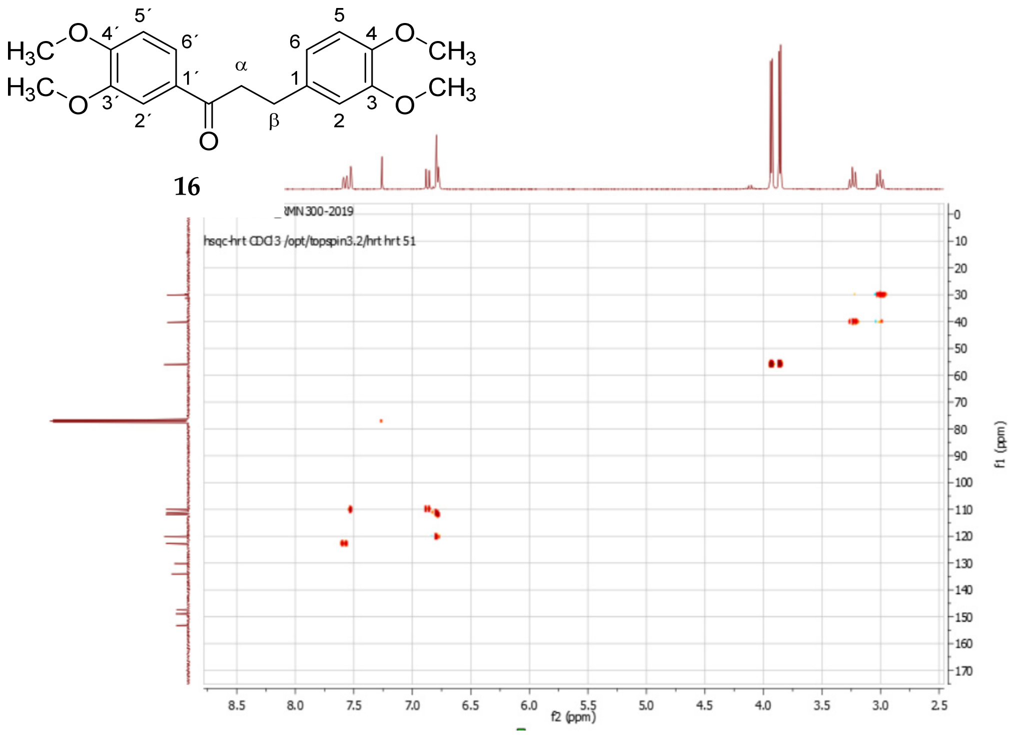

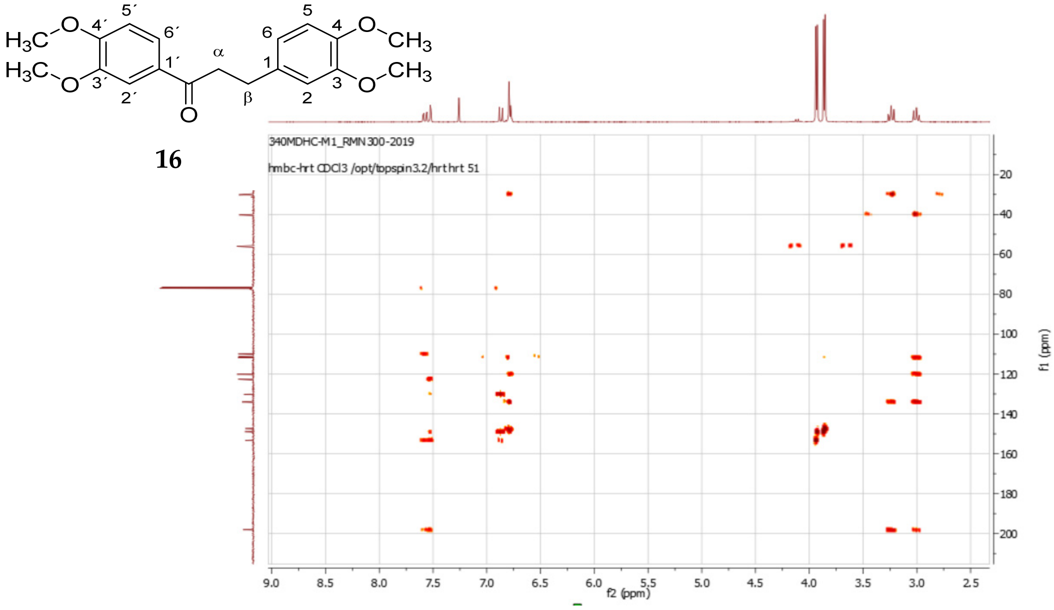

Figure S20. HSQC and HMBC spectra of compound **16**.

1,3-bis(3,4-dimethoxyphenyl)propan-1-one (16): purified by TLC (SiO_2, n-hexane/ ethyl acetate 6:4). Yield: 63% as a white solid; m.p. (n-hexane/ ethyl acetate 6:4) = 86.8 — 87.9 °C; IR (KBr, υ (cm^{-1})): 3000 (C_{sp2}-H); 2964, 2938, 2839 (C_{sp3}-H); 1668 (C=O); 1591, 1515, 1470, 1456 (aromatic C=C), 1198 (C-O); ^{1}H NMR (300 MHz, $CDCl_3$) δ (ppm): 7.59 (*d*, J = 2.0 Hz, H-2´), 7.58 (*dd*, J = 8.4; 2.0 Hz, H-6´), 6.87 (*d*, J = 8.4 Hz, H-5´), 6.81 (*d*, J = 8.0 Hz, H-5), 6.78 (*d*, J = 2.6 Hz, H-2), 6.78 (*dd*, J = 8.0; 2.6 Hz, H-6), 3.94 (*s*, 4´-OCH_3), 3.93 (*s*, 4-OCH_3), 3.87 (*s*, 3´-OCH_3), 3.85 (*s*, 3-OCH_3), 3.24 (*t*, J = 7.6 Hz, H-β), 3.00 (*t*, J = 7.6 Hz, H-α); ^{13}C NMR (300 MHz, $CDCl_3$) δ (ppm): 198.1 (C=O), 153.2 (C-4´), 149.0 (C-4), 148.9 (C-3´), 147.4 (C-3), 134.1 (C-1´), 130.1 (C-1), 122.7 (C-6´), 120.1 (C-2), 111.8 (C-6), 111.3 (C-5), 110.1 (C-2´), 110.0 (C-5´), 56.1 (4´-OCH_3; 4-OCH_3), 56.0 (3-OCH_3), 55.9 (3´-OCH_3), 40.3 (C-β), 30.2 (C-α).

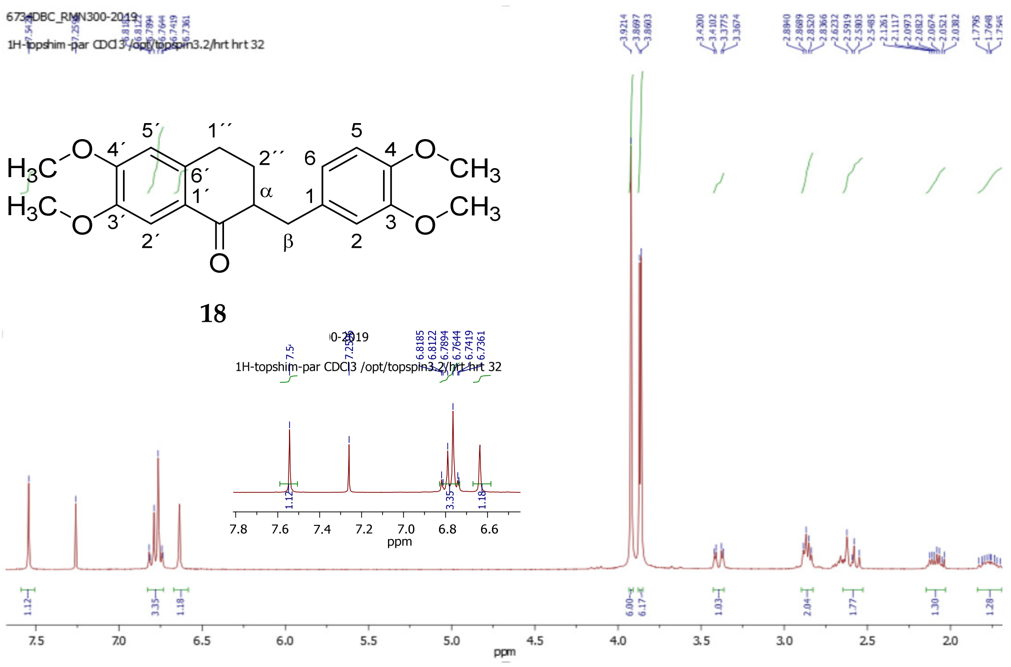

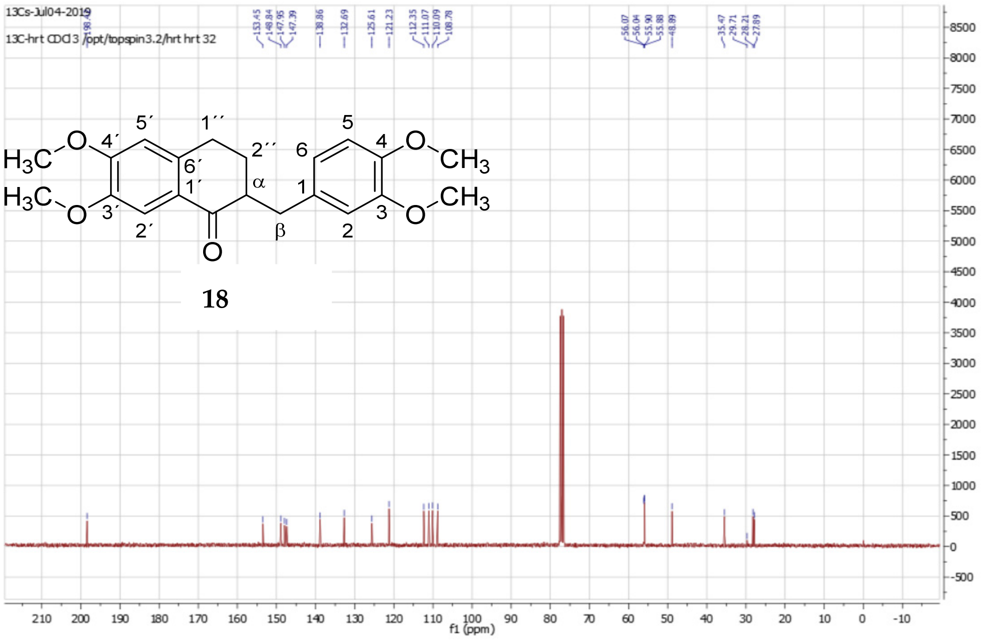

Figure S21. ^{1}H NMR and ^{13}C NMR spectra of compound **18**.

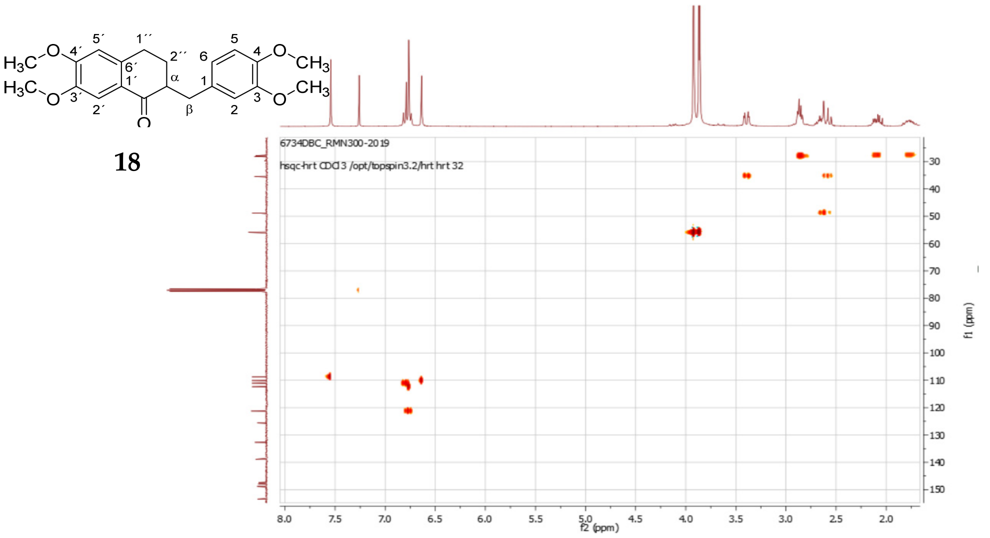

18

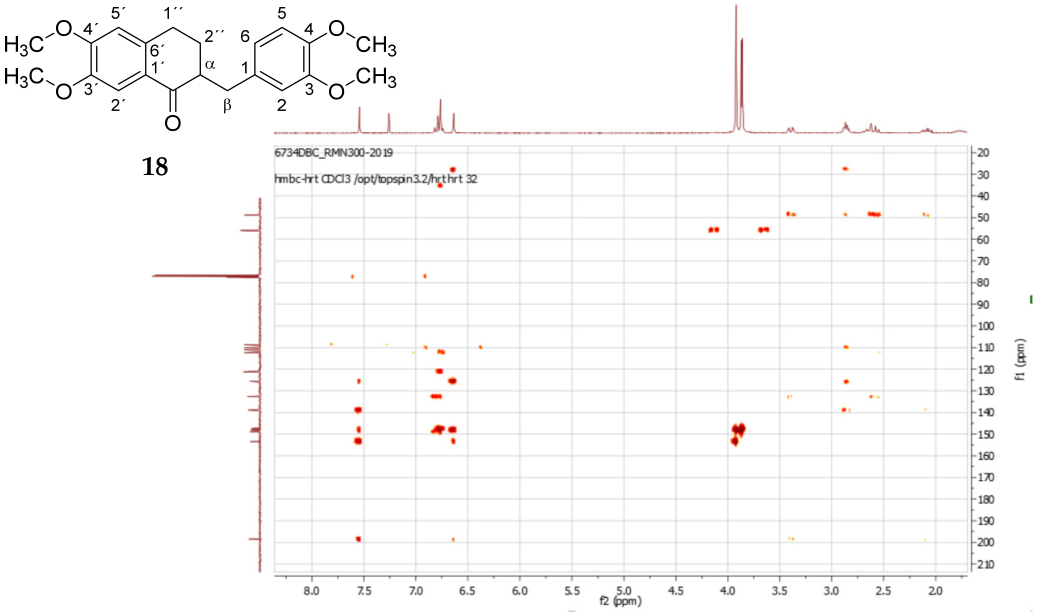

18

Figure S22. ^{1}H NMR and ^{13}C NMR spectra of compound **18**.

2-(3,4-dimethoxybenzyl)-6,7-dimethoxy-3,4-dihydronaphthalene-1(2*H*)-one) (18): purified by TLC (SiO_2, n-hexane/ ethyl acetate 7:3). Yield: 60% as a white solid; m.p. (n-hexane/ ethyl acetate 7:3) = 139.5 – 140.1 °C; IR (KBr, υ (cm^{-1})): 3083 (C_{sp2}-H); 2997, 2939, 2851, 2832 (C_{sp3}-H); 1659 (C=O); 1512, 1466, 1449, 1440 (aromatic C=C); 1181 (C-O); ^{1}H NMR (300 MHz, $CDCl_3$) δ (ppm): 7.54 (*s*, H-2′), 6.80 (*dd*, J = 7.6; 1.9 Hz, H-6), 6.78 (*d*, J = 7.6 Hz, H-6), 6.78 (*d*, J = 7.6 Hz, H-5), 6.74 (*d*, J = 1.9 Hz, H-2), 6.64 (*s*, H-5′), 3.92 (*s*, 3-OCH_3, 4-OCH_3), 3.87 (*s*, 4′-OCH_3), 3.86 (*s*, 3′-OCH_3), 3.39 (*m*, H-α), 2.80 (*t*, J = 4.8 Hz, H-1′′), 2.63 (*m*, H- βa), 2.57 (*m*, H-βb), 2.10 (*m*, H-2′′a), 1.77 (*m*, H-2′′b); ^{13}C NMR (300 MHz, $CDCl_3$) δ (ppm):198.1 (C=O), 153.2 (C-4′), 149.0 (C-4), 148.9 (C-3′), 147.4 (C-3), 134.1 (C-1′), 130.1 (C-1), 122.7 (C-6′), 120.1 (C-2), 111.8 (C-6), 111.3 (C-5), 110.1 (C-2′), 110.0 (C-5′), 56.1 (4′-OCH_3, 4-OCH_3), 56.0 (3-OCH_3), 55.9 (3′-OCH_3), 40.3 (C-β), 30.2 (C-α).

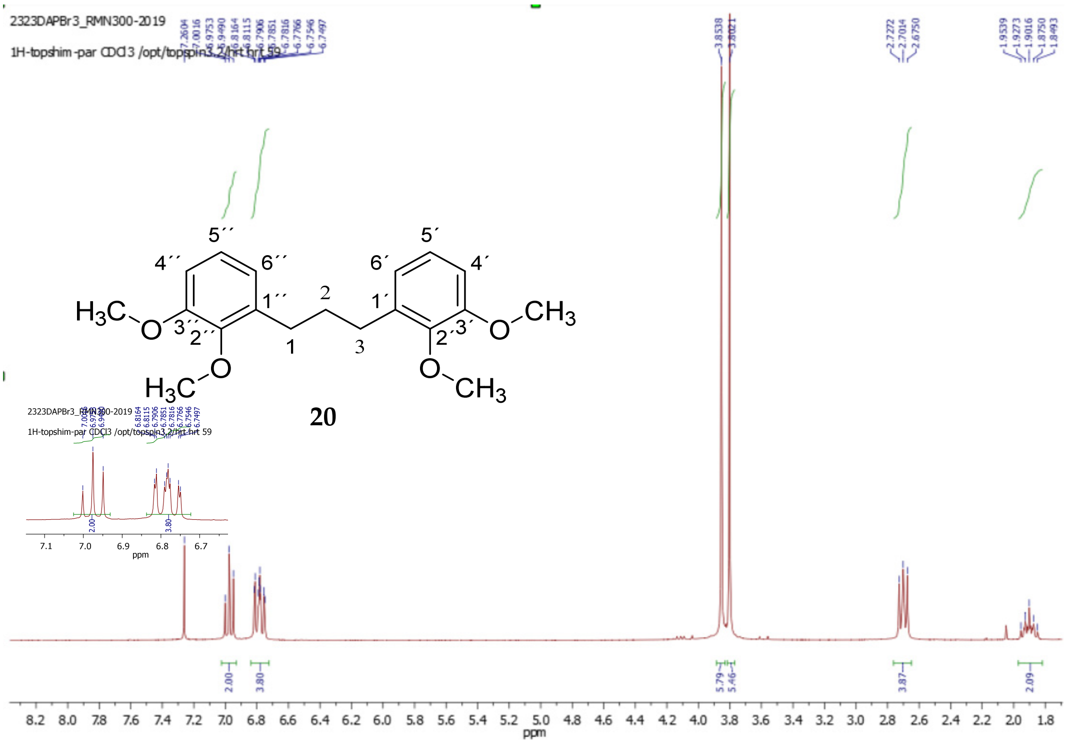

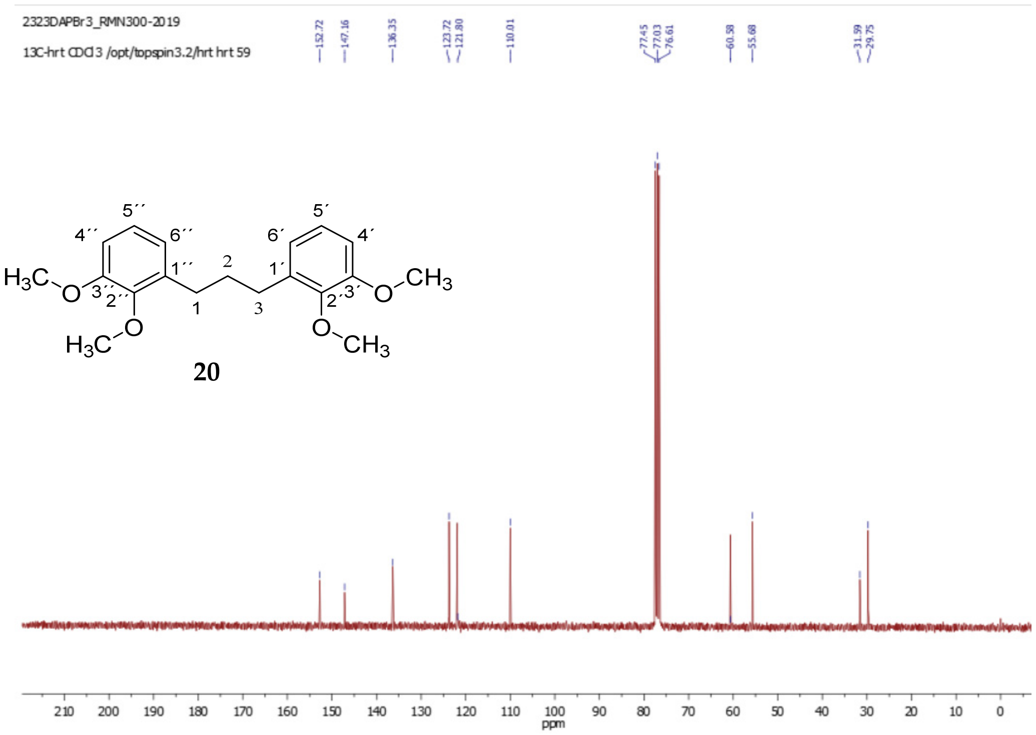

Figure S23. ^{1}H NMR and ^{13}C NMR spectra of compound **20**.

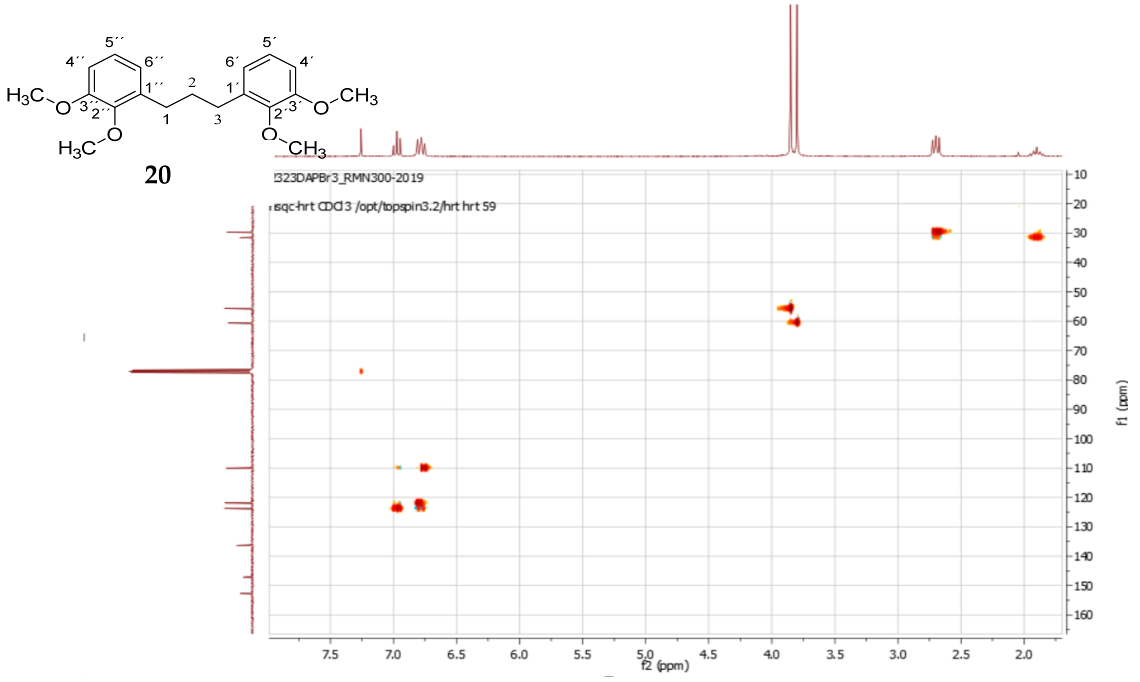

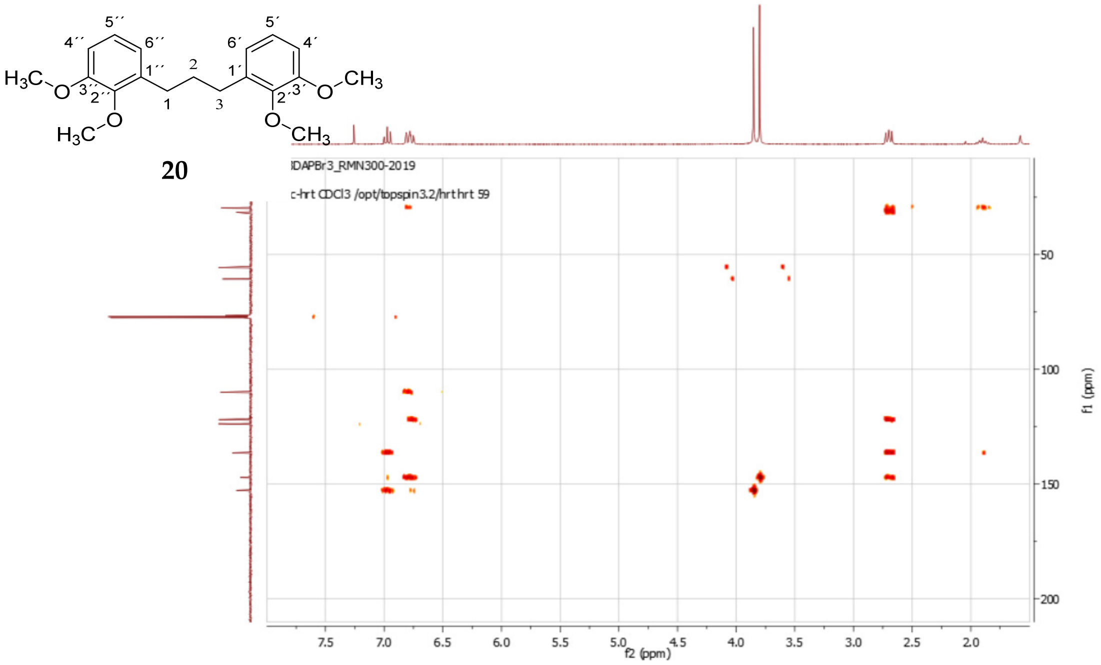

Figure S24. ^{1}H NMR and ^{13}C NMR spectra of compound **20**.

1,3-bis(2,3-dimethoxyphenyl)propane (20): purified by TLC (SiO_2, n-hexane/ ethyl acetate 7:3). Yield: 66% as a colorless oil; IR (KBr, υ (cm^{-1})): 2934, 2860, 2833 (C_{sp3}-H); 1598, 1584, 1474, 1429 (aromatic C=C); 1170 (C-O); ^{1}H NMR (300 MHz, $CDCl_3$) δ (ppm): 6.98 (*t*, J = 8.1 Hz, H-5′′, H-5′), 6.80 (*dd*, J = 8.1; 1.7 Hz, H-6′′, H-6′), 6.76 (*dd*, J = 8.1; 1.7 Hz, H-4′′, H-4′), 3.85 (*s*, 3′′-OCH_3, 3′-OCH_3), 3.80 (*s*, 2′′-OCH_3, 2′-OCH_3), 2.70 (*t*, J = 7.8 Hz, H-1, H-3), 1.90 (*m*, H-2); ^{13}C NMR (300 MHz, $CDCl_3$) δ (ppm): 152.7 (C-3′′, C-3′), 147.2 (C-2′′, C-2′), 123.7 (C-5′′, C-5′), 121.8 (C-6′′, C-6′), 110.0 (C-4′′, C-4′), 61.0 (2′′-OCH_3, 2′-OCH_3), 55.7 (3′′-OCH_3, 3′-OCH_3), 31.6 (C-2), 29.8 (C-1, C-3).

NMR and HRMS spectra of compounds 2, 5, 11, 12, 17, and 21-26.

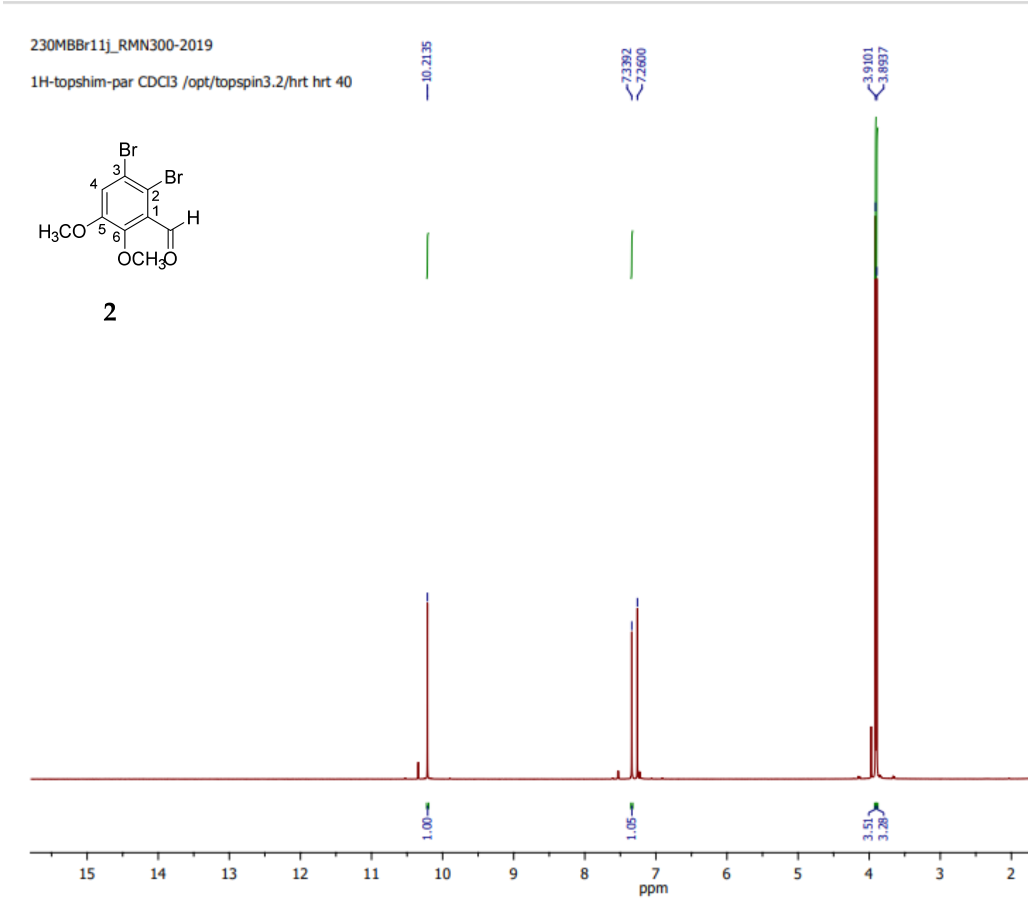

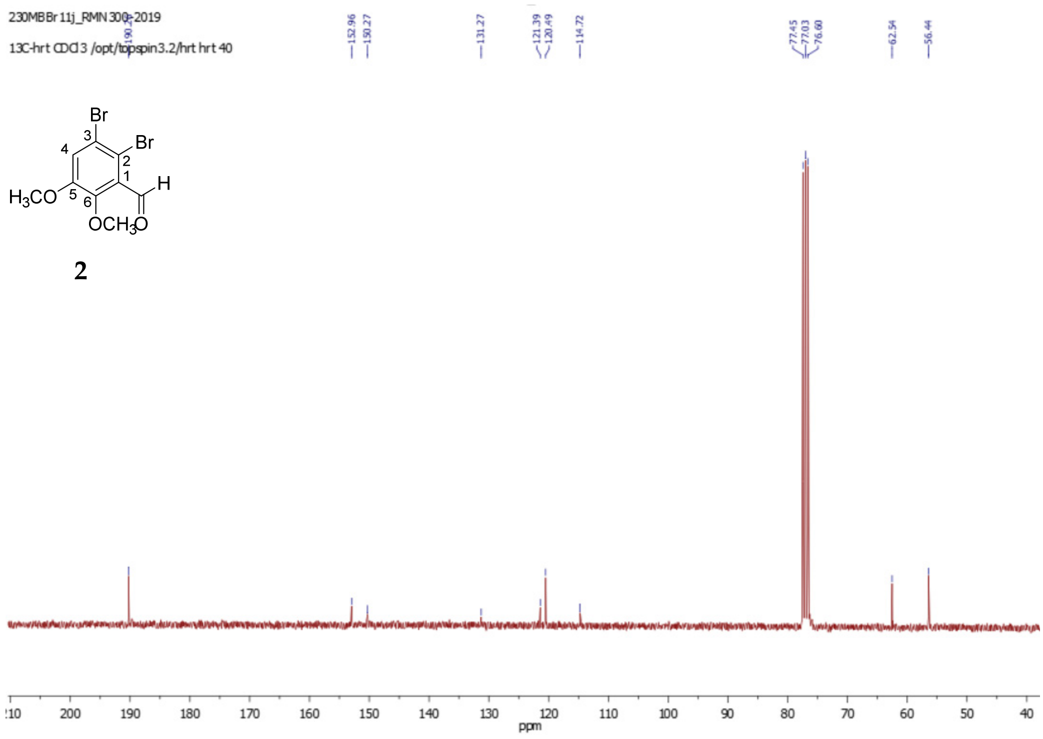

Figure S25. ^{1}H NMR and ^{13}C NMR spectra of compound **2**.

2

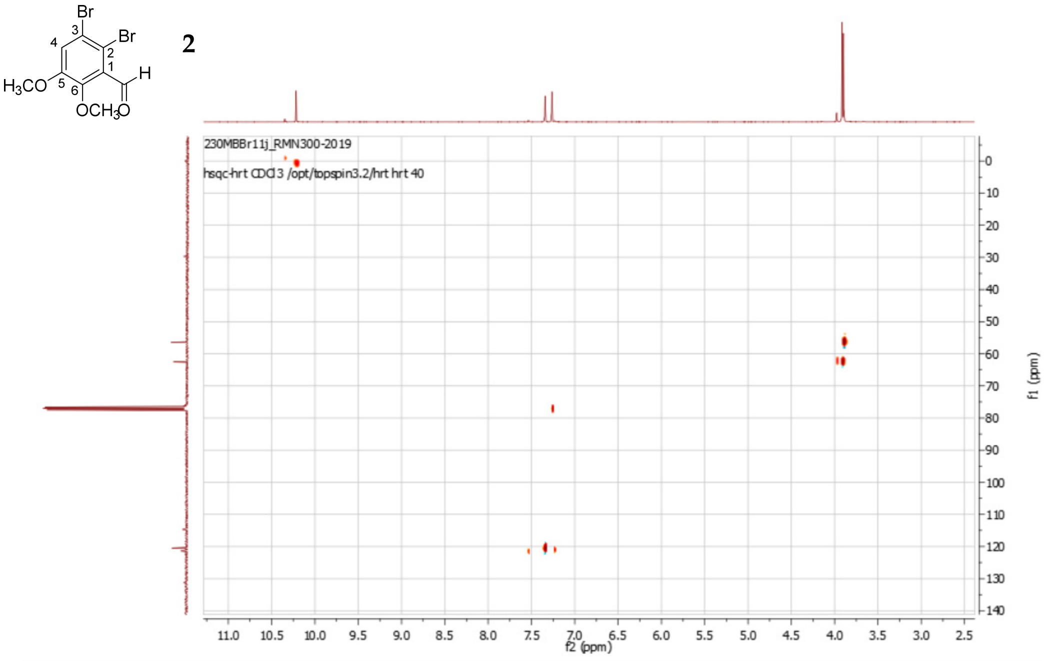

2

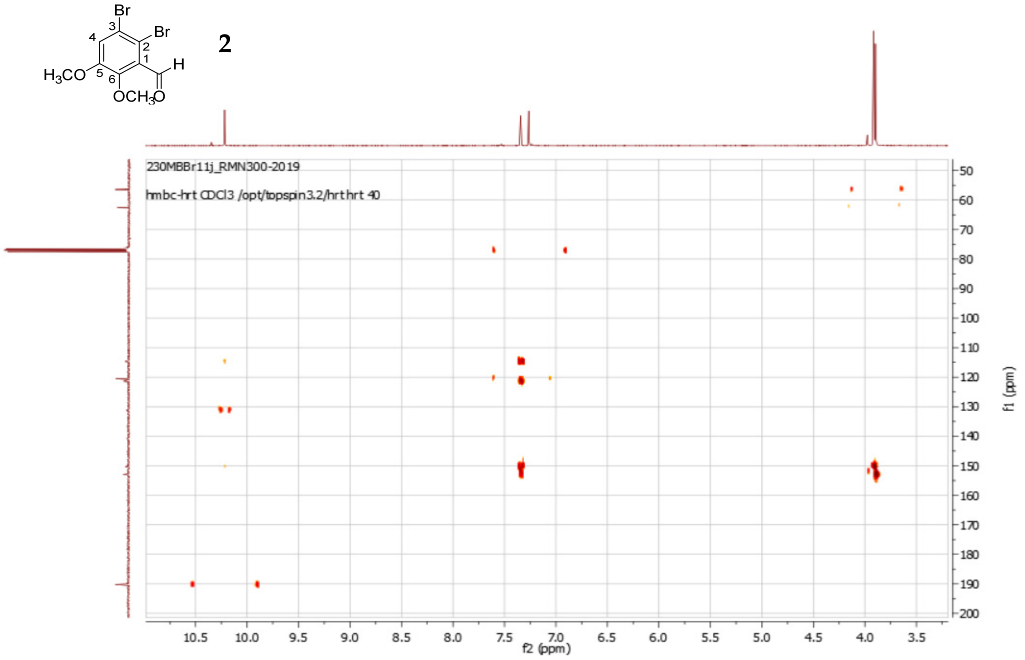

Figure S26. HSQC and HMBC spectra of compound **2**.

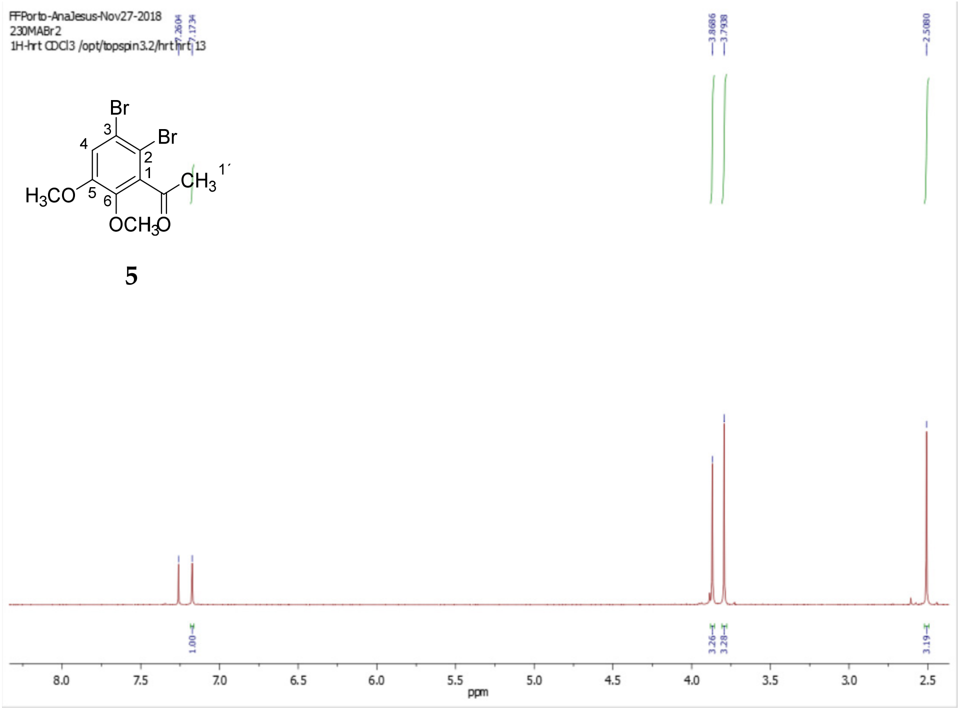

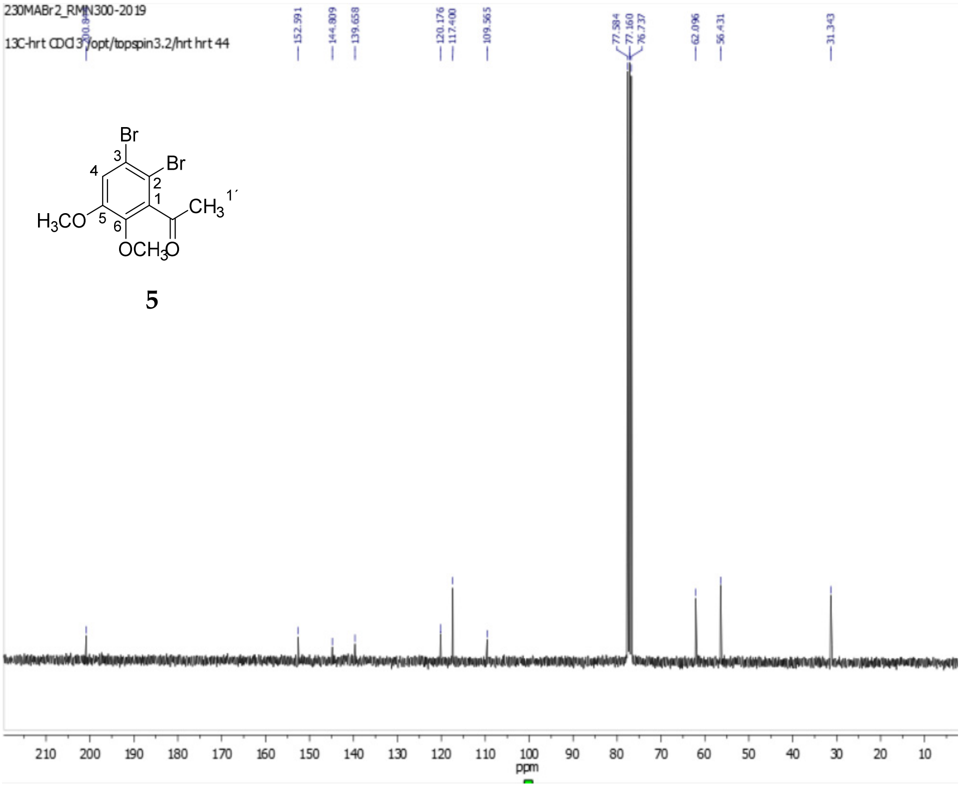

Figure S27. ^{1}H NMR and ^{13}C NMR spectra of compound **5**.

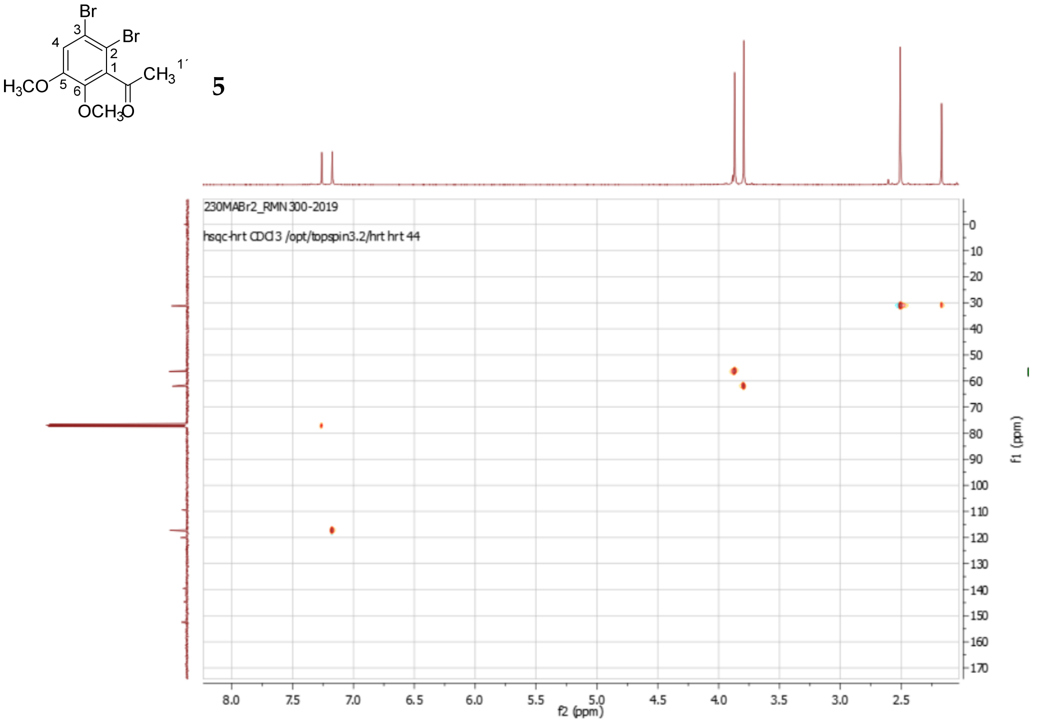

5

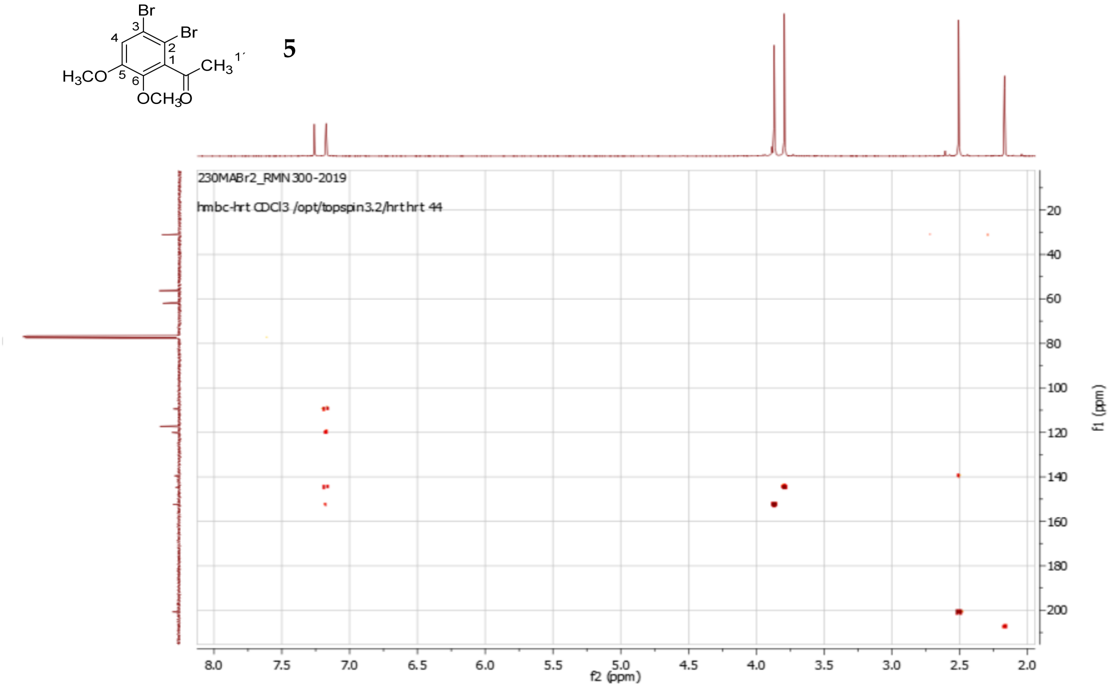

5

Figure S28. HSQC and HMBC spectra of compound **5**.

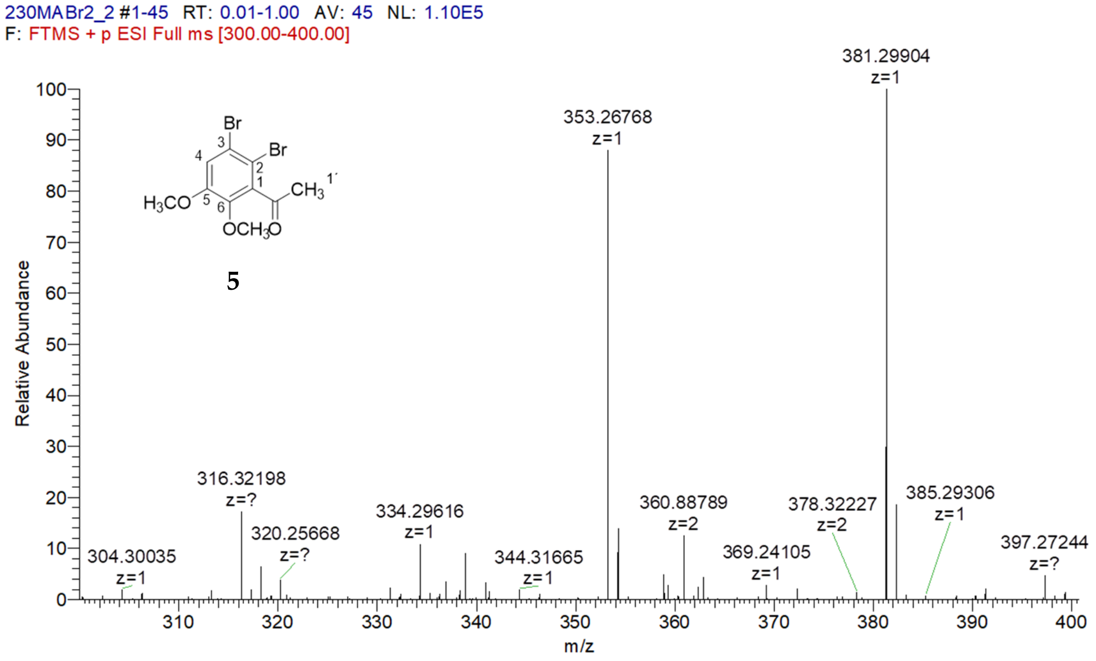

m/z calculated (ppm)	Formula	m/z found (ppm)	Error (ppm)
382.89762	$C_{10}H_{10}Br_2O_3Na_2$	381.29904	-1.59858

Figure S29. HRMS spectrum of compound **5**.

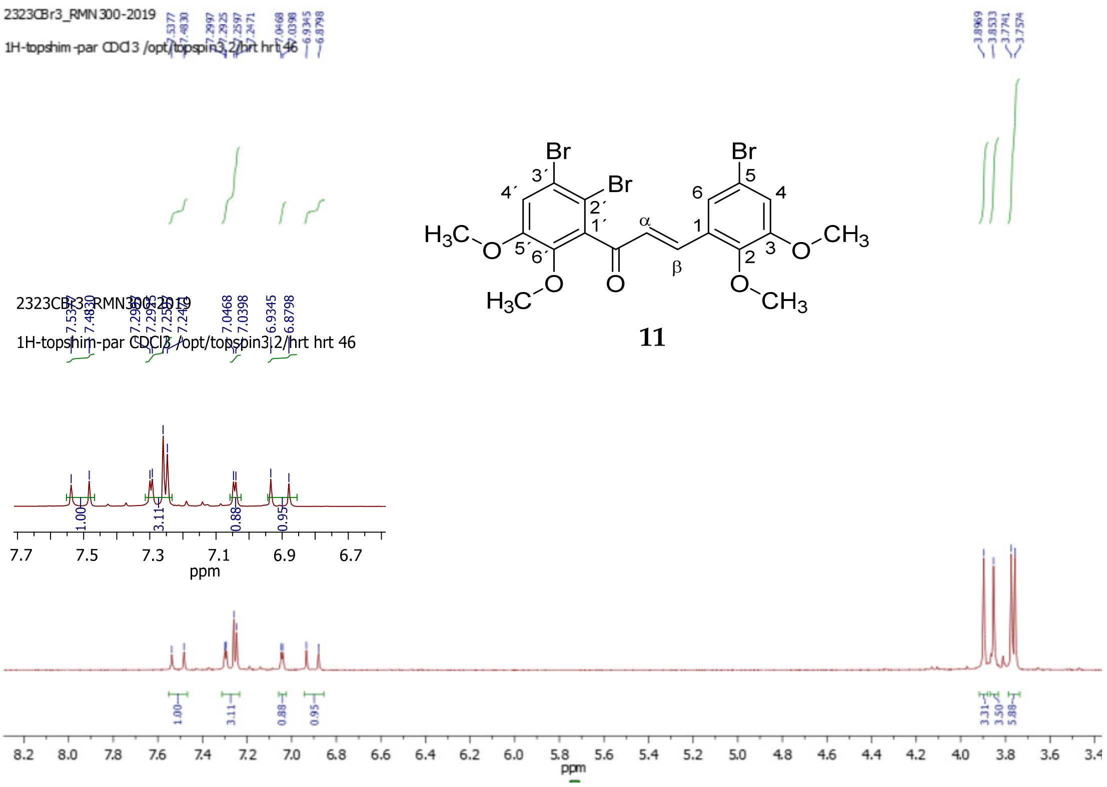

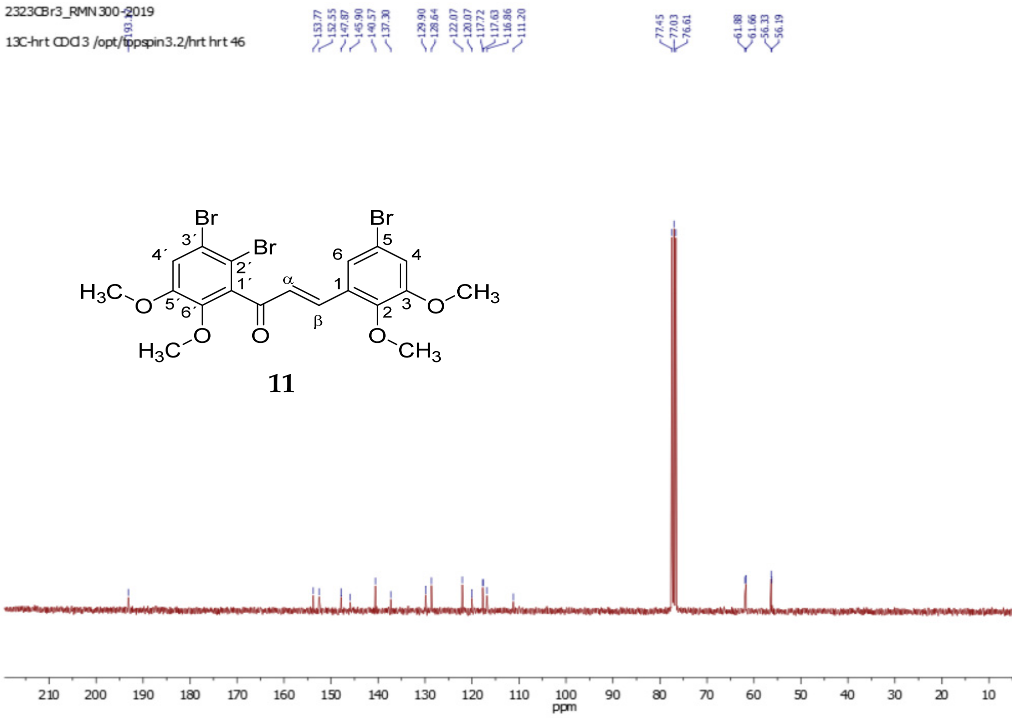

Figure S30. ^{1}H NMR and ^{13}C NMR spectra of compound **11**.

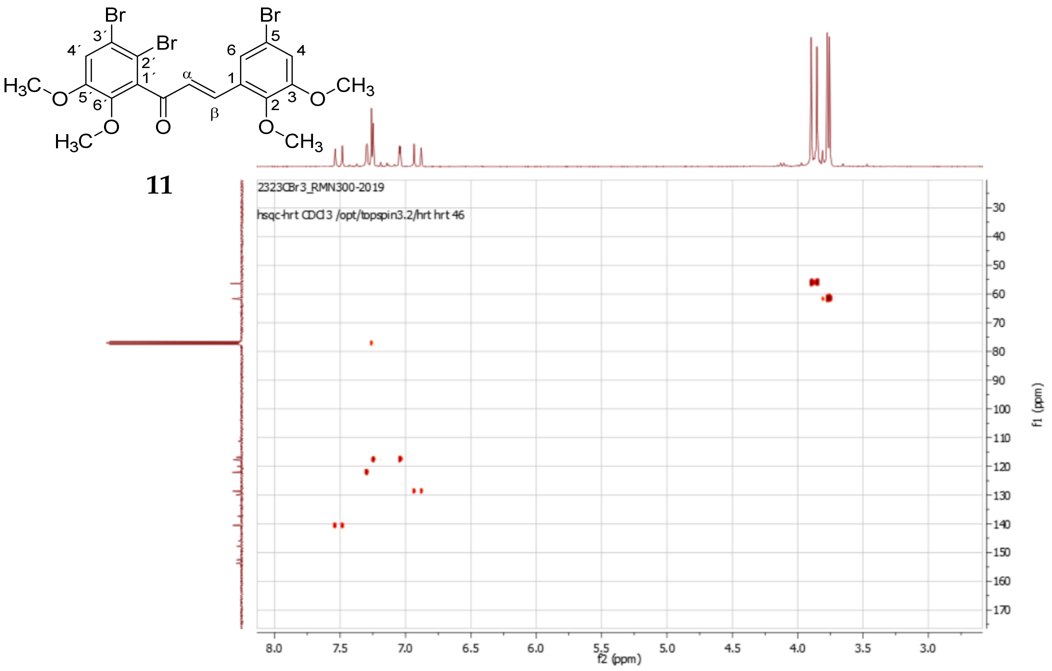

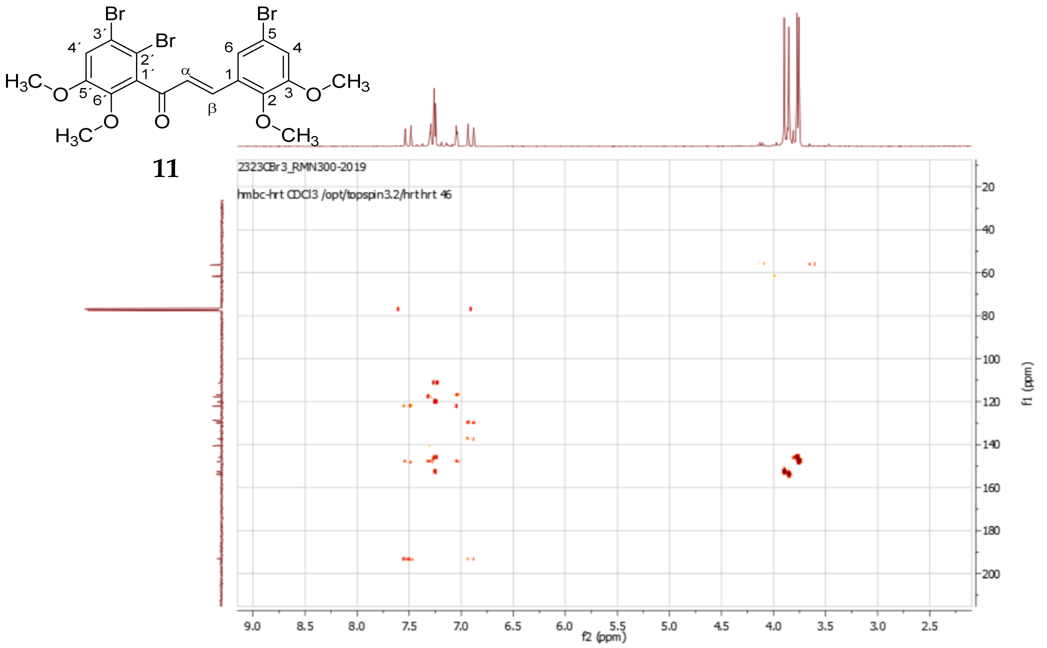

Figure S31. HSQC and HMBC spectra of compound **11**.

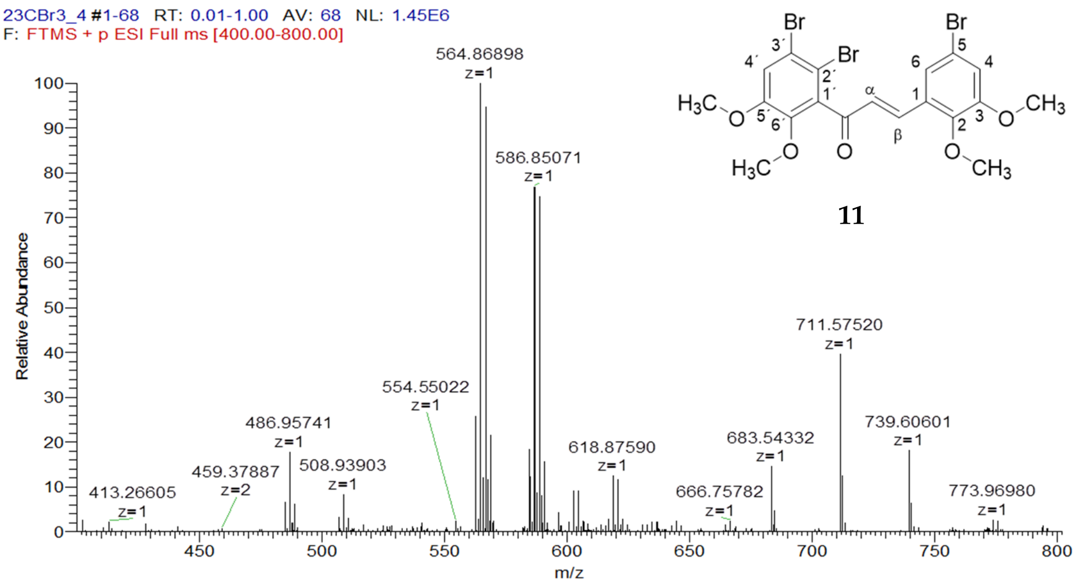

11

m/z calculated (ppm)	Formula	m/z found (ppm)	Error (ppm)
565.05200	$C_{19}H_{17}Br_3O_5$	564.86898	-0.18302

Figure S32. HRMS spectrum of compound **11**.

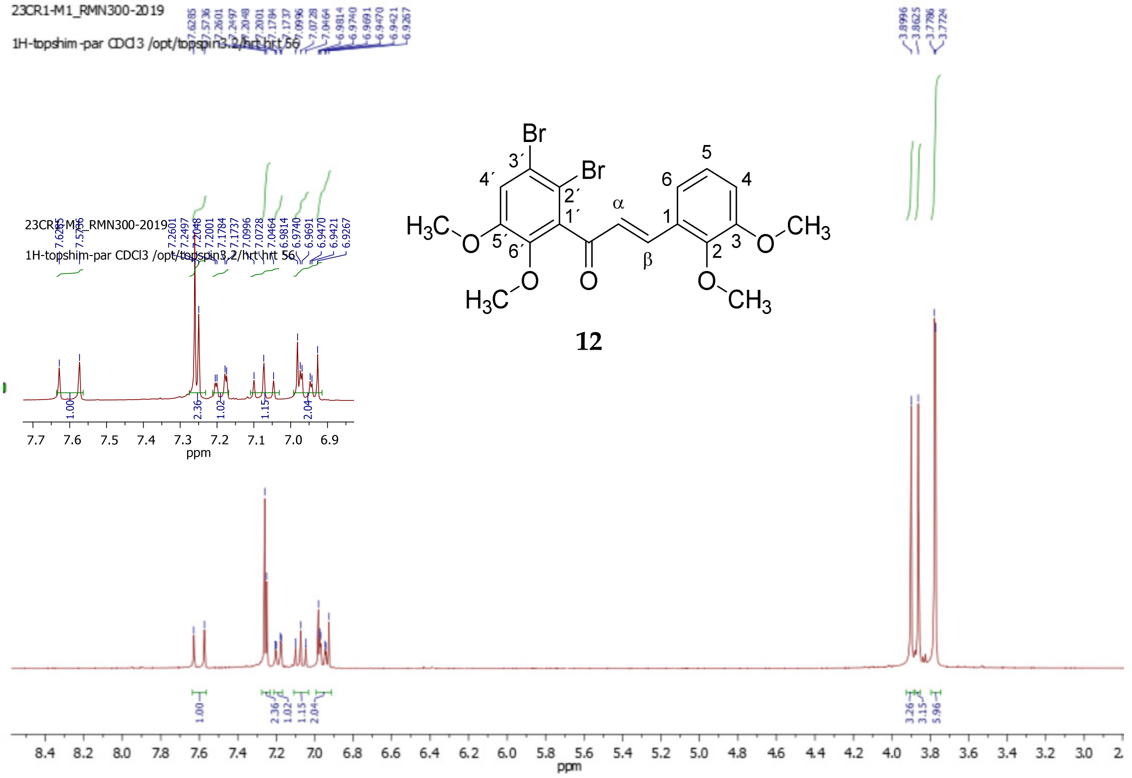

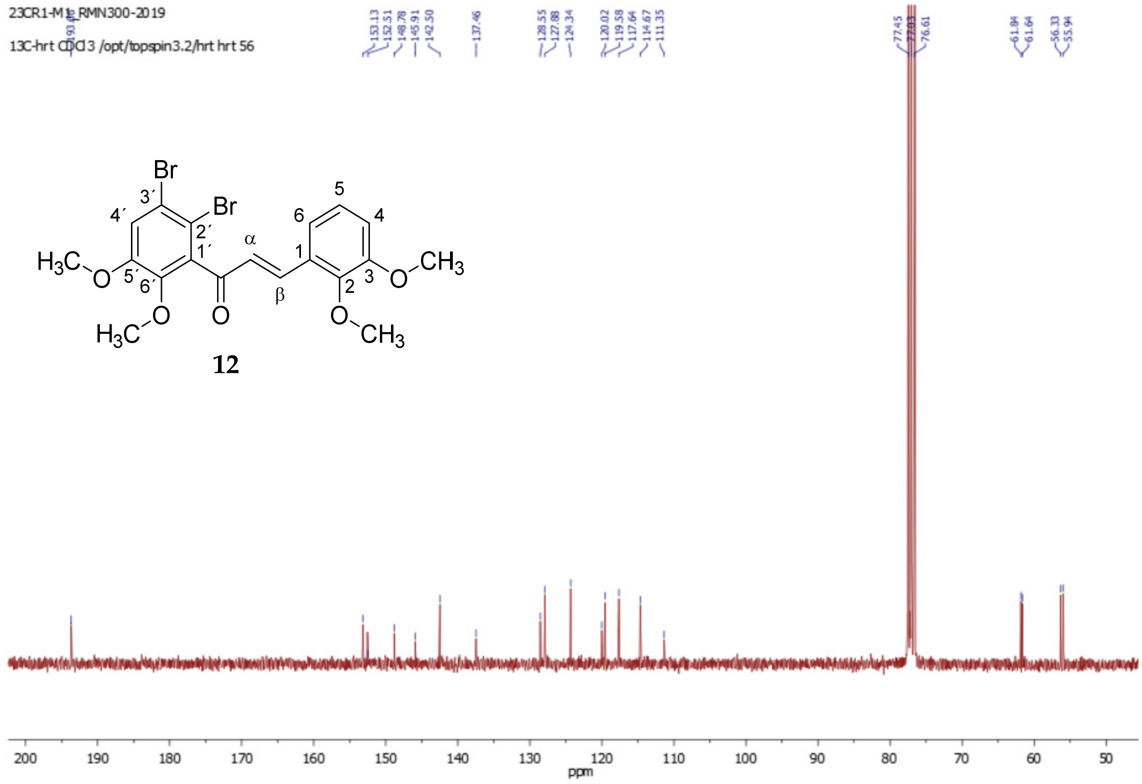

Figure S33. ^{1}H NMR and ^{13}C NMR spectra of compound **12**.

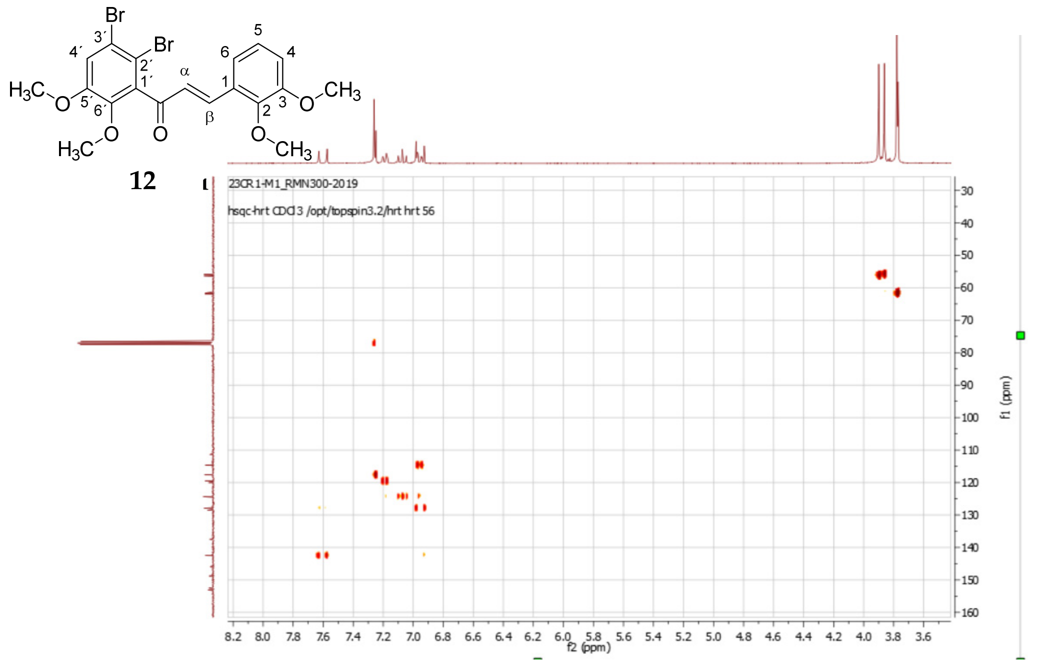

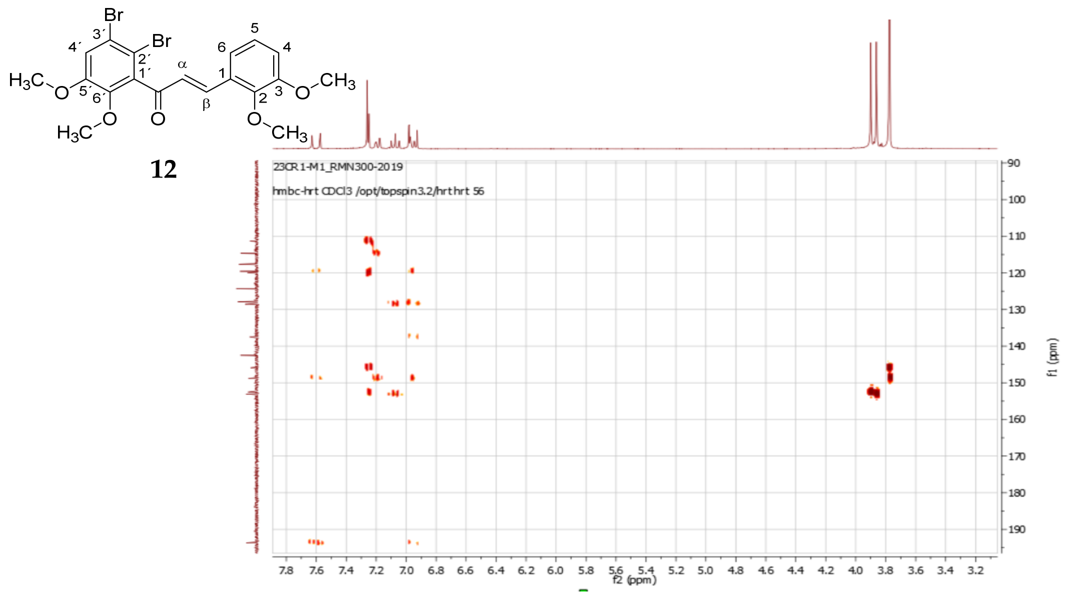

Figure S34. HSQC and HMBC spectra of compound **12**.

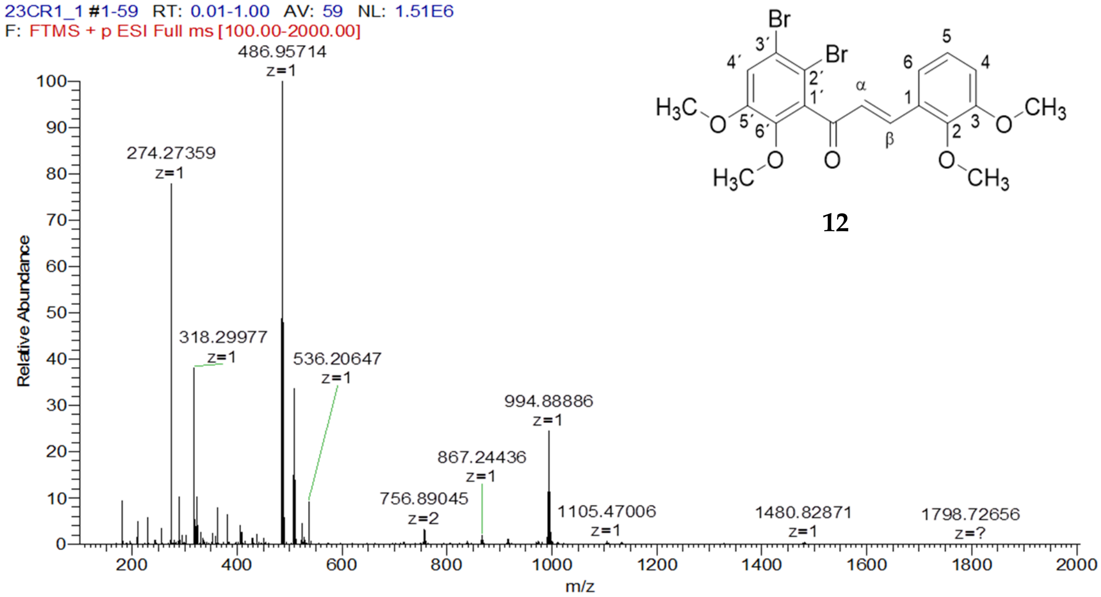

12

m/z calculated (ppm)	Formula	m/z found (ppm)	Error (ppm)
486.95005	$C_{19}H_{18}Br_2O_5$	486.95714	0.00709

Figure S35. HRMS spectrum of compound **12**.

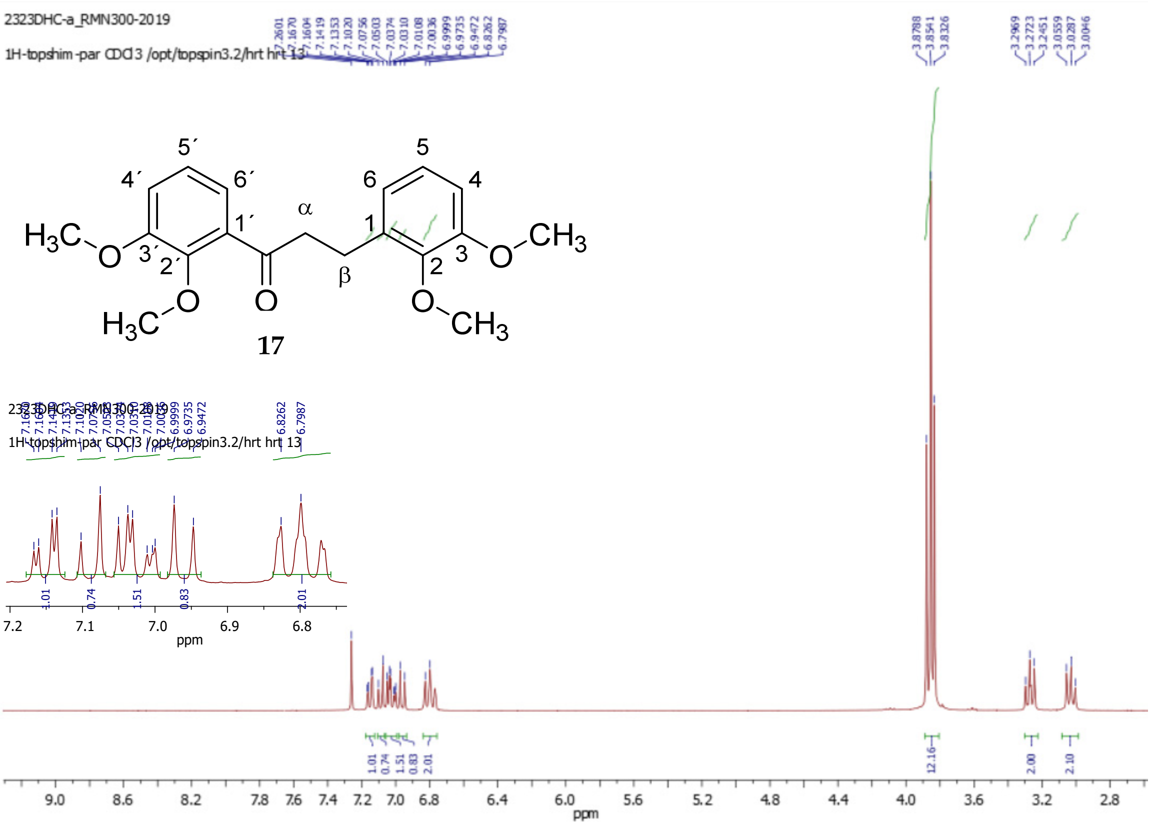

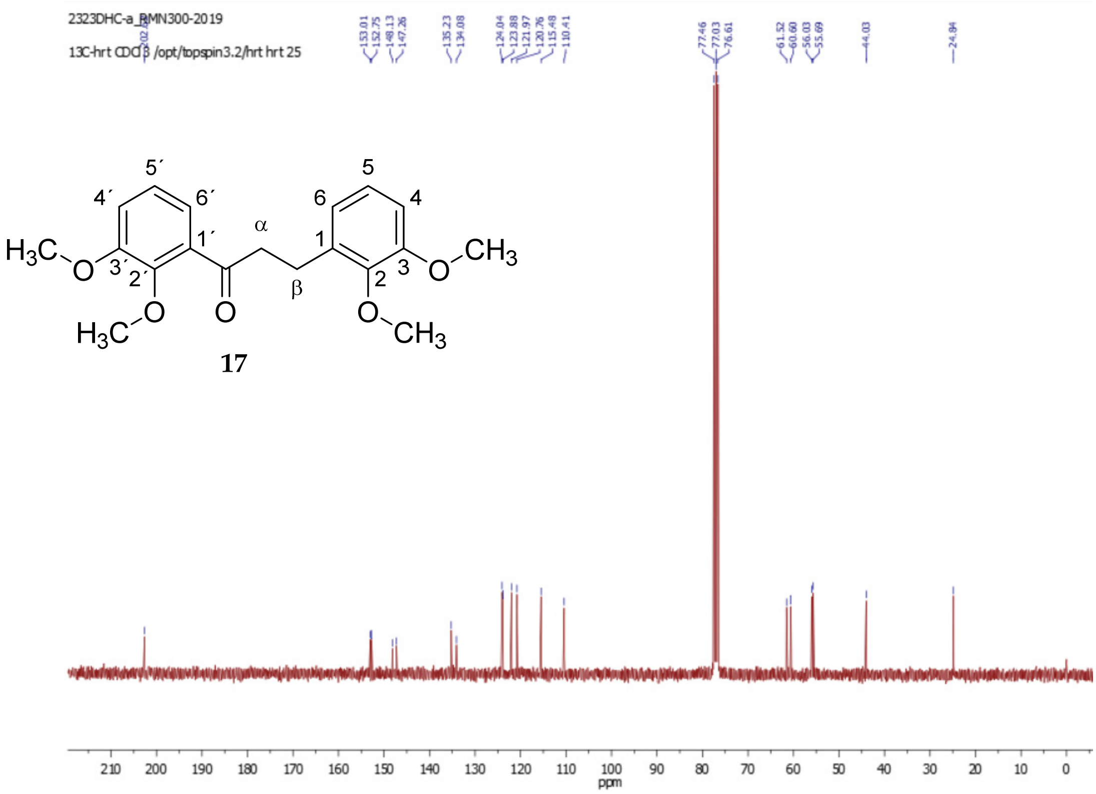

Figure S36. ^{1}H NMR and ^{13}C NMR spectra of compound **17**.

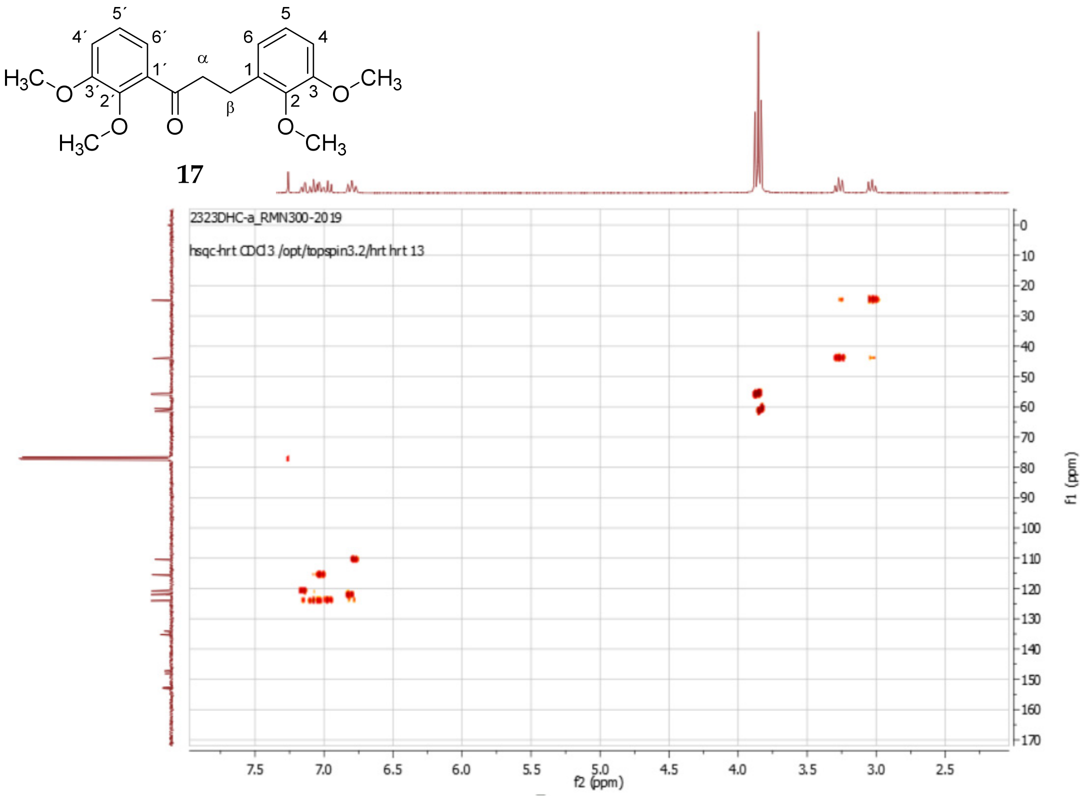

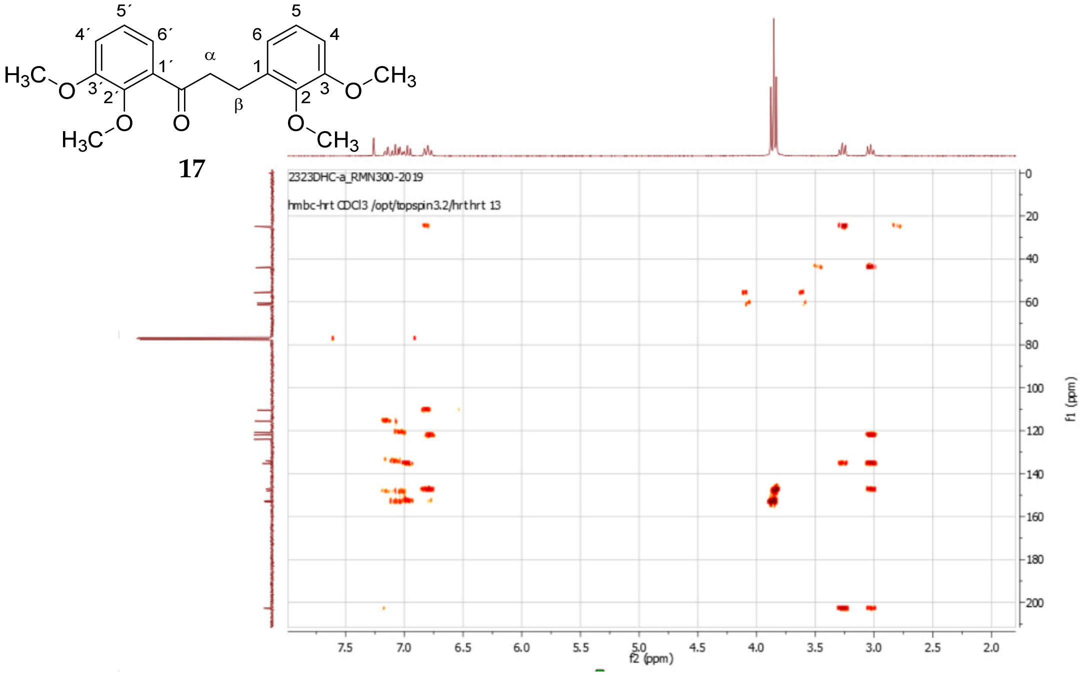

Figure S37. HSQC and HMBC spectra of compound **17**.

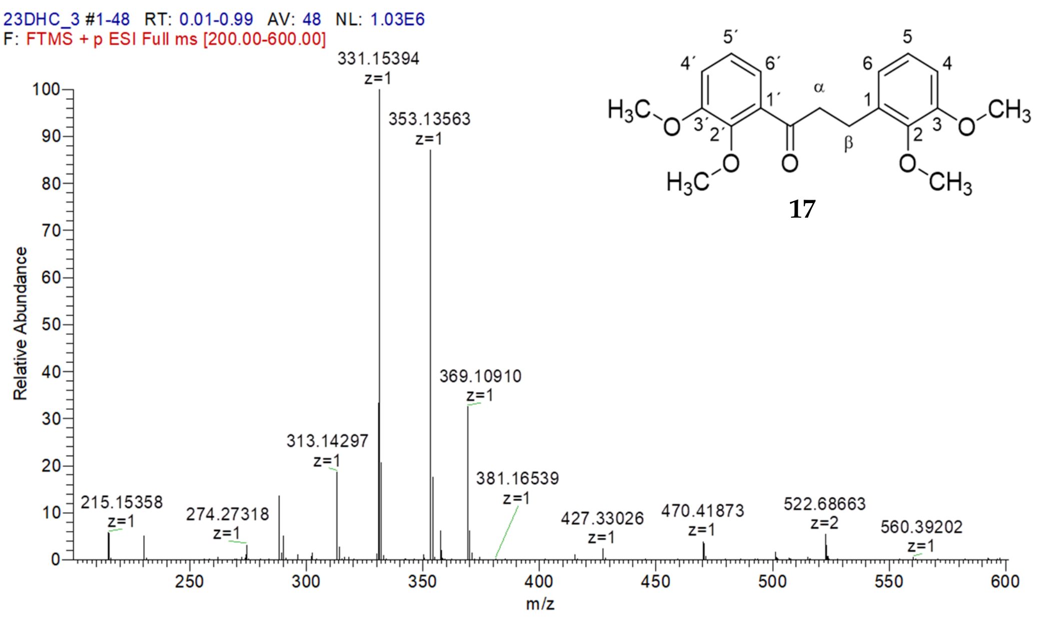

m/z calculated (ppm)	Formula	m/z found (ppm)	Error (ppm)
331.14672	$C_{19}H_{22}O_5$	331.15394	0.00722

Figure S38. HRMS spectrum of compound **17**.

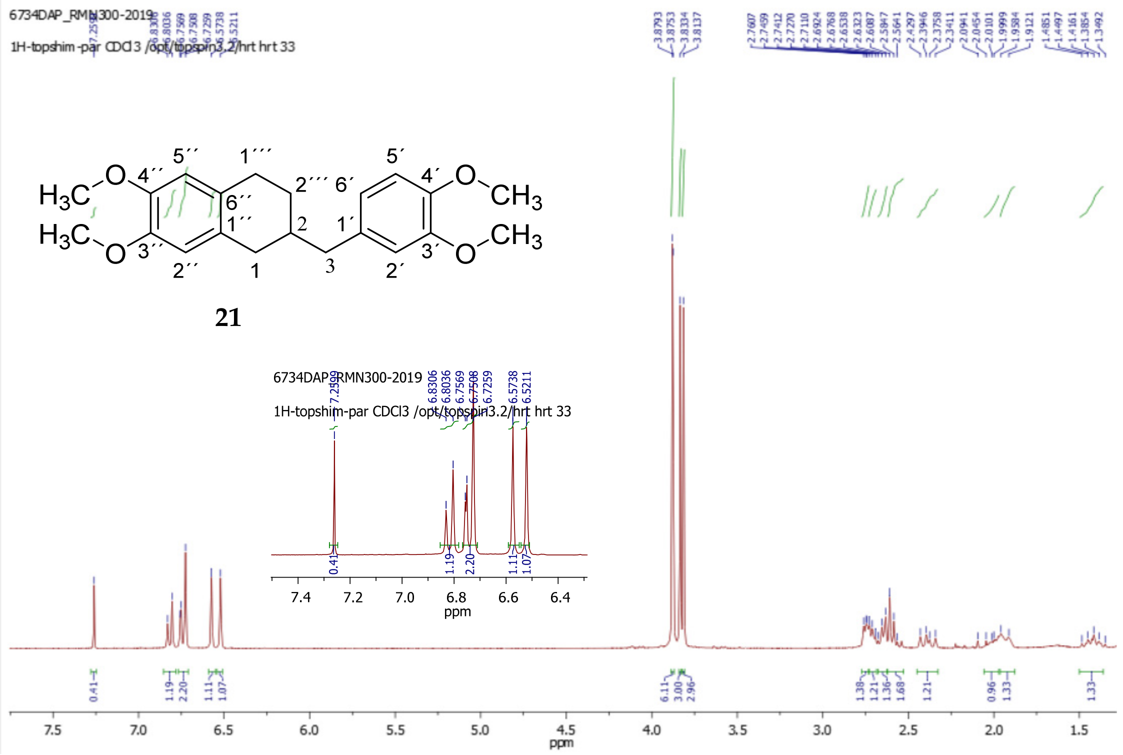

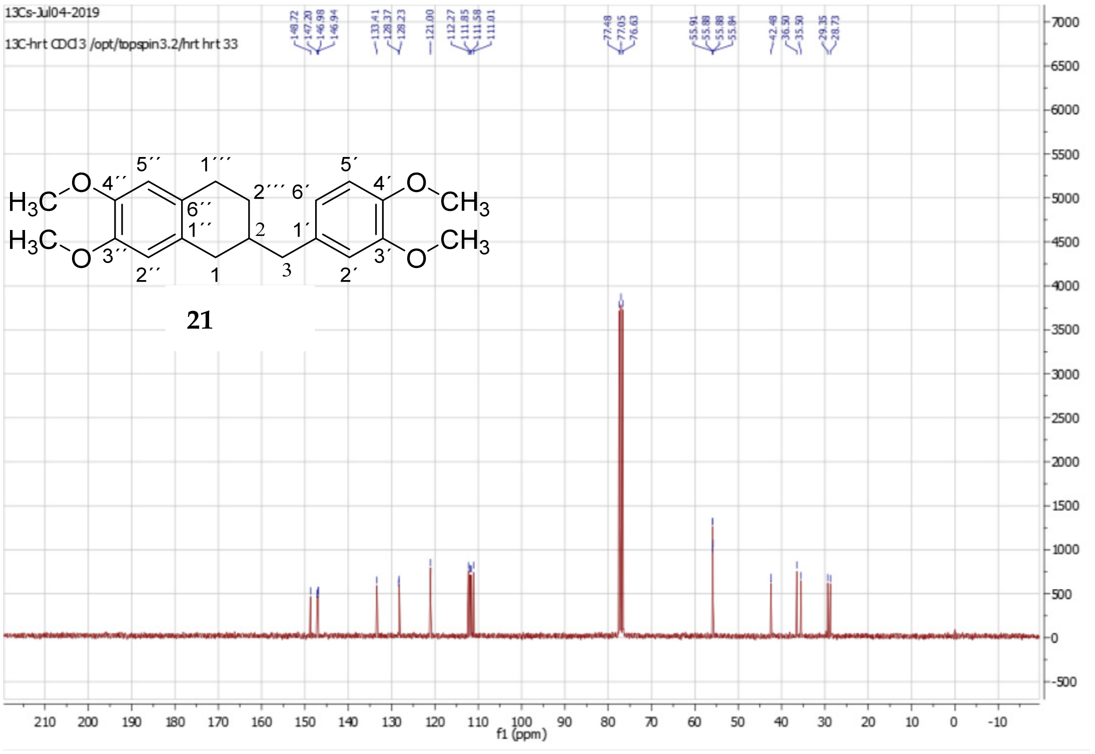

Figure S39. ^{1}H NMR and ^{13}C NMR spectra of compound **21**.

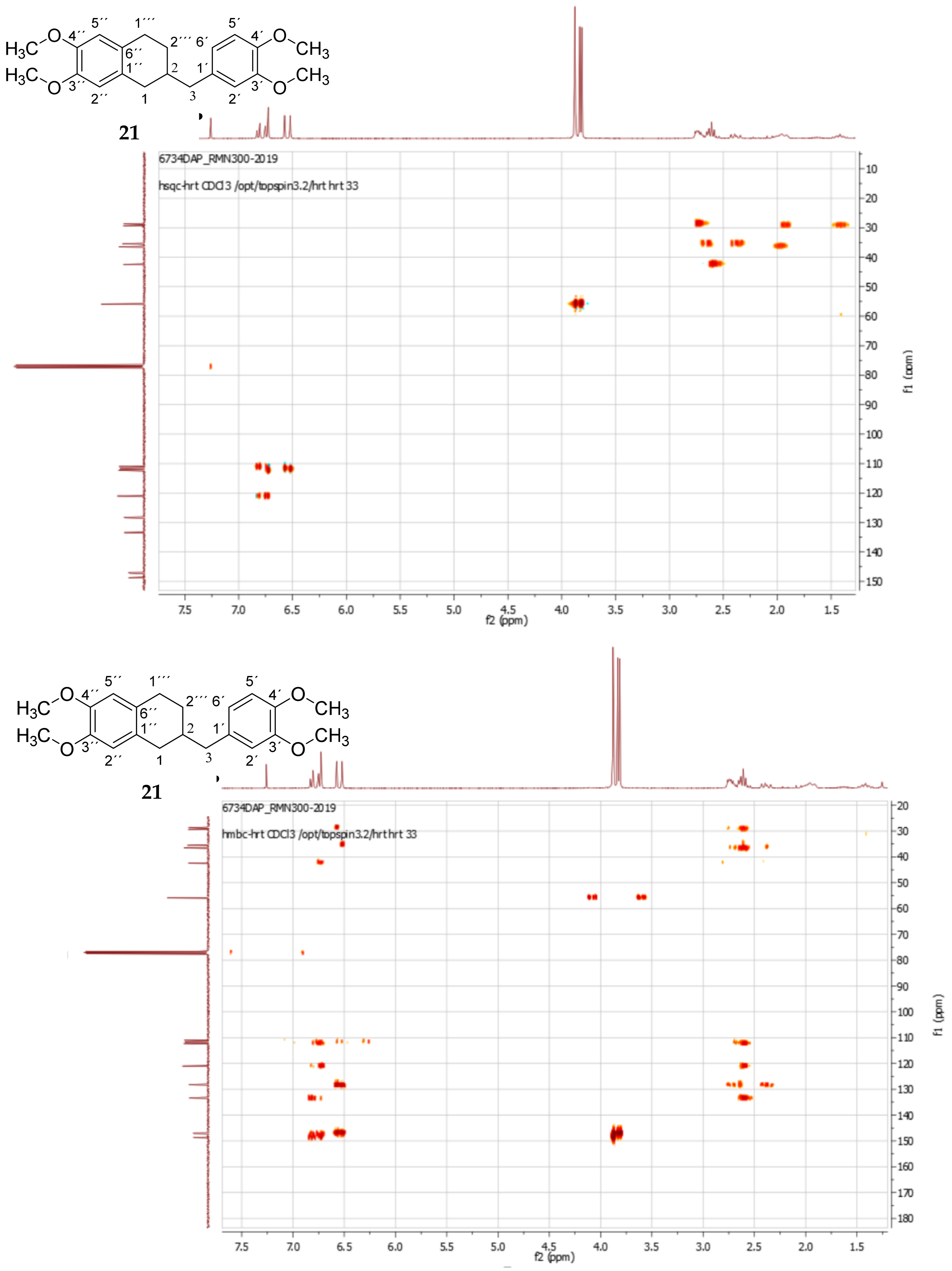

Figure S40. HSQC and HMBC spectra of compound **21**.

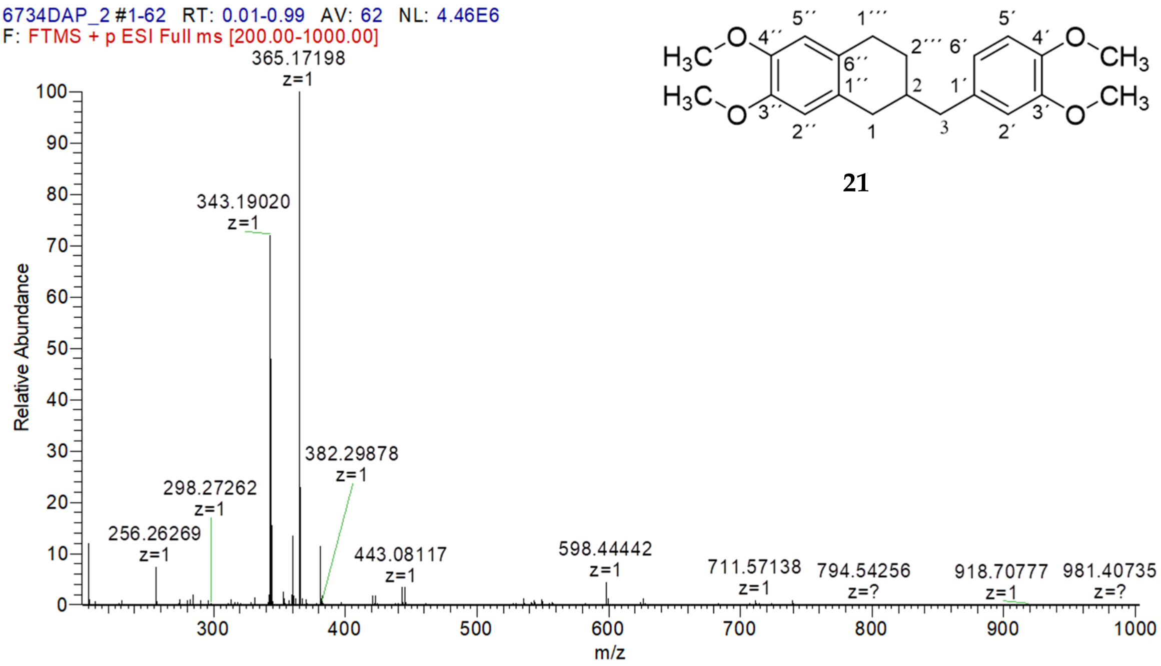

m/z calculated (ppm)	Formula	m/z found (ppm)	Error (ppm)
365.17288	$C_{21}H_{26}O_4Na$	365.17198	-0.0009

Figure S41. HRMS spectrum of compound **21**.

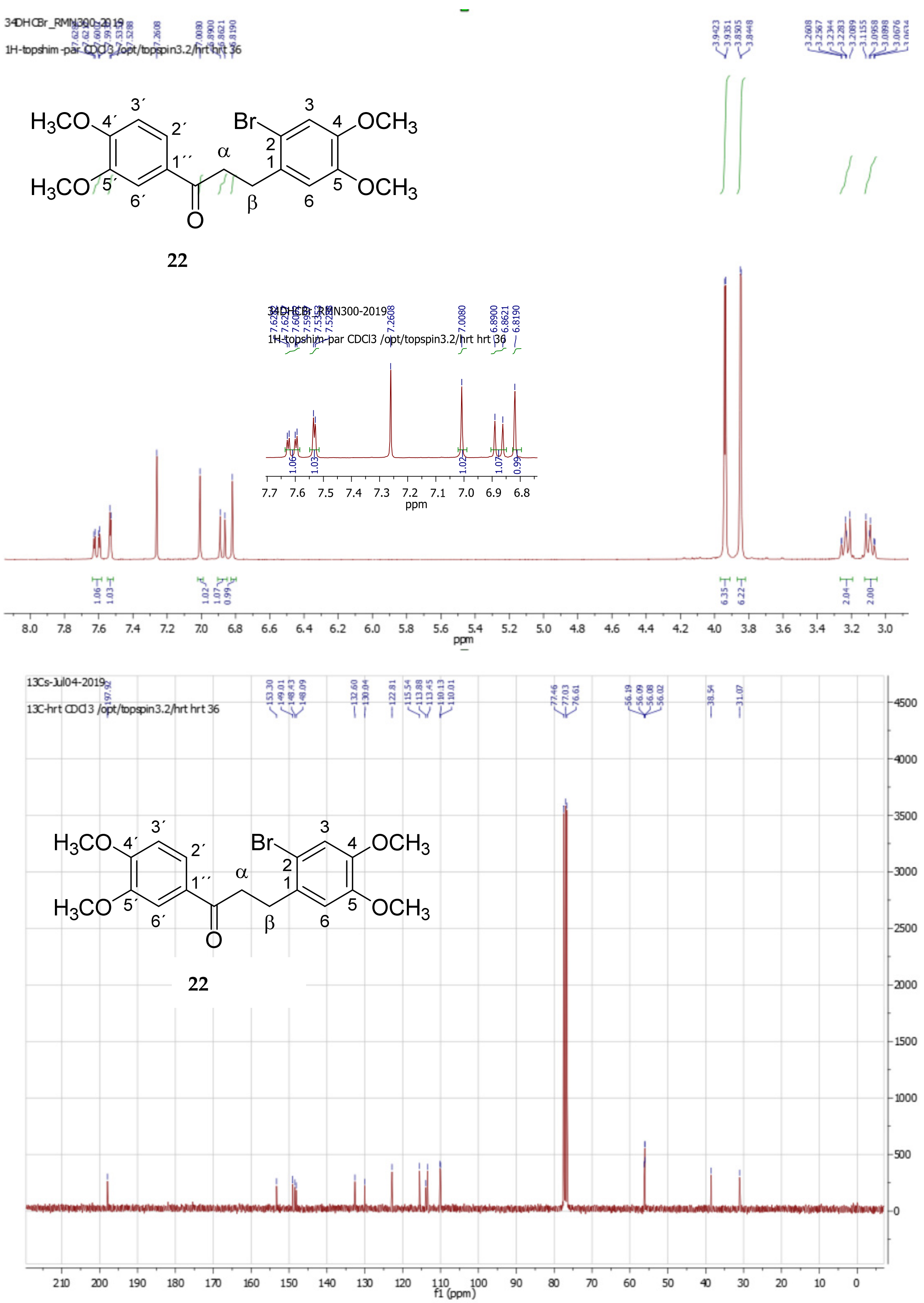

Figure S42. ^{1}H NMR and ^{13}C NMR spectra of compound **22**.

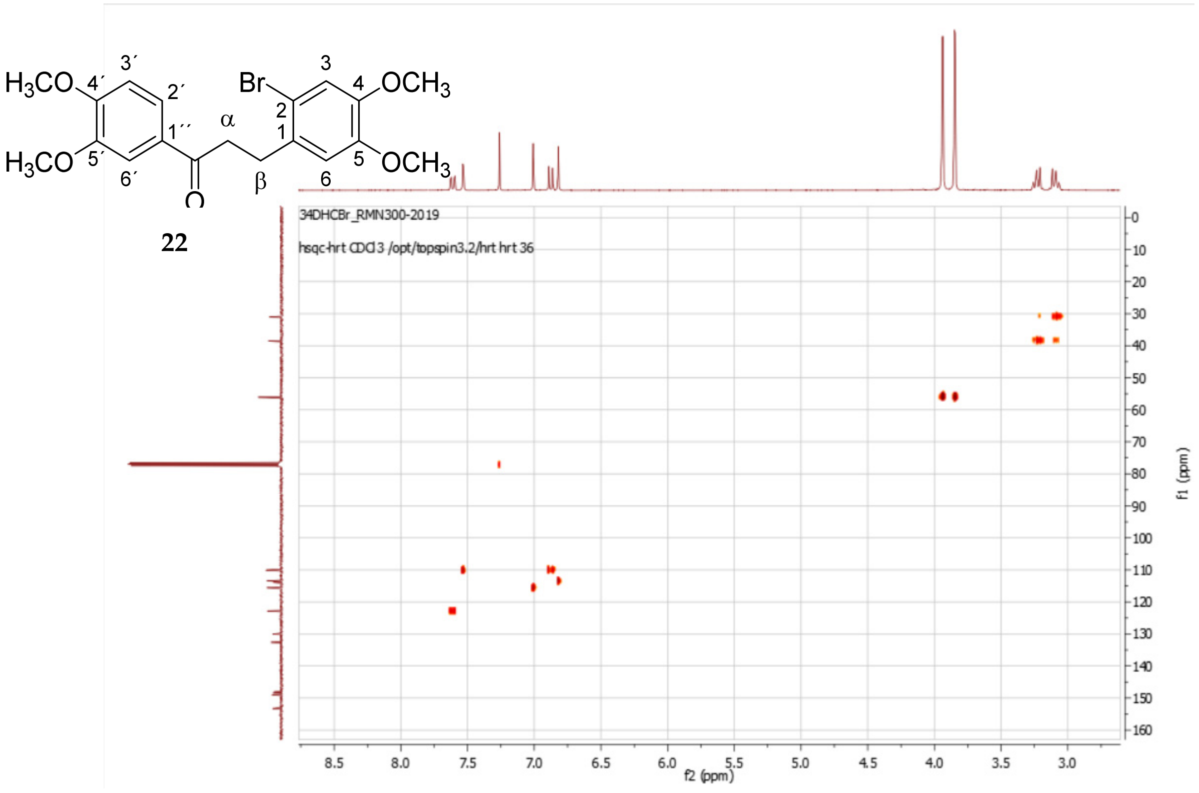

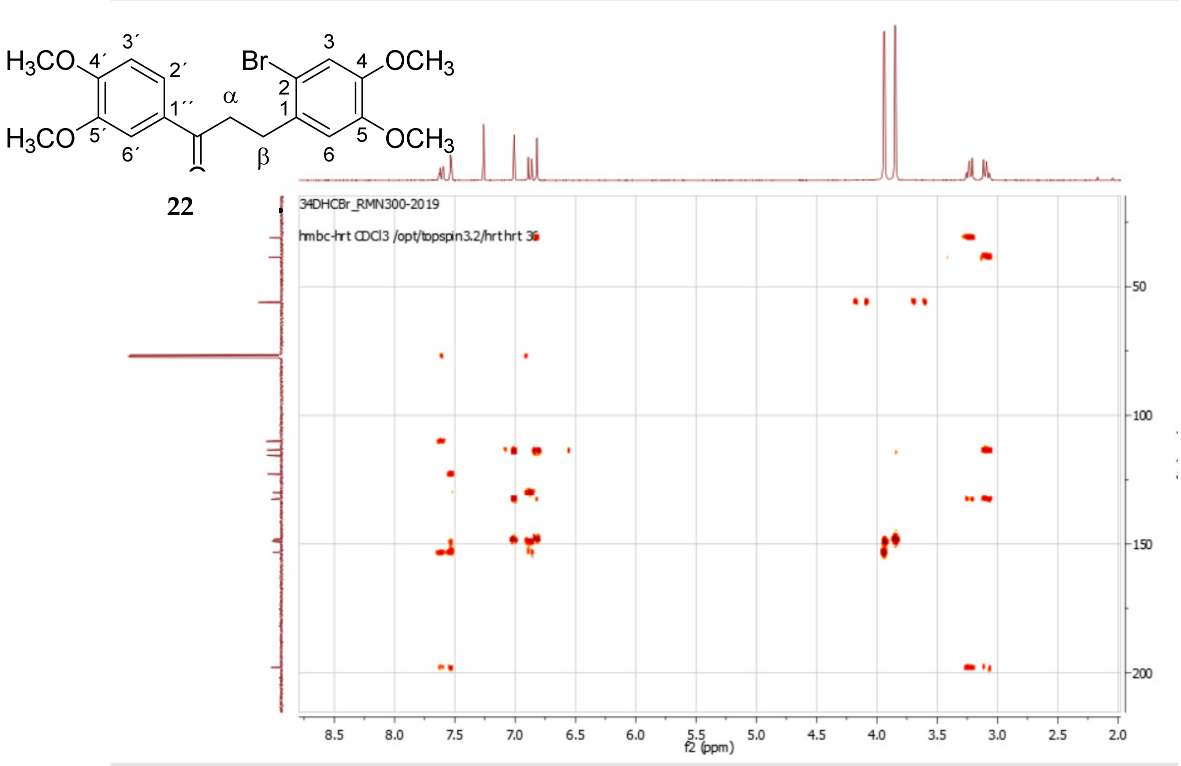

Figure S43. HSQC and HMBC spectra of compound **22**.

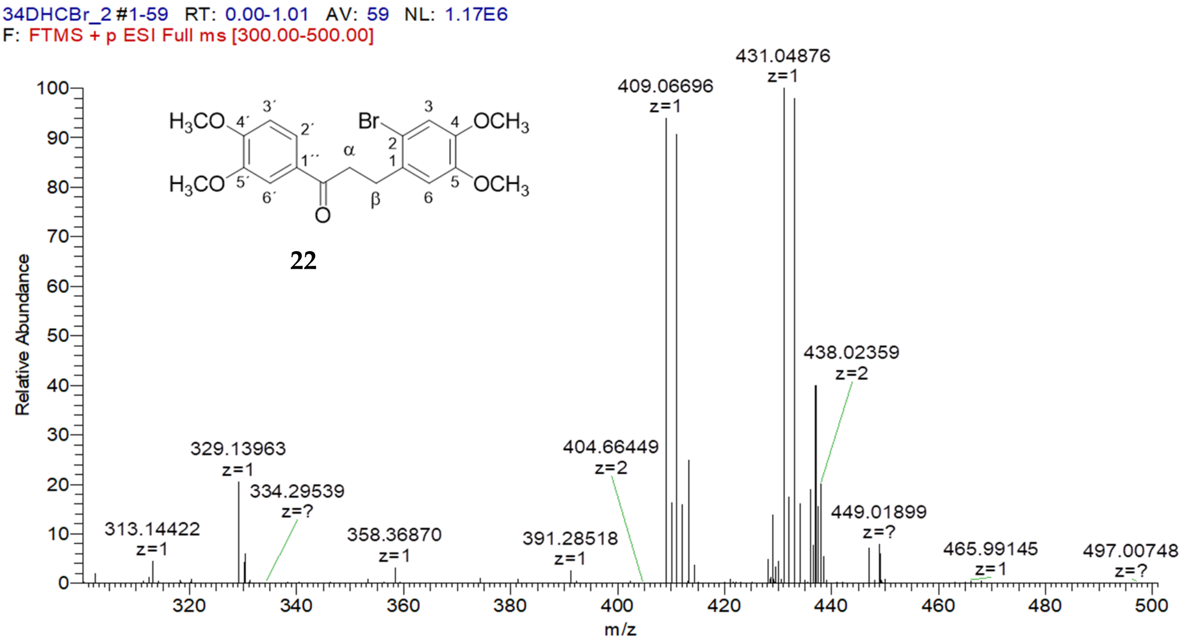

m/z calculated (ppm)	Formula	m/z found (ppm)	Error (ppm)
432.26577	$C_{19}H_{21}BrO_5Na$	431.04876	-1.21701

Figure S44. HRMS spectrum of compound **22**.

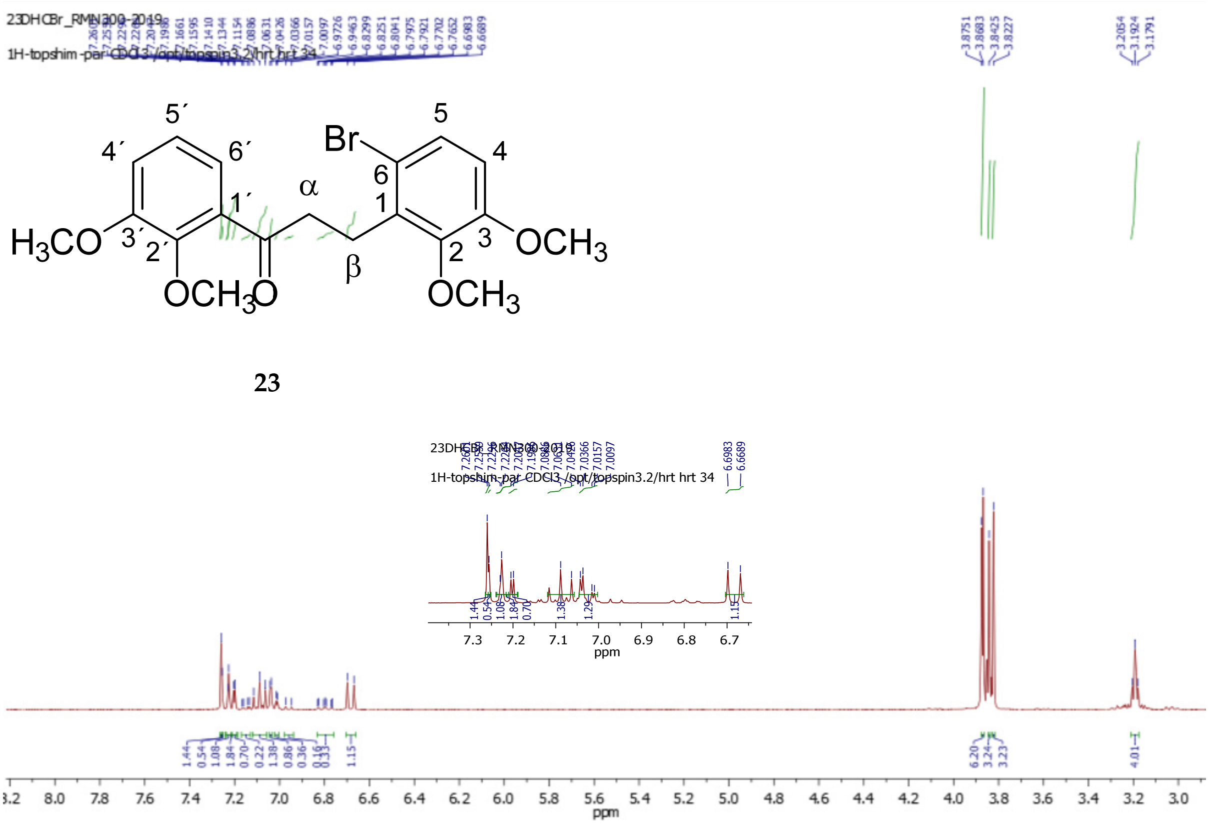

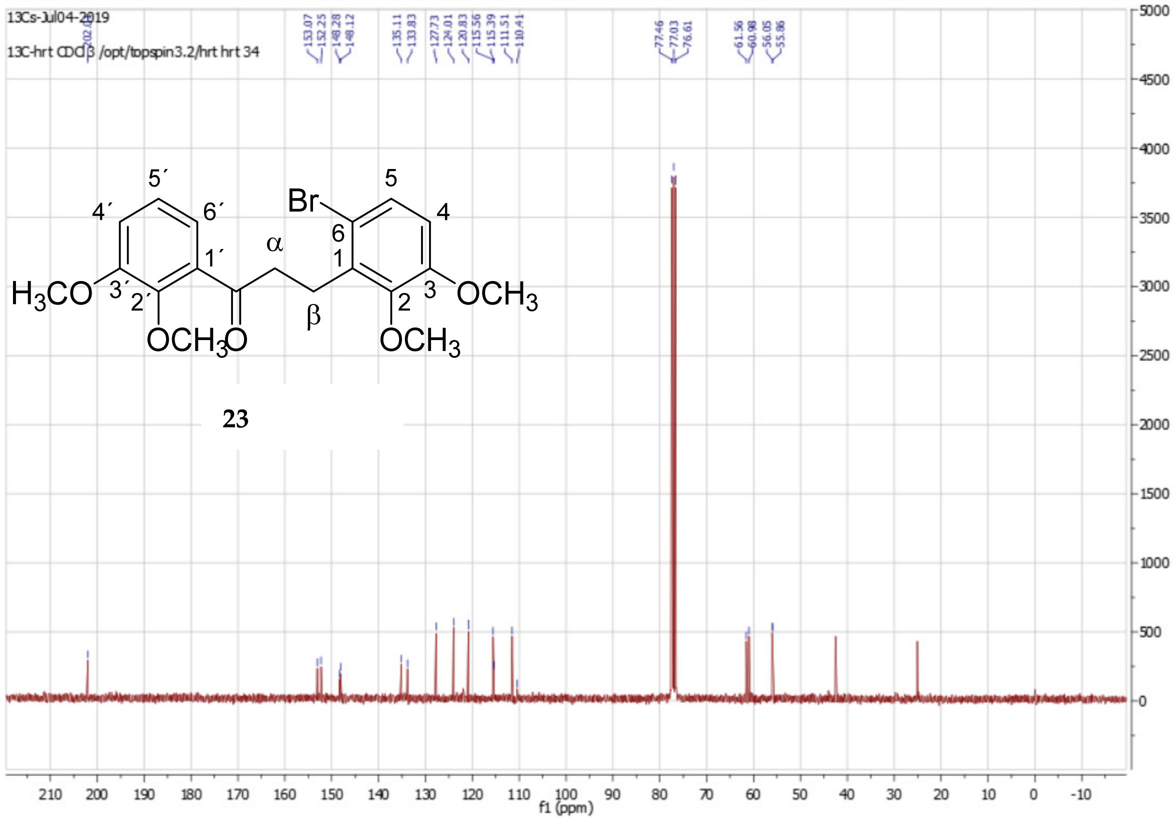

Figure S45. ^{1}H NMR and ^{13}C NMR spectra of compound **23**.

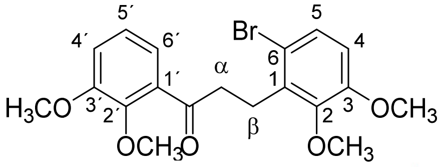

23

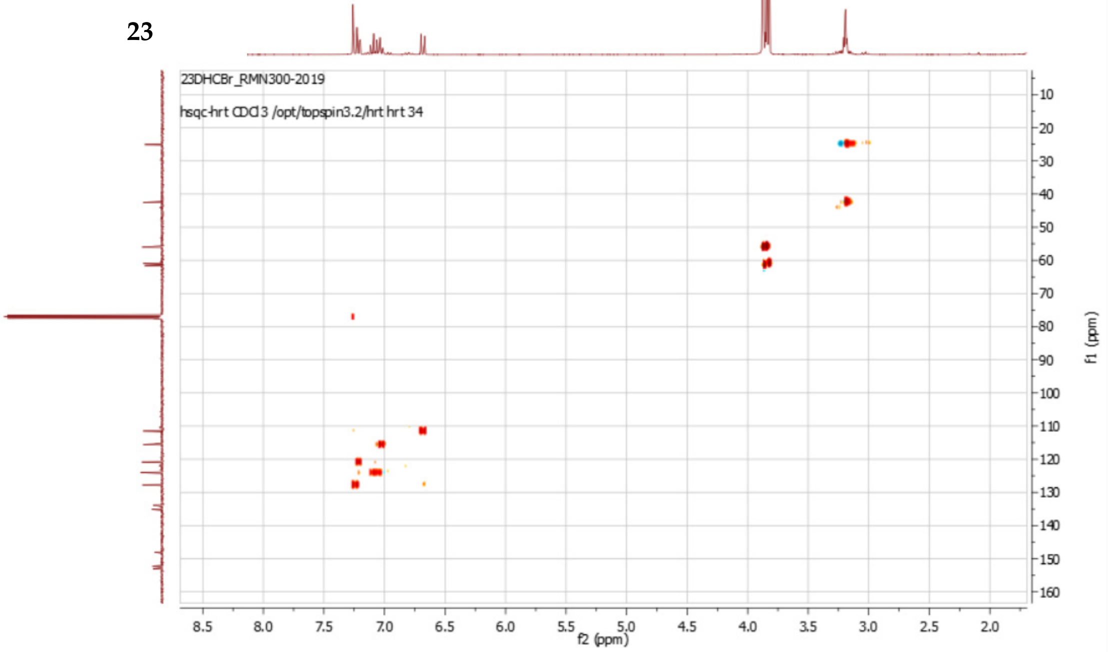

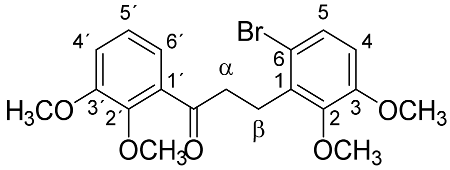

23

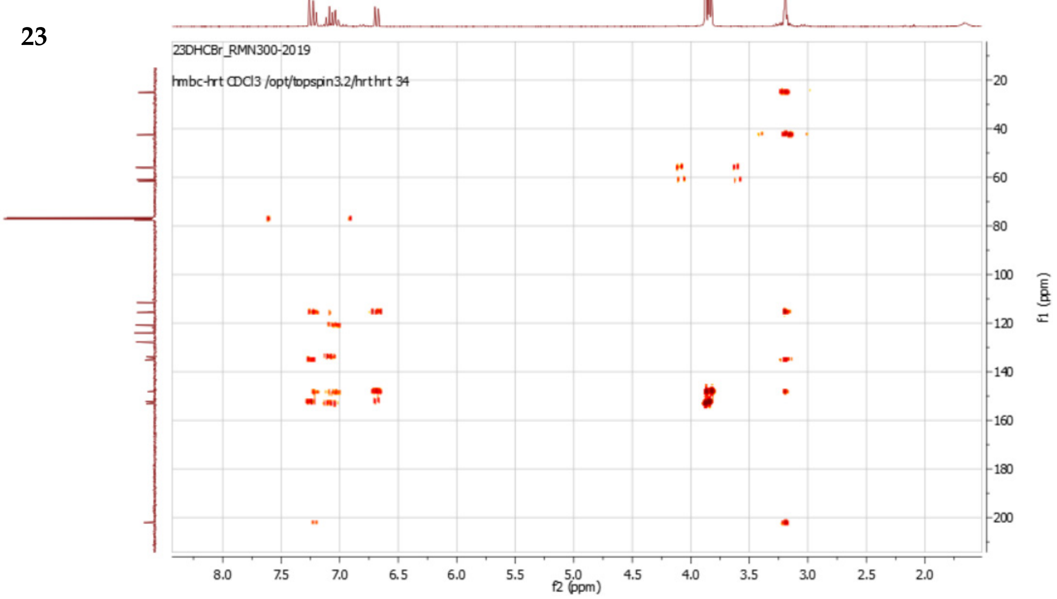

Figure S46. HSQC and HMBC spectra of compound **23**.

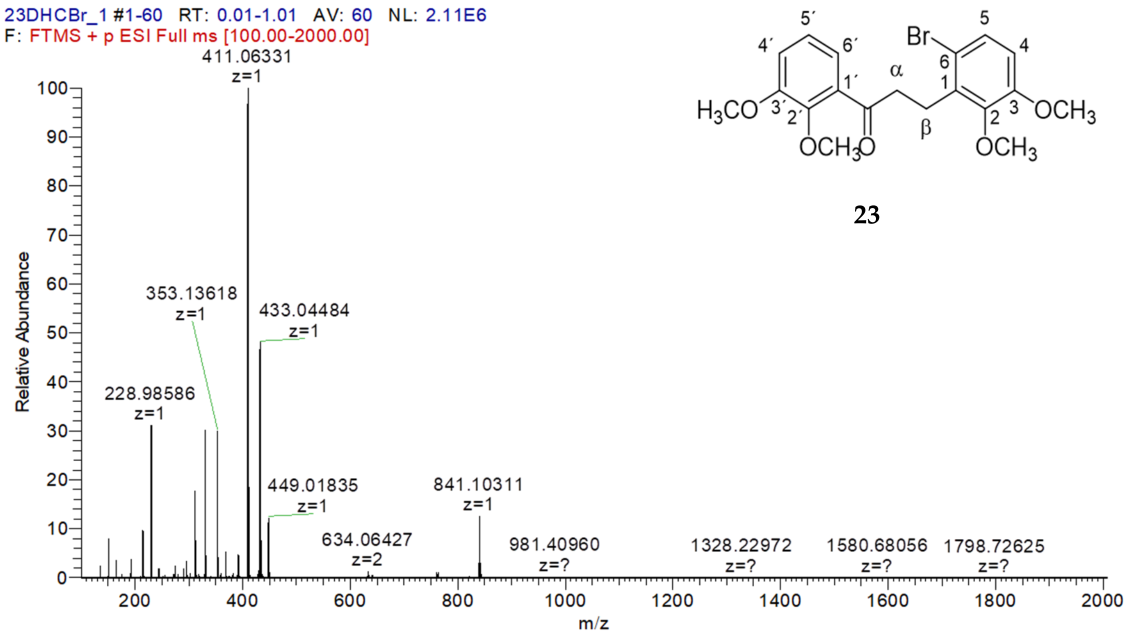

23

m/z calculated (ppm)	Formula	m/z found (ppm)	Error (ppm)
411.27600	$C_{19}H_{23}BrO_5$	411.06331	-0.21269

Figure S47. HRMS spectrum of compound **23**.

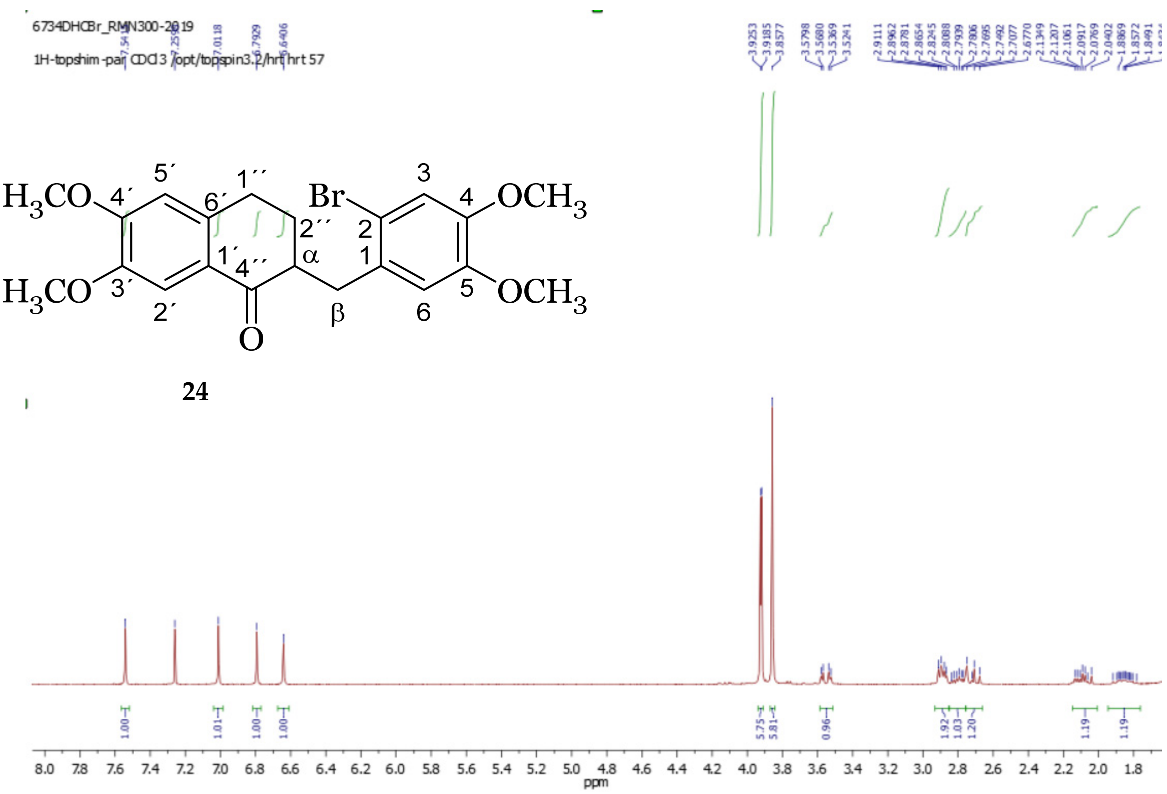

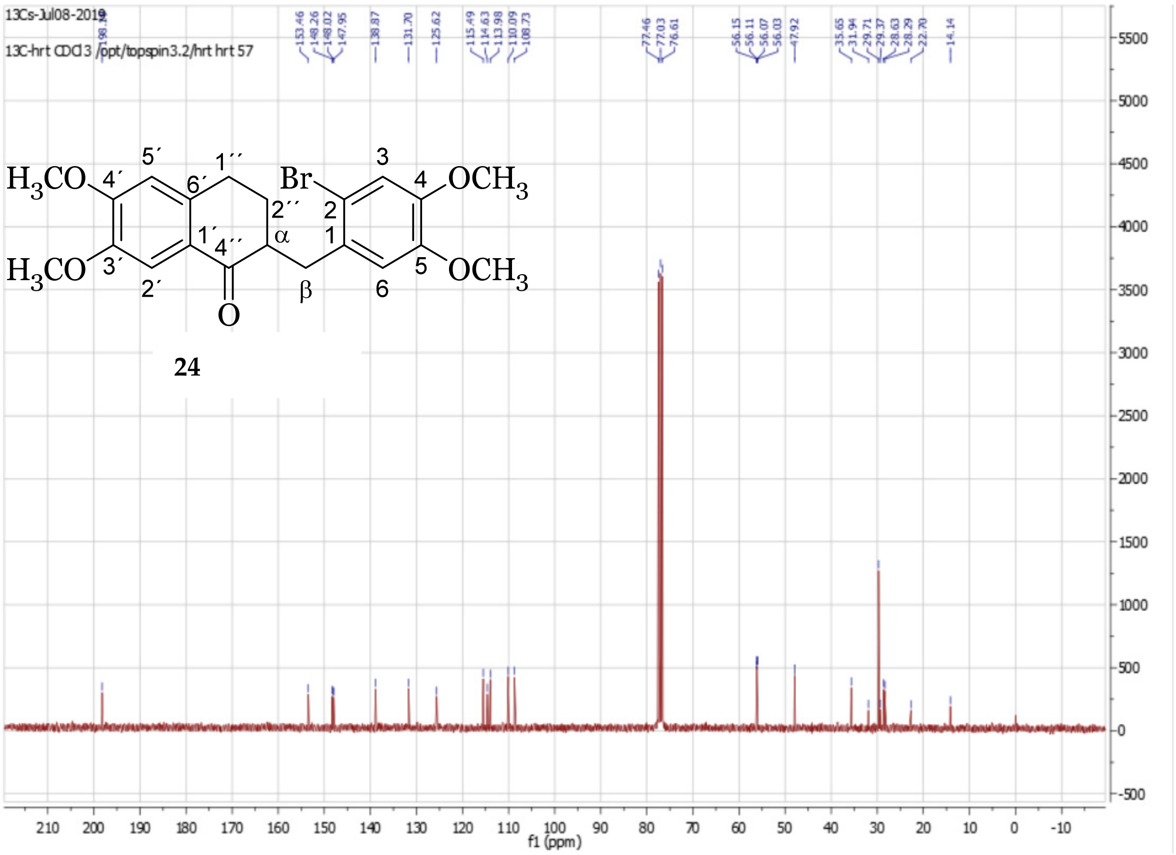

Figure S48. ^{1}H NMR and ^{13}C NMR spectra of compound **24**.

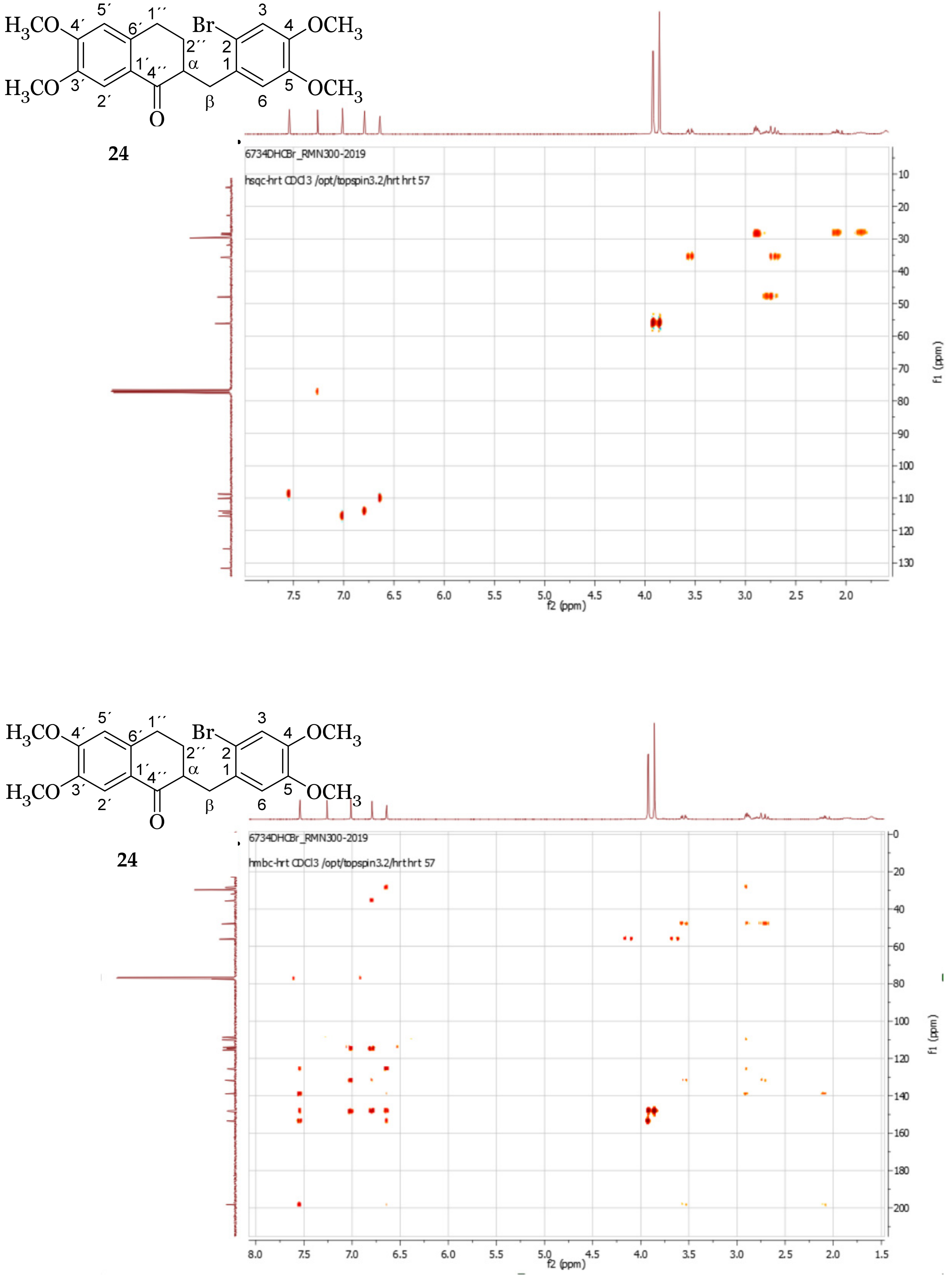

Figure S49. HSQC and HMBC spectra of compound **24**.

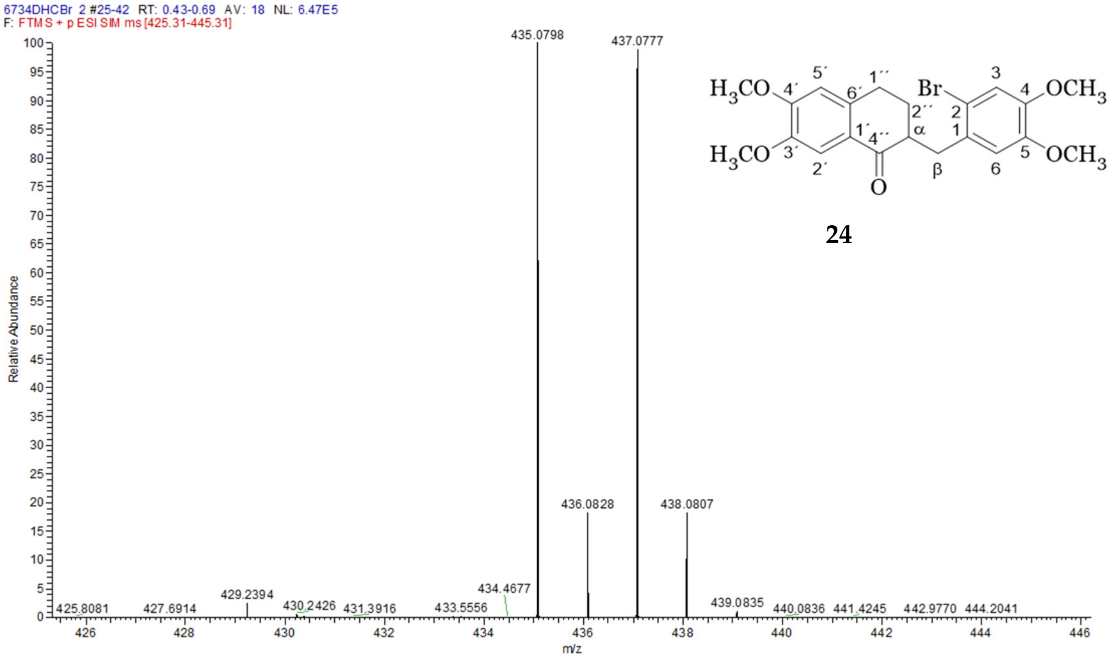

24

m/z calculated (ppm)	Formula	m/z found (ppm)	Error (ppm)
435.07289	$C_{21}H_{24}BrO_5$	435.0798	0.00691

Figure S50. HRMS spectrum of compound **24**.

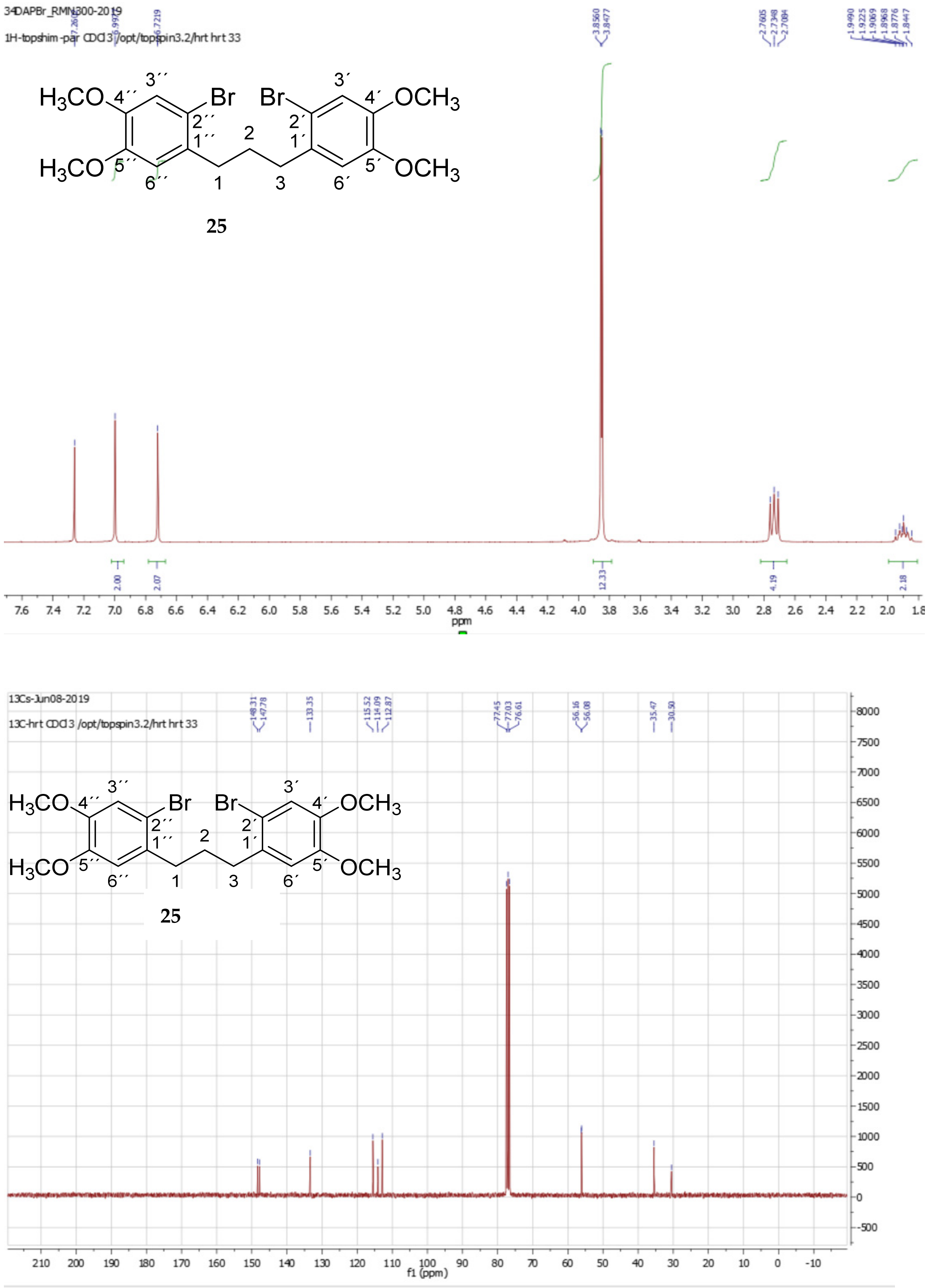

Figure S51. ^{1}H NMR and ^{13}C NMR spectra of compound **25**.

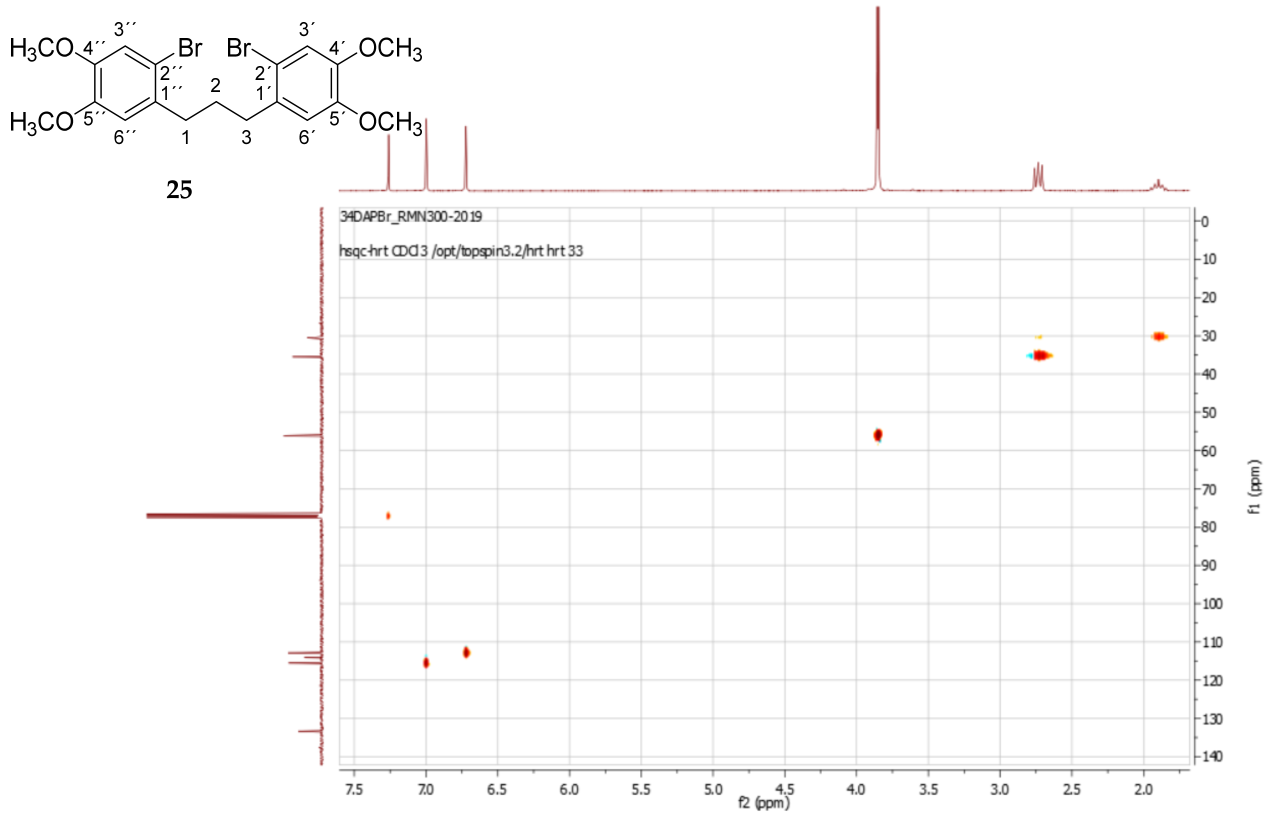

25

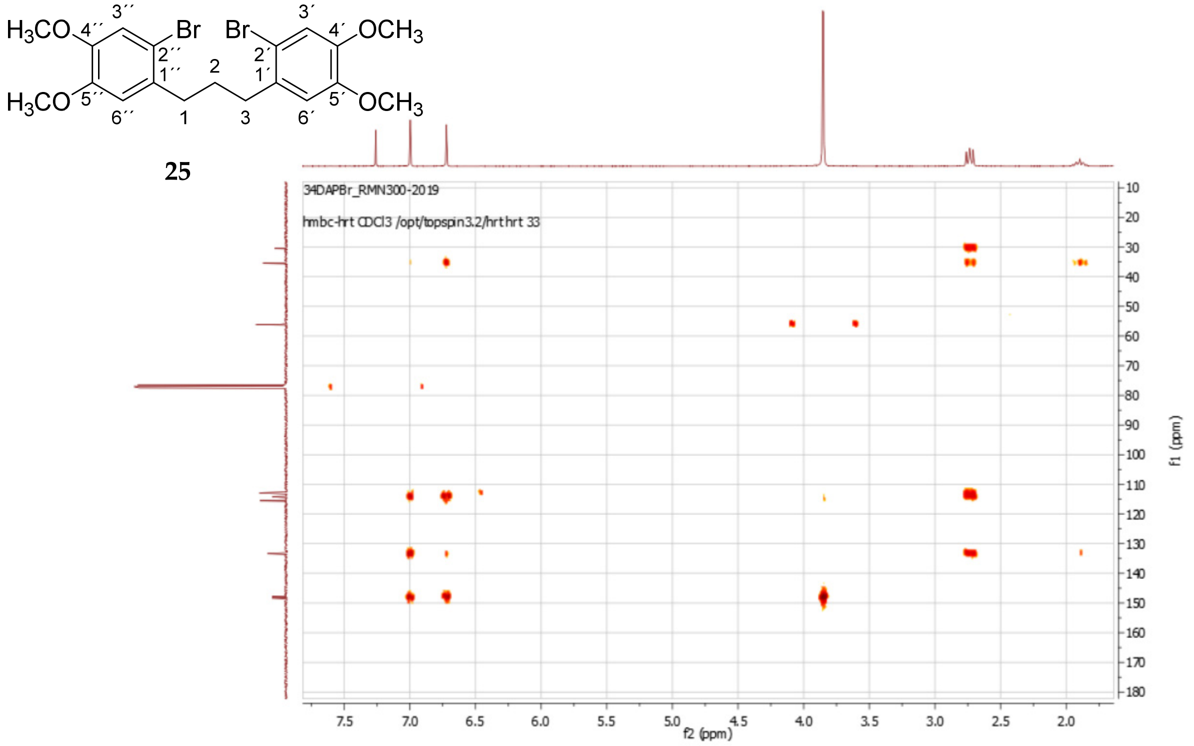

25

Figure S52. HSQC and HMBC spectra of compound **25**.

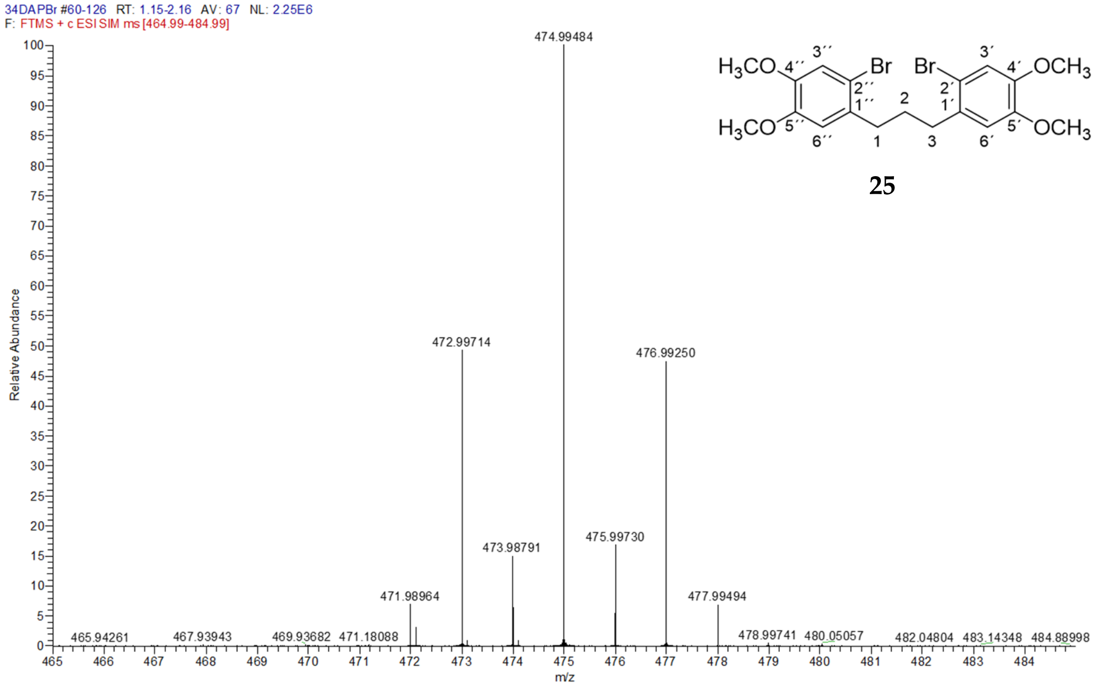

m/z calculated (ppm)	Formula	m/z found (ppm)	Error (ppm)
474.98644	$C_{19}H_{23}Br_2O_4$	474.99484	0.00840

Figure S53. HRMS spectrum of compound **25**.

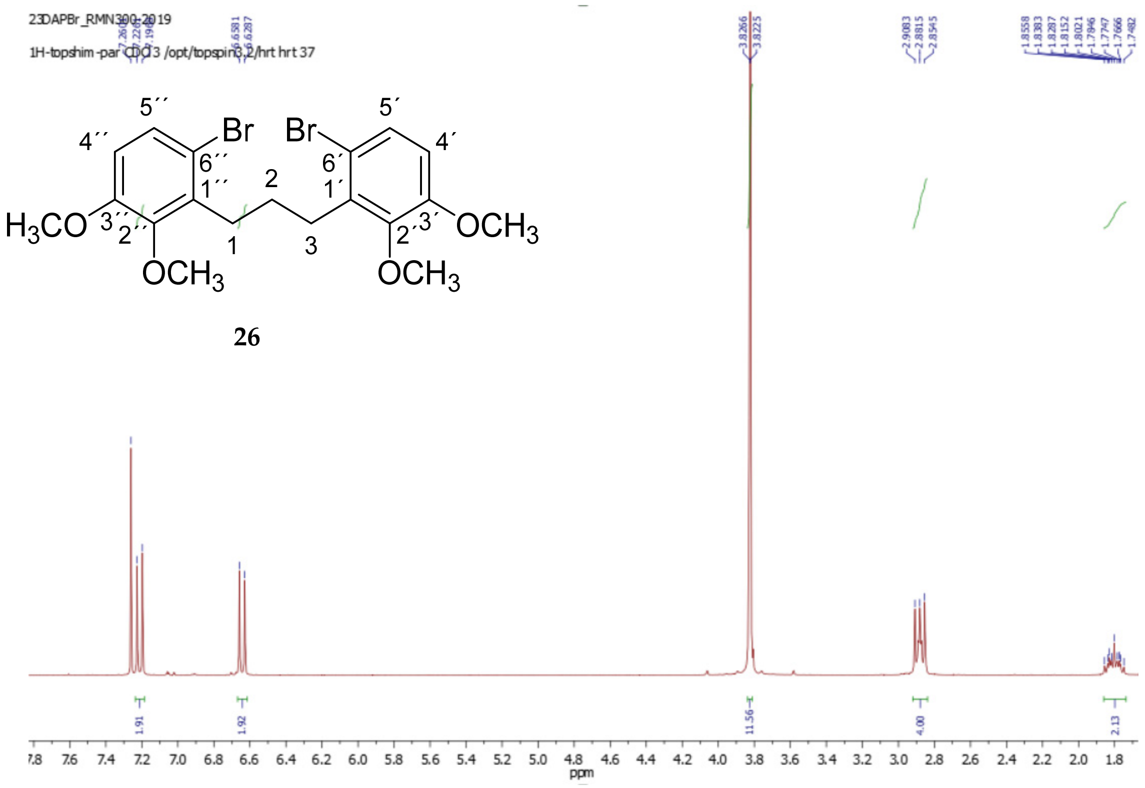

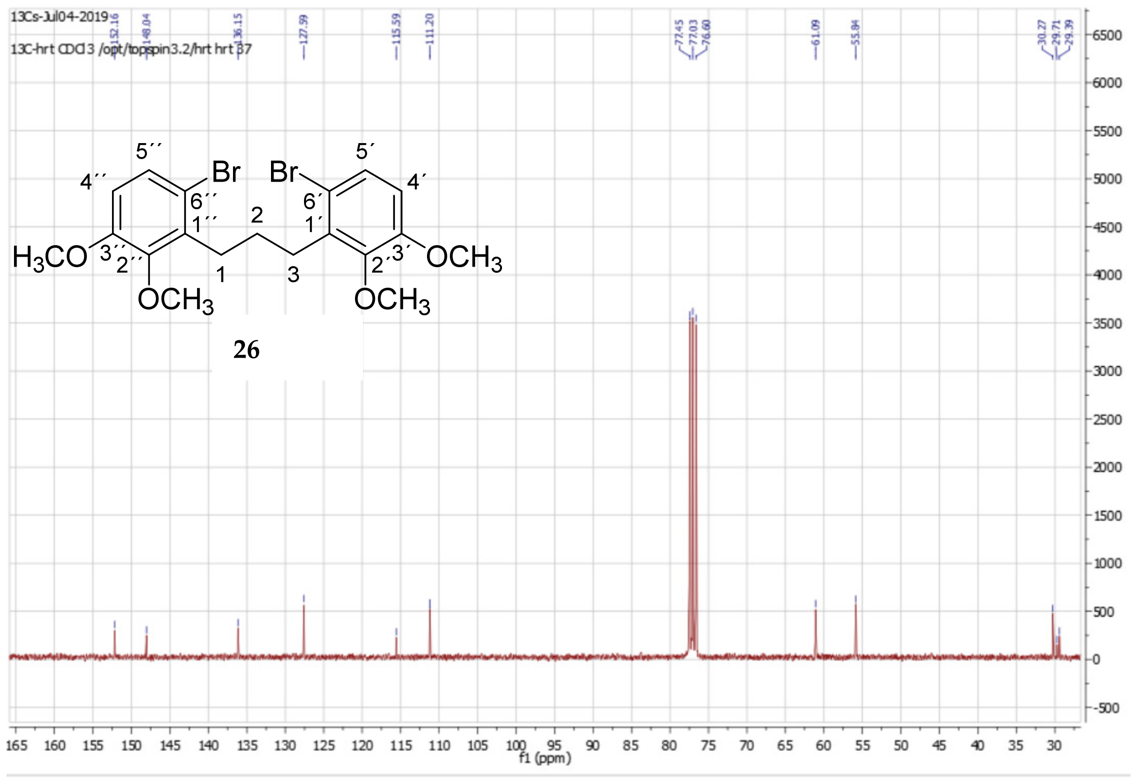

Figure S54. ^{1}H NMR and ^{13}C NMR spectra of compound **26**.

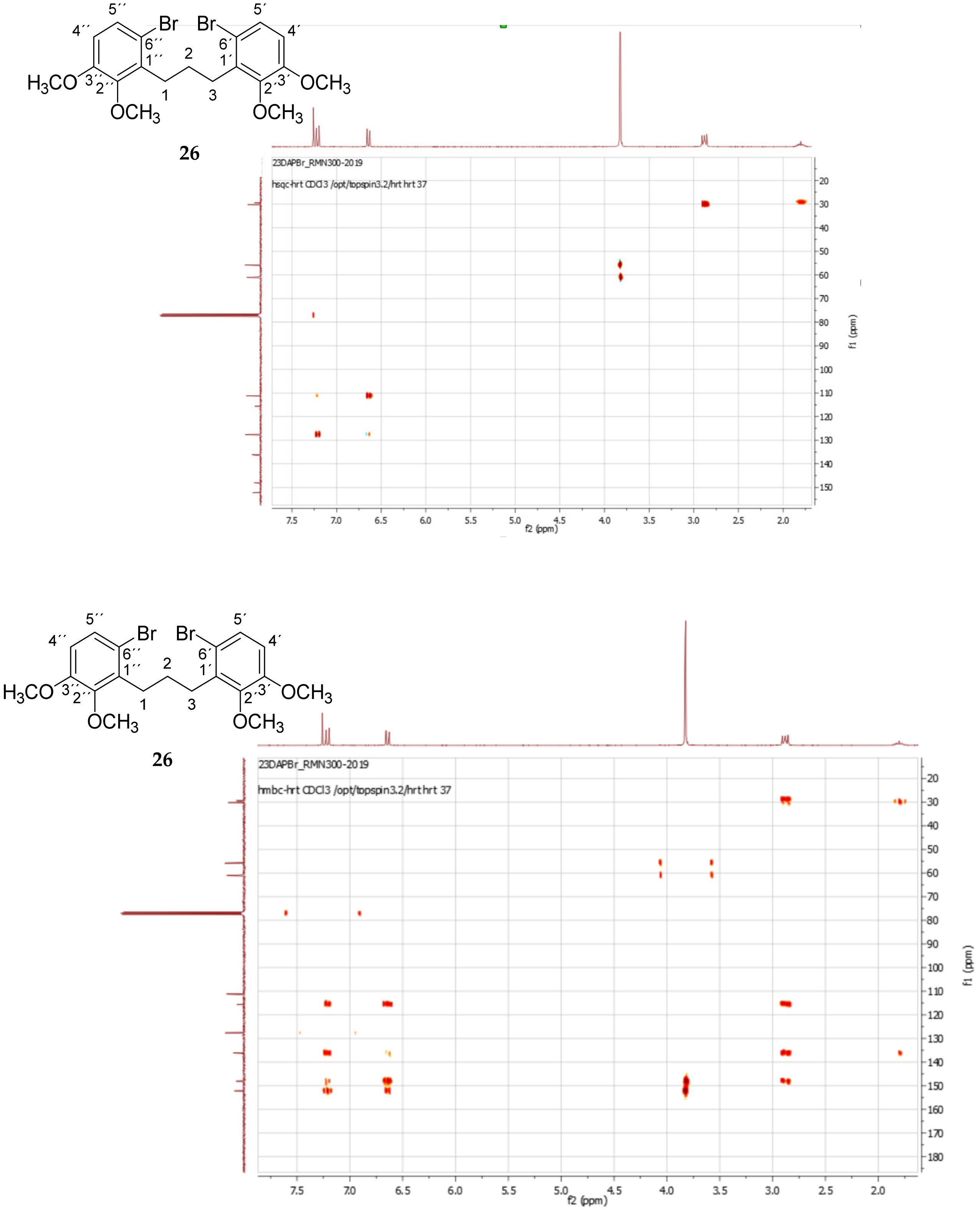

Figure S55. HSQC and HMBC spectra of compound **26**.

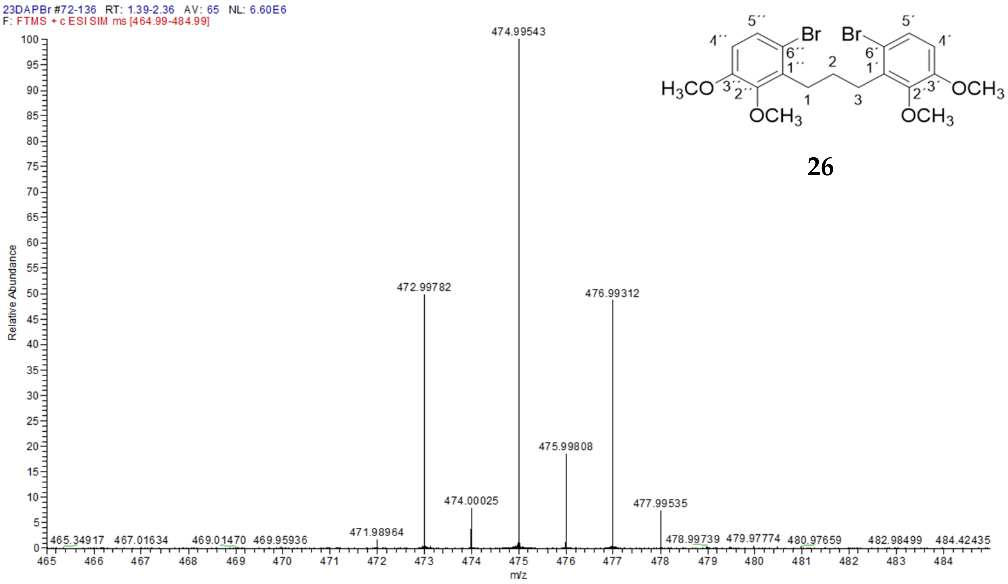

26

m/z calculated (ppm)	Formula	m/z found (ppm)	Error (ppm)
474.98644	$C_{19}H_{23}Br_2O_4$	474.99543	0,00899

Figure S56. HRMS spectrum of compound **26**.

Relative fluorescence index curves for compounds 9 – 26

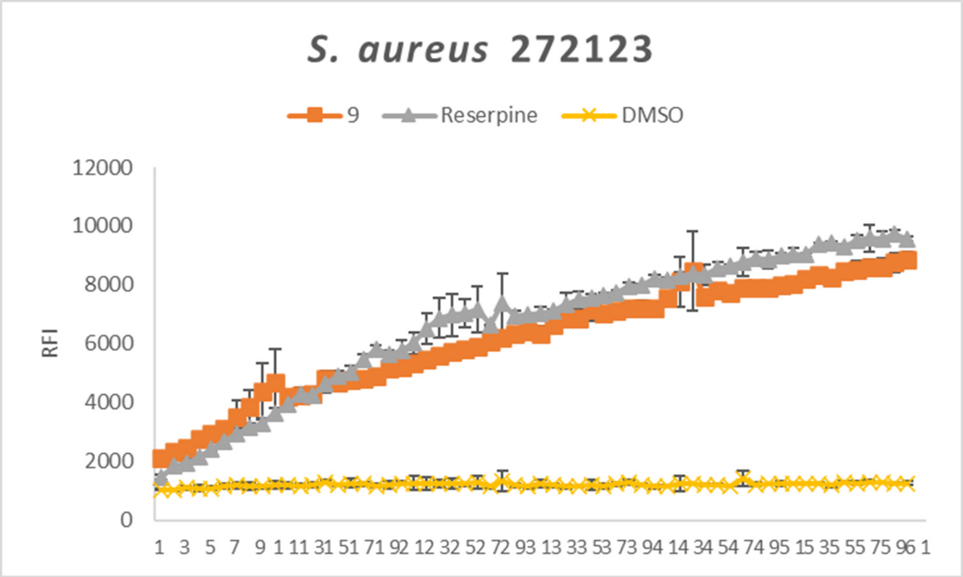

Figure S57. Relative fluorescence index (RFI) of compound **9**, reserpine (positive control) and DMSO (negative control).

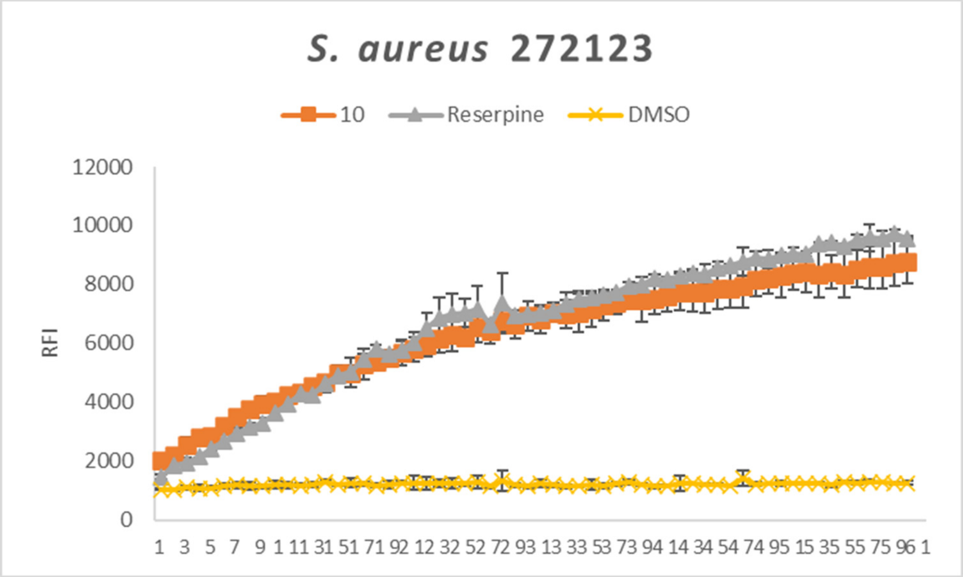

Figure S58. RFI of compound **10**, reserpine (positive control) and DMSO (negative control).

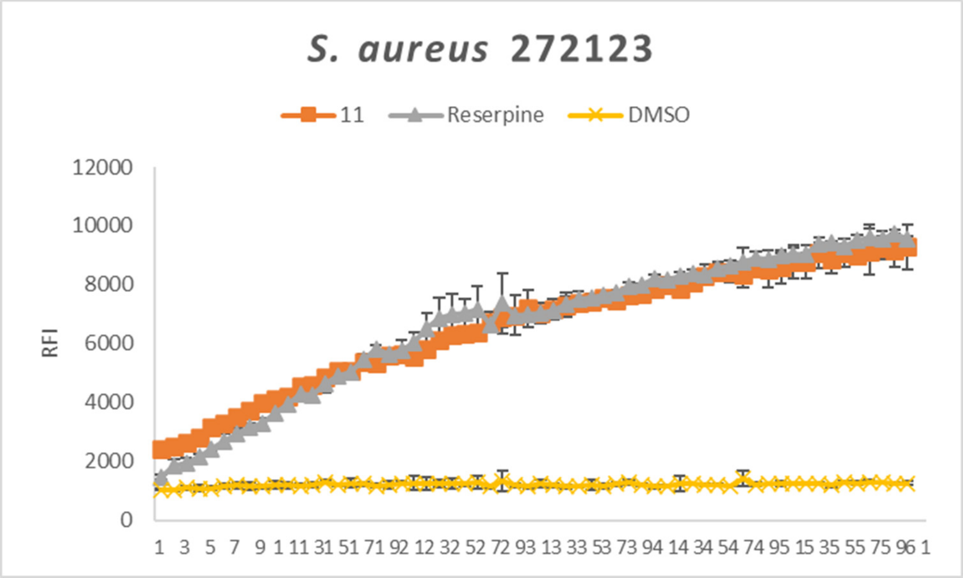

Figure S59. RFI of compound **11**, reserpine (positive control) and DMSO (negative control).

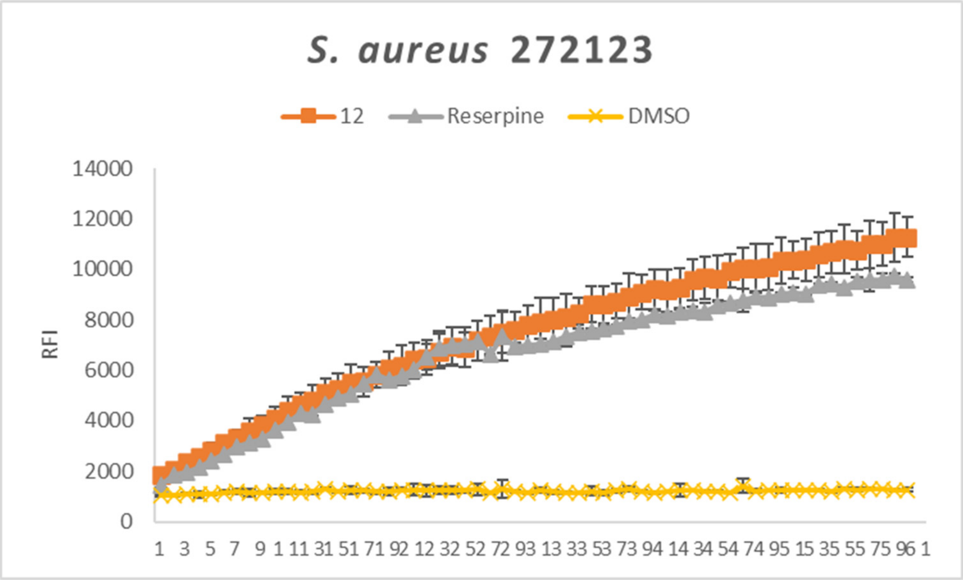

Figure S60. RFI of compound **12**, reserpine (positive control) and DMSO (negative control).

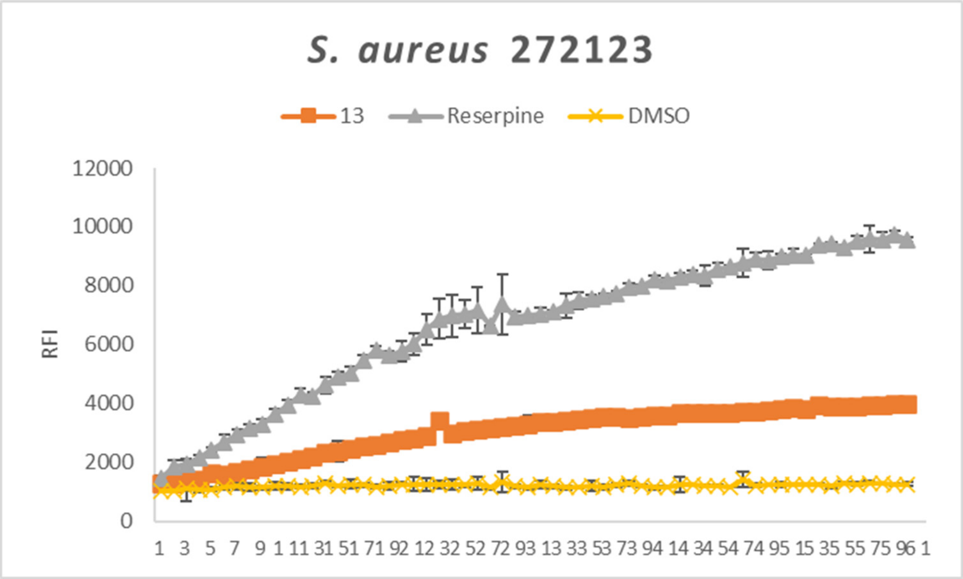

Figure S61. RFI of compound **13**, reserpine (positive control) and DMSO (negative control).

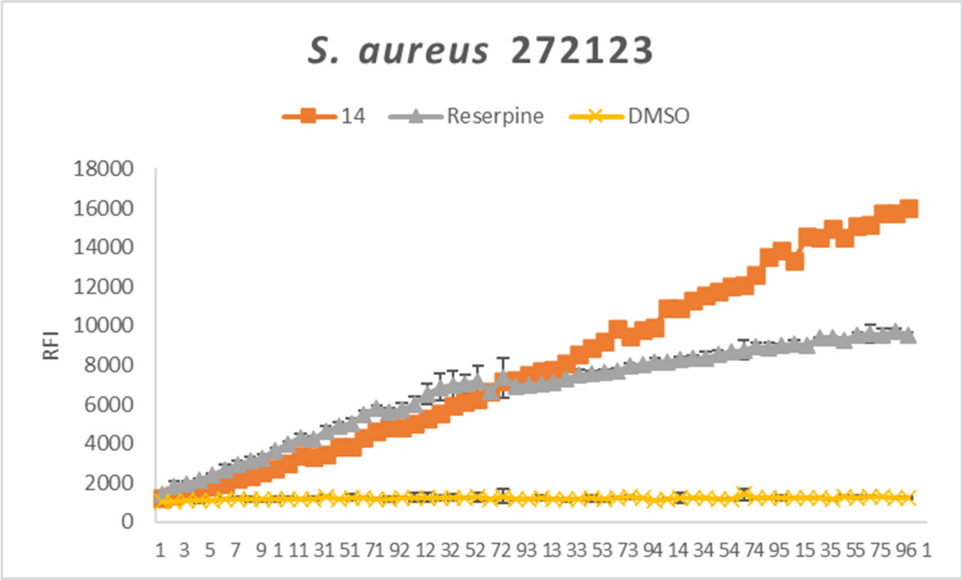

Figure S62. RFI of compound **14**, reserpine (positive control) and DMSO (negative control).

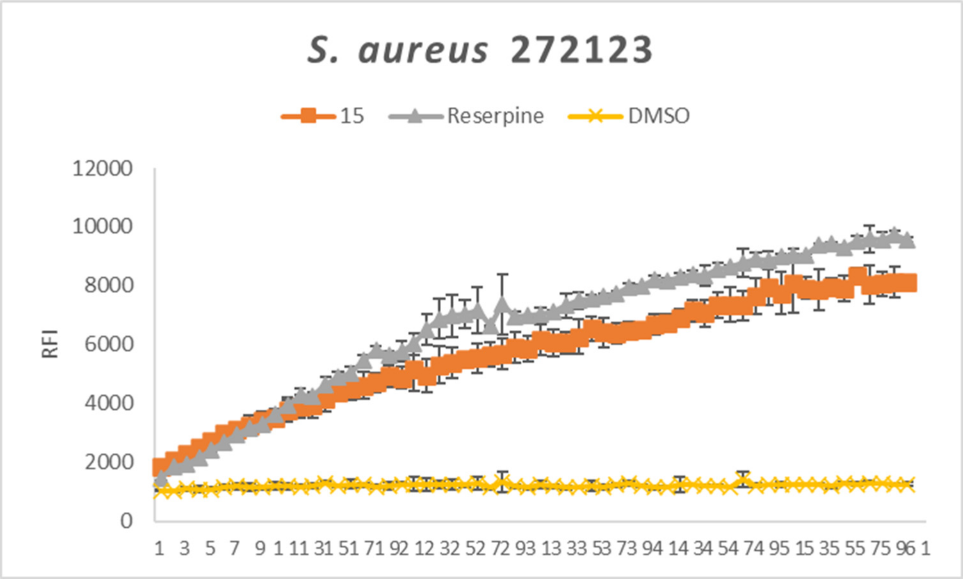

Figure S63. RFI of compound **15**, reserpine (positive control) and DMSO (negative control).

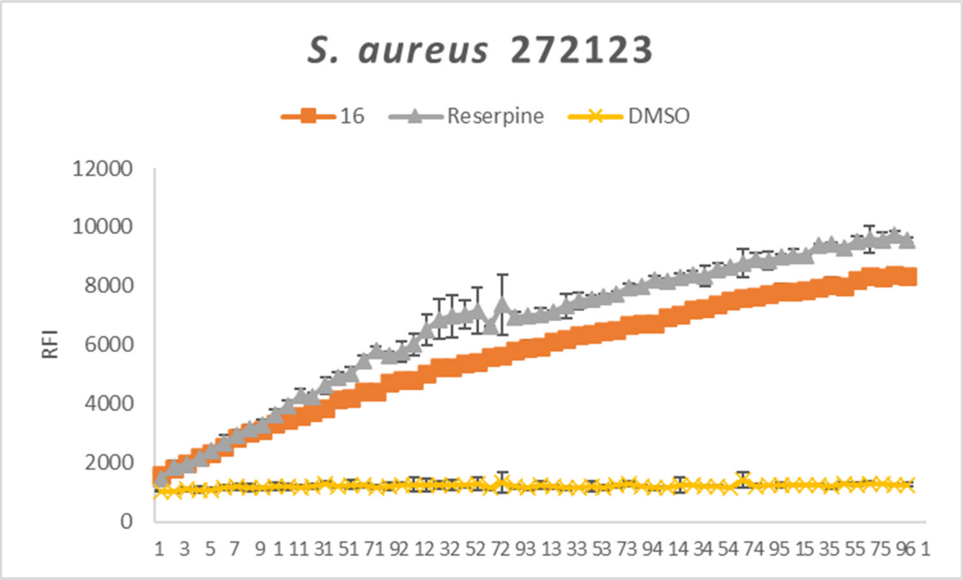

Figure S64. RFI of compound **16**, reserpine (positive control) and DMSO (negative control).

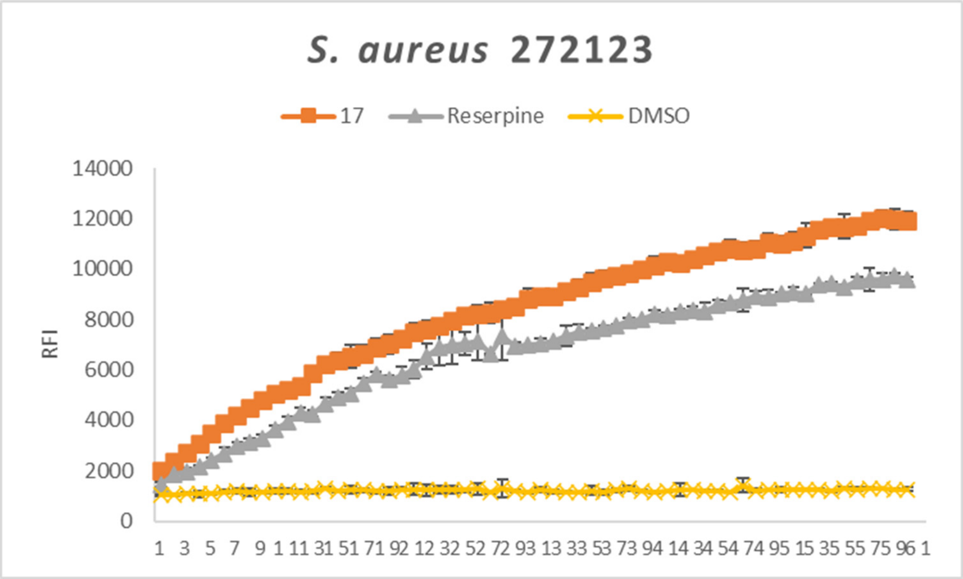

Figure S65. RFI of compound **17**, reserpine (positive control) and DMSO (negative control).

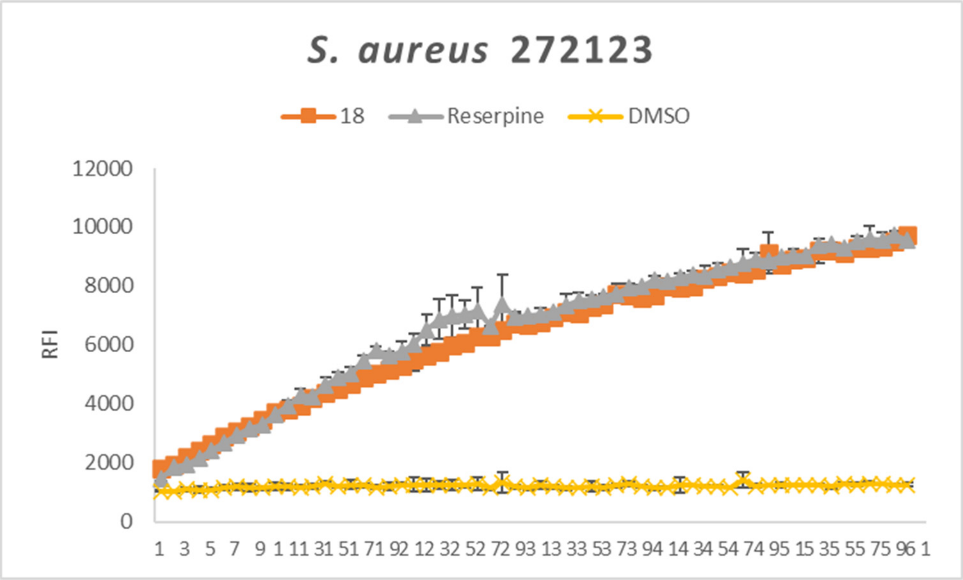

Figure S66. RFI of compound **18**, reserpine (positive control) and DMSO (negative control).

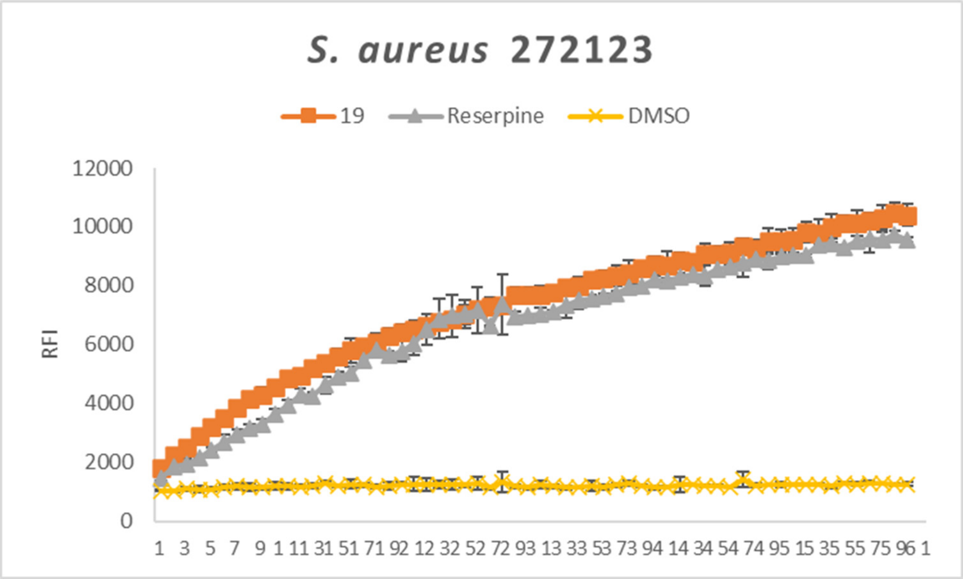

Figure S67. RFI of compound **19**, reserpine (positive control) and DMSO (negative control).

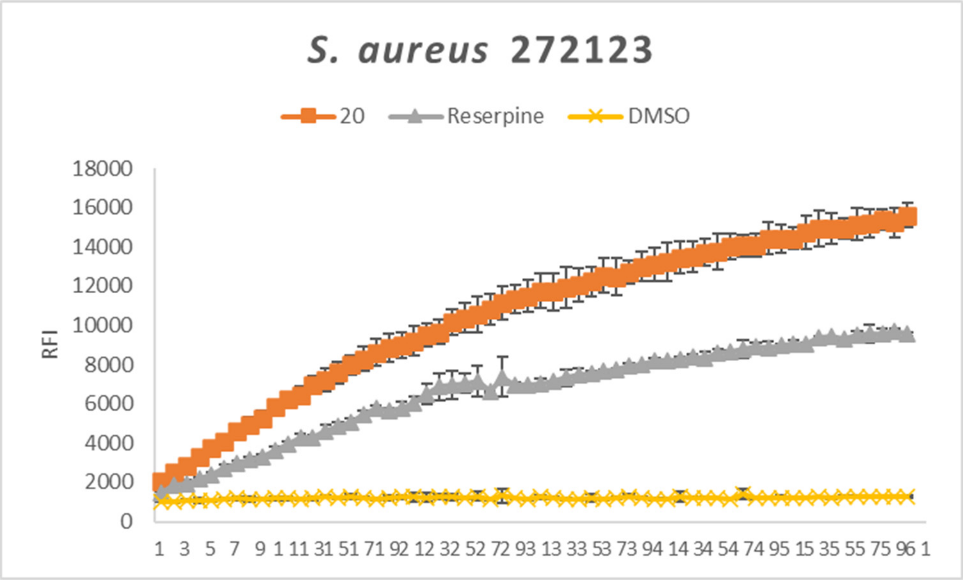

Figure S68. RFI of compound **20**, reserpine (positive control) and DMSO (negative control).

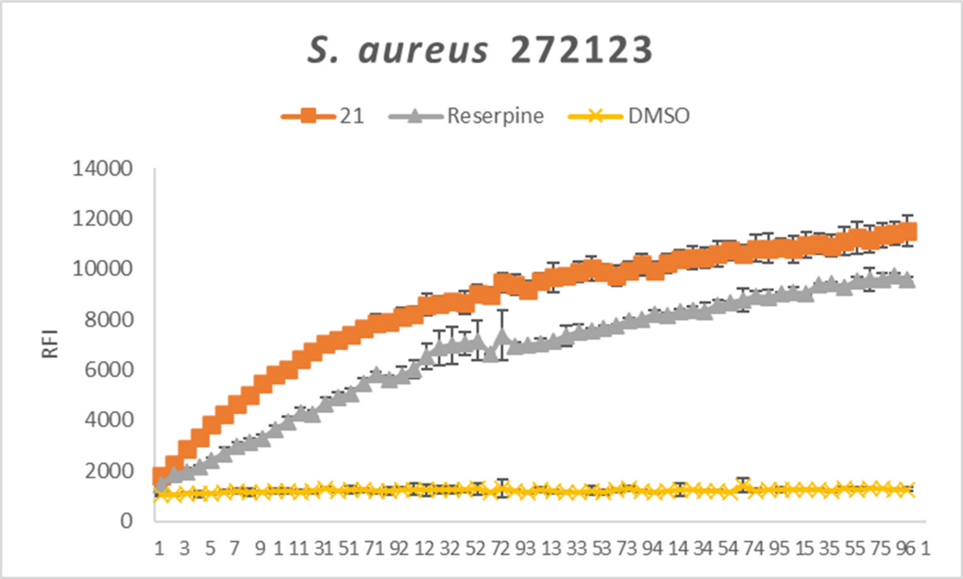

Figure S69. RFI of compound **21**, reserpine (positive control) and DMSO (negative control).

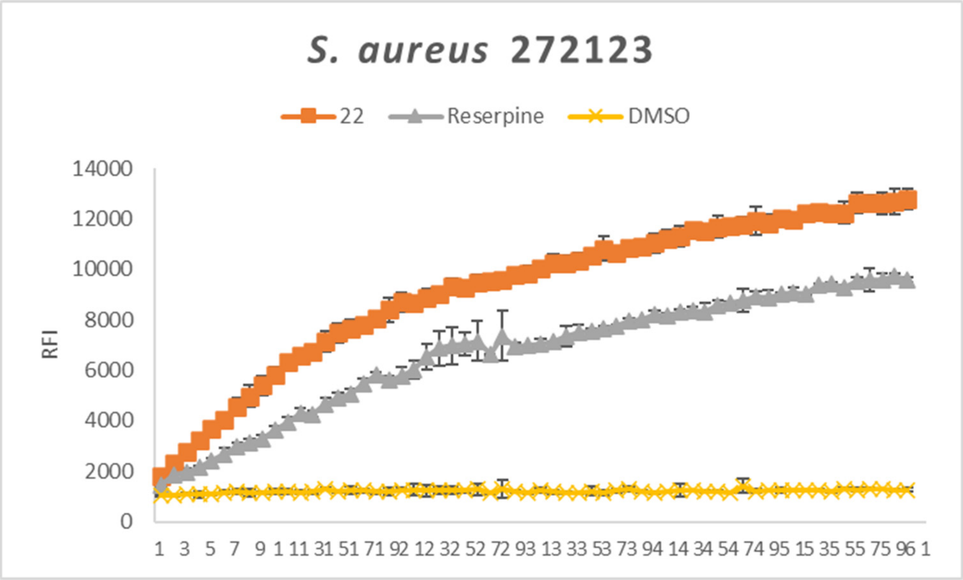

Figure S70. RFI of compound **22**, reserpine (positive control) and DMSO (negative control).

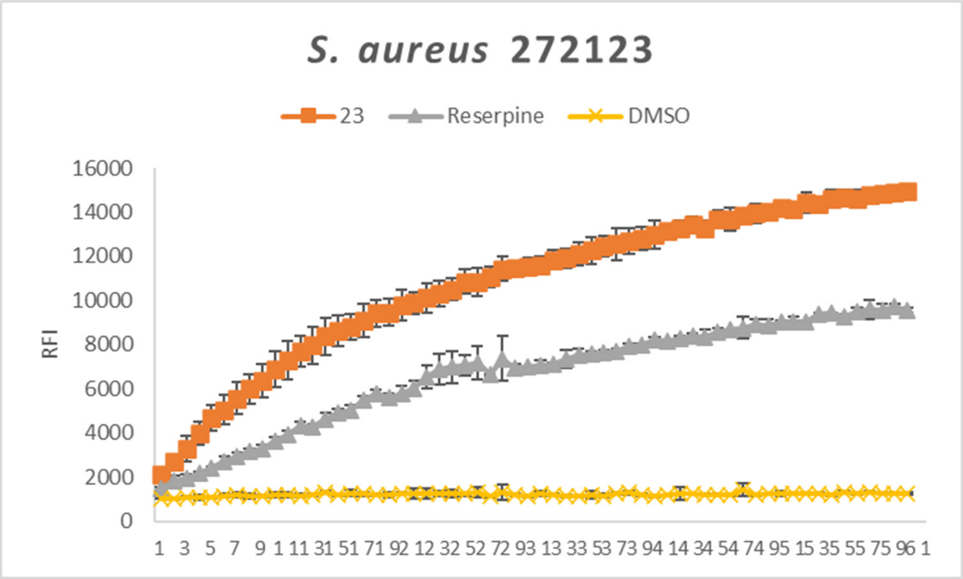

Figure S71. RFI of compound **23**, reserpine (positive control) and DMSO (negative control).

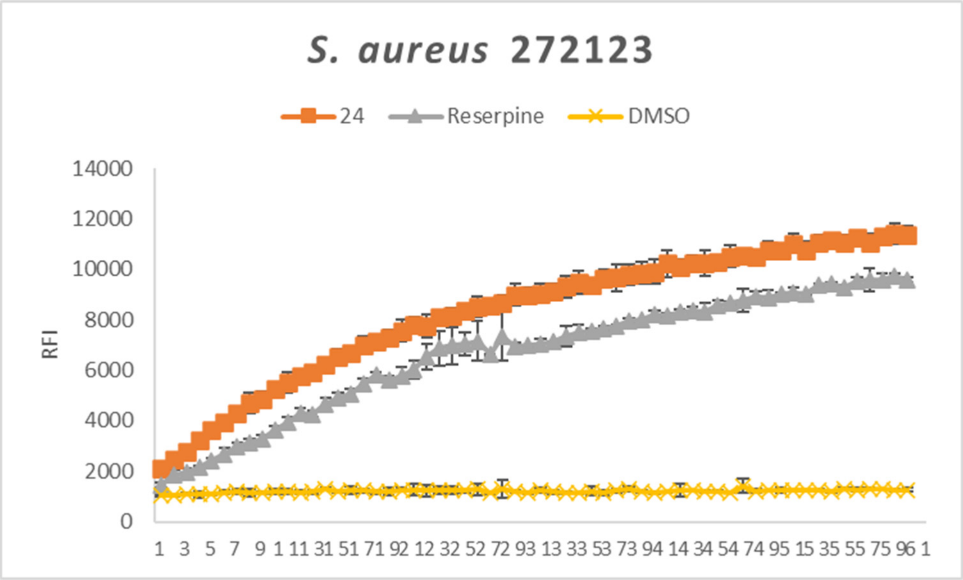

Figure S72. RFI of compound **24**, reserpine (positive control) and DMSO (negative control).

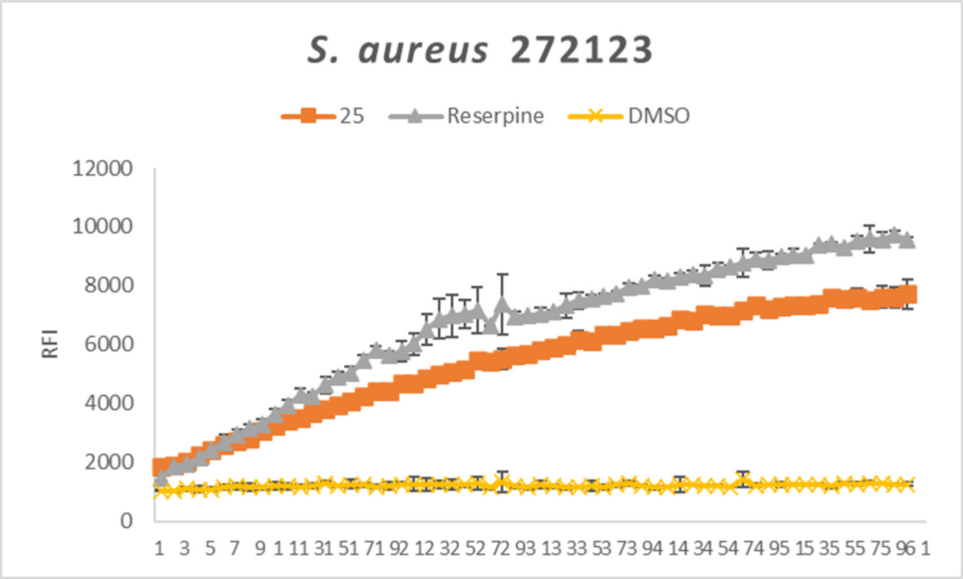

Figure S73. RFI of compound **25**, reserpine (positive control) and DMSO (negative control).

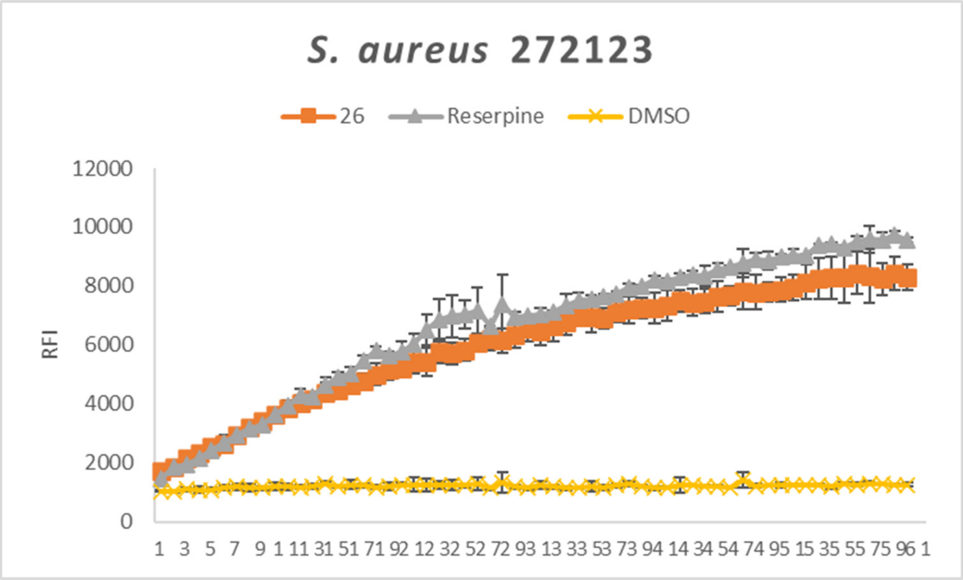

Figure S74. RFI of compound **26**, reserpine (positive control) and DMSO (negative control).

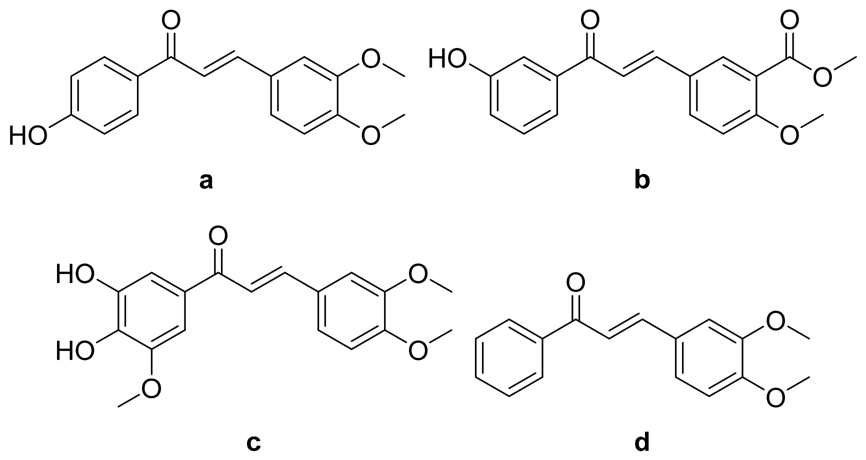

Figure S75. Chalcones described in [10], used in the docking studies.

Docking studies

Docking studies were performed in the NorA homology model, and the sites used for docking of the compounds were the binding core region (BCR) and the cytoplasmic side (CS), as described in [28] (**Table S1**).

Table S1. Docking results for the compounds in NorA (BCR: binding core region; CS: cytoplasmic side).

**Name**	**Docking Score**	
	**BCR**	**CS**
Reserpine	1.0	-4.6
**9**	-5.5	-5.2
**10**	-7.5	-5.6
**11**	-4.5	-4.8
**12**	-5.3	-5.3
**13**	-7.7	-7.3
**14**	-6.3	-5.2
**15**	-5.7	-5.6
**16**	-7.6	-7.5
**17**	-6.9	-5.1
**18**	-6.8	-5.6
**22**	-6.8	-6.8
**23**	-5.0	-4.9

**24**	-5.4	-5.6
**19**	-7.5	-7.4
**20**	-6.8	-5.1
**21**	-7.4	-7.2
**25**	-6.2	-6.1
**26**	-4.8	-4.5
**a**	-8.2	-4.4
**b**	-9.0	-4.6
**c**	-7.5	-4.9
**d**	-8.5	-4.6

From the analysis of the docking results, it can be noted that all the synthesized compounds are predicted to bind more effectively than reserpine in the sites tested, suggesting a possible potential for these compounds to act as EP inhibitors. However, it must be taken into account that a homology model of NorA is being used for the docking studies, and the results may not reflect the actual activity of the compounds. Nevertheless, all the compounds were tested for their potential as EP inhibitors.

Reference:
10. Rezende-Junior, L.M.; Andrade, L.M.S.; Leal, A.; Mesquita, A.B.S.; Santos, A.; Neto, J.S.L.; Siqueira-Junior, J.P.; Nogueira, C.E.S.; Kaatz, G.W.; Coutinho, H.D.M.; et al. Chalcones Isolated from Arrabidaea brachypoda Flowers as Inhibitors of NorA and MepA Multidrug Efflux Pumps of Staphylococcus aureus. *Antibiotics (Basel)* **2020**, *9*, doi:10.3390/antibiotics9060351.
